# Directed Design of a Au^I^ Complex with a Reduced Mesoionic Carbene Radical Ligand: Insights from 1,2,3‐Triazolylidene Selenium Adducts and Extensive Electrochemical Investigations

**DOI:** 10.1002/chem.202100105

**Published:** 2021-03-17

**Authors:** Julia Beerhues, Maren Neubrand, Sebastian Sobottka, Nicolás I. Neuman, Hannes Aberhan, Shubhadeep Chandra, Biprajit Sarkar

**Affiliations:** ^1^ Lehrstuhl für Anorganische Koordinationschemie Institut für Anorganische Chemie Universität Stuttgart Pfaffenwaldring 55 70569 Stuttgart Germany; ^2^ Institut für Chemie und Biochemie Freie Universität Berlin Fabeckstraße 34–36 14195 Berlin Germany

**Keywords:** carbene–selenium adducts, cyclic voltammetry, mesoionic carbenes, radicals, spectroelectrochemistry

## Abstract

Carbene‐based radicals are important for both fundamental and applied chemical research. Herein, extensive electrochemical investigations of nine different 1,2,3‐triazolylidene selenium adducts are reported. It is found that the half‐wave potentials of the first reduction of the selones correlate with their calculated LUMO levels and the LUMO levels of the corresponding triazolylidene‐based mesoionic carbenes (MICs). Furthermore, unexpected quasi‐reversibility of the reduction of two triazoline selones, exhibiting comparable reduction potentials, was discovered. Through UV/Vis/NIR and EPR spectroelectrochemical investigations supported by DFT calculations, the radical anion was unambiguously assigned to be triazoline centered. This electrochemical behavior was transferred to a triazolylidene‐type MIC‐gold phenyl complex resulting in a MIC‐radical coordinated Au^I^ species. Apart from UV‐Vis‐NIR and EPR spectroelectrochemical investigations of the reduction, the reduced gold‐coordinated MIC radical complex was also formed in situ in the bulk through chemical reduction. This is the first report of a monodentate triazolylidene‐based MIC ligand that can be reduced to its anion radical in a metal complex. The results presented here provide design principles for stabilizing radicals based on MICs.

## Graphical abstract

**MIC drop**! The first example and the spectroscopic characterization of a metal‐bound as well as selenium‐bound mesoionic carbene (MIC)‐radical of the 1,2,3‐triazol‐5‐ylidene type is reported. Design principles for stabilizing such radicals are presented as well.

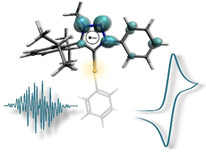

## Introduction

Triazolylidenes are powerful and versatile ligands in transition‐metal coordination chemistry.^[1]^ The readily available and widely variable ligand precursors, obtained from copper‐catalyzed azide‐alkyne cycloaddition, and the higher donor ability compared with classic N‐heterocyclic carbenes (NHCs) has led to the popularity of this ligand type in the last years. Surprisingly, the research field of triazolylidenes with main group elements is relatively unexplored, even though research of main group elements with classical NHCs and among them the group of cyclic alkyl(amino) carbenes (CAACs) is much more advanced.^[2]^ It was shown that they, inter alia, stabilize low valent and low coordinate main group elements and radical species.^[3]^ The few existing publications on 1,2,3‐triazolylidenes as actors in main group chemistry indicate promising results for further studies.^[4]^

Recently, we investigated the π‐accepting properties of triazolylidenes in selenium adducts^[5]^ by the method by Ganter and co‐workers who introduced ^77^Se NMR spectroscopic investigations of those adducts for this purpose.^[6]^ Herein, we shed light on the electrochemical behavior of this relatively new substance class. It is the first time that these triazoline selones are analyzed electrochemically. During that process, we discovered several interesting properties of these triazoline selones, of the corresponding mesoionic carbenes (MICs), and of Au^I^ complexes of the MICs.

This work displays a journey starting with the aim to determine the electronic properties of triazolylidenes through electrochemical investigation of their easily available selenium adducts and finding spectroscopic evidence of a reduced triazolylidene gold complex in bulk. Additionally, we report on DFT‐driven directed design of radical anion stabilizing triazolylidene transition‐metal complexes through triazoline selones. Intermediate stops are cyclic voltammetric investigations of the selones, correlation of reduction potentials to LUMO energy levels, unexpected electrochemical reversibility of specific triazoline selones, the analysis of the reduced species, and transfer of the electrochemical properties from a MIC selenium adduct to a transition‐metal complex. Cyclic voltammetry, EPR, and UV/Vis spectroelectrochemical investigations, theoretical calculations and synthetic methods line the way. To the best of our knowledge, this works represents the first report of the reversible reduction of a 1,2,3‐triazol‐5‐ylidene ligand to the corresponding radical anion. Furthermore, we also investigate the electrochemically driven cleavage of Au^I^−Cl bonds, a process that is immensely important in homogeneous catalysis with Au^I^ complexes.

## Results and Discussion

### Cyclic voltammetric investigations of triazoline selones: Start of the investigations

**Figure 1:**
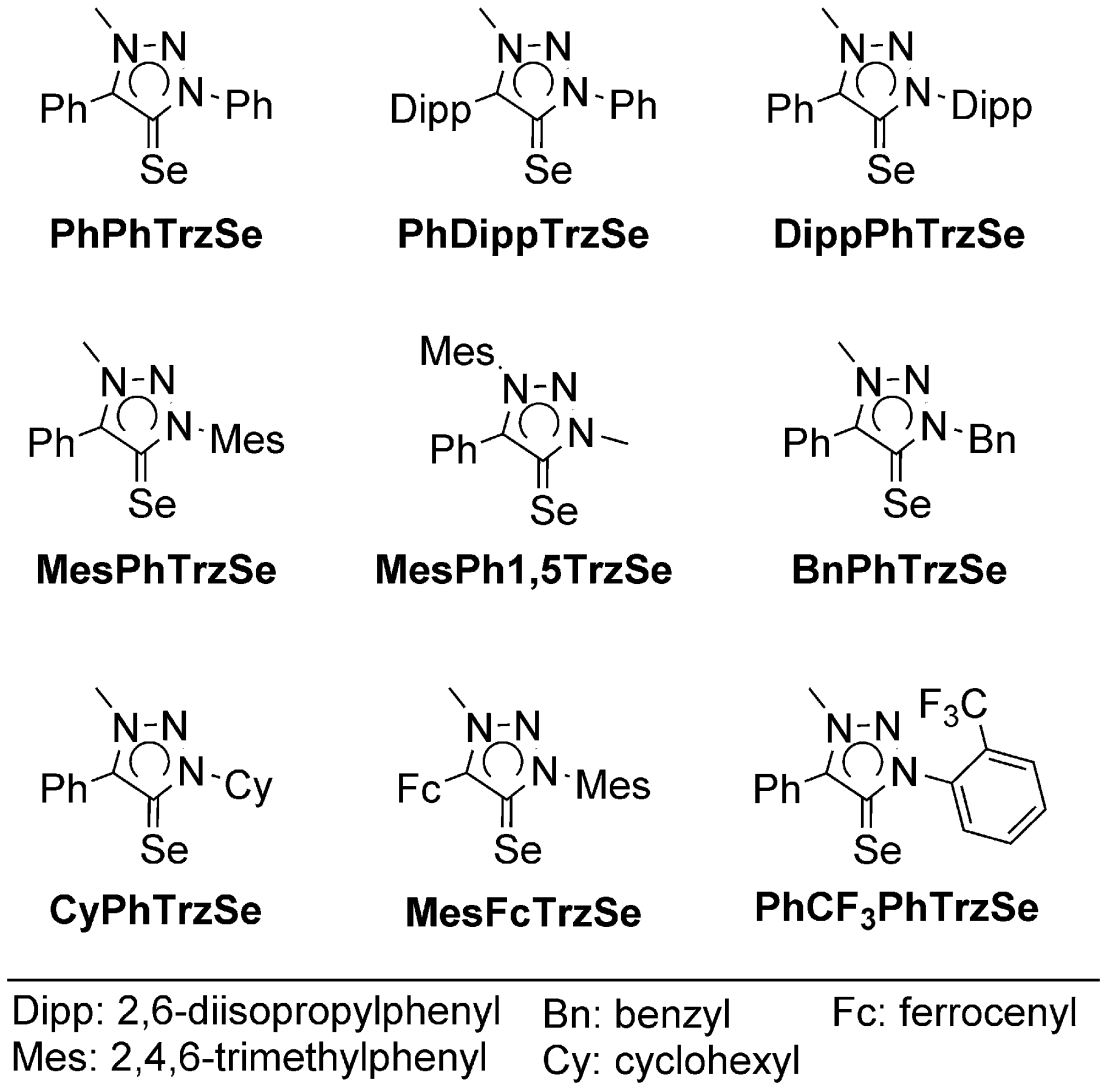
Triazolylidene selenium adducts studied electrochemically in this work.

**Figure 2:**
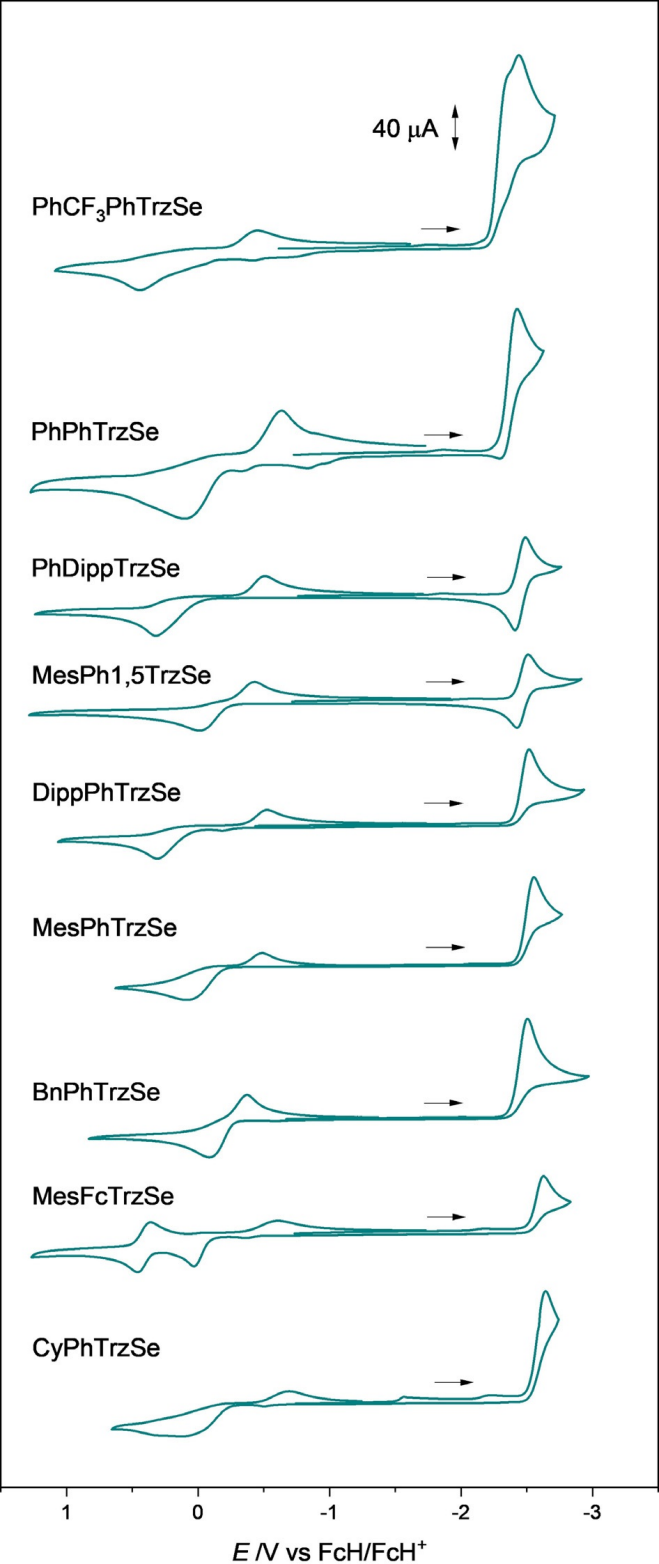
Cyclic voltammograms of the investigated triazoline selones (scan rate: 0.1 V s^−1^, in MeCN, electrolyte: NBu_4_PF_6_ (0.1 m), electrode: glassy carbon).

A series of triazolylidene selenium adducts was analyzed electrochemically (Figure 1). The synthesis and analytics can be found in a recent publication by our group.^[5]^ Cyclic voltammograms (CVs) of all the triazoline selones were recorded (Figure 2). Through the straightforward and inexpensive availability of the selones, a relatively wide range of differently substituted species could easily be analyzed.

**Scheme 1:**
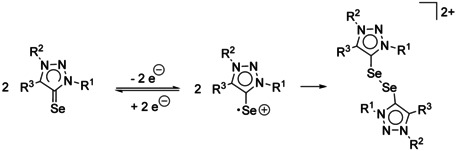
Proposed EC mechanism of the electrochemical oxidation of the selenium adduct with follow‐up dimerization.

The first oxidation is irreversible for all selenium adducts. The peak potential of the first oxidation at a scan rate of 0.1 V s^−1^ is found between −0.1 V and 0.4 V. The oxidation seems to proceed as an EC mechanism with a re‐reduction of the formed species, which is found at more negative potential than the expected re‐reduction of the oxidized selone. Similar observations have been previously made for NHC thiones and selones.^[7]^ Most likely, dimerization of the selenium adducts occurs (Scheme 1), which has been proposed^[8]^ and observed before for NHC sulfur adducts,^[9]^ and during electrochemical synthesis of disulfides.^[10]^ Recently, 1,2,3‐triazolin di‐ and tetraselinides have been isolated after chemical oxidation.^[11]^

**Figure 3:**
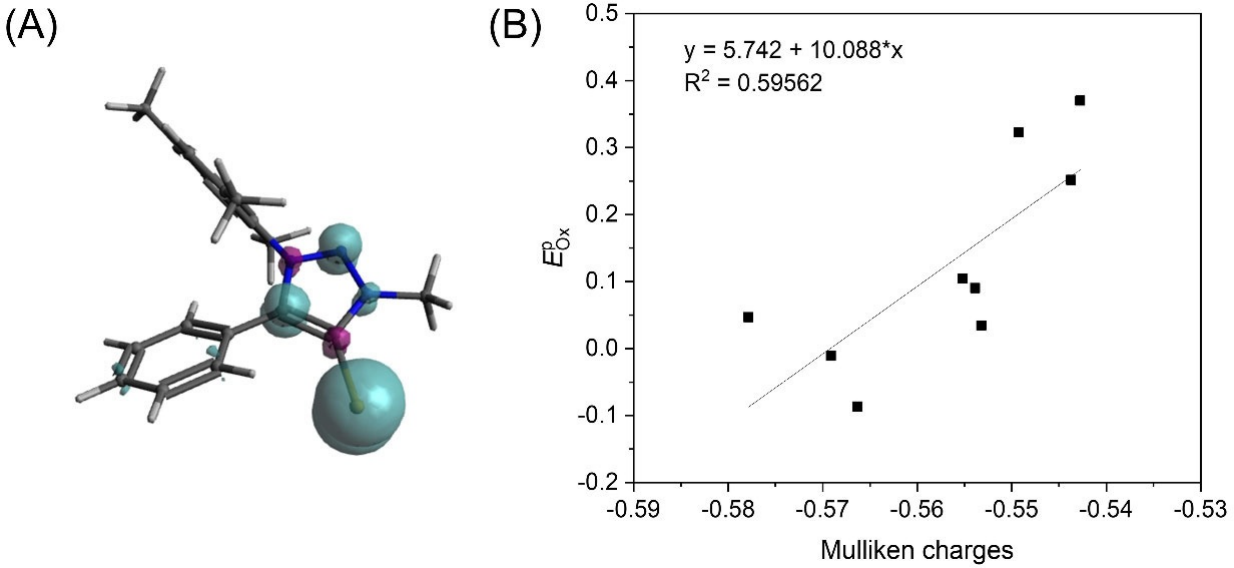
(A) Exemplary spin density plot of **[MesPh1,5TrzSe]^.+^** (isovalue=0.004). (B) Plot of *E* ^p^_ox_ against Mulliken charges and a goodness‐of‐fit *R* ^2^=0.60.

Even at high scan rates of up to 50 V s^−1^, no direct re‐reduction wave corresponding to the oxidation wave could be observed. DFT calculations confirm the assumption that the oxidation is mainly selenium centered with an exemplary spin population of 79.5 % on selenium calculated for the oxidized **MesPh1,5Trz** (Figure 3). Initially, we planned to analyze if the oxidation potential is linked to the overall donor properties of the triazolylidene to the selenium. Plotting of the peak potential of the first oxidation *E* ^p^_ox_ against DFT‐derived Mulliken charges at the selenium atoms, a more qualitative relation observed (*R* ^2^=0.60, Figure 3, Table S17 in the Supporting Information). The fact that the spin population of the oxidized radical does not fully lie on the selenium and the lack of reversibility probably blurs this correlation. Furthermore, the broad oxidation waves observed in the cyclic voltammograms of several compounds complicate the sharp definition of the peak potentials. In any case, it would be questionable, if the electronic properties of the selenium adduct could be quantitatively correlated to the properties of a triazolylidene in a transition‐metal complex. Overall, the analysis of the peak potential of the selenium adducts only led to a qualitative estimation of the overall donor properties of the MIC to the selenium.

Besides the oxidation wave, a reduction wave at a potential between −2.4 V and −2.7 V was observed in the CV of all the triazoline selones. The analysis of the reduction processes was carried out with similar concentrations of the substrates in acetonitrile (1.4–1.9 mmol L^−1^) and with exactly the same electrochemical set‐up to be able to compare the results quantitatively even though the various selones exhibit similar reduction properties. For **PhCF_3_PhTrzSe**, the first reduction is followed by a second reduction and it was not possible to separate the processes at 0.1 V s^−1^.

**Figure 4:**
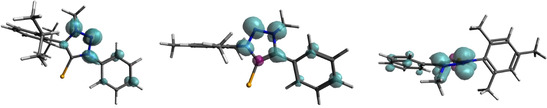
Exemplary spin density plot of **[PhDippTrzSe]^.^** ^−^ and **[MesPhTrzSe]^.−^** (isovalue=0.004).

DFT spin density calculations of the reduced species of the selenium adducts indicate that the first reduction, as intuitively expected, is centered on the triazolylidene moiety, mainly on the nitrogen atoms (Figure 4 and Table S15 in the Supporting Information). For **[PhDippTrzSe]^.^** ^−^, 74 % of the spin density is located on the triazolyline moiety itself and 0 % on selenium.

**Figure 5:**
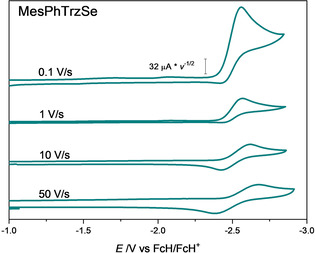
First reduction of **MesPhTrzSe** at various scan rates. The current was scaled by multiplying with (scan rate)^−1/2^ (in MeCN, electrolyte: NBu_4_PF_6_, electrode: glassy carbon, at a scan rate of 50 V s^−1^: *IR* drop compensation of 50 Ω).

**Scheme 2:**
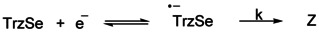
EC mechanism of the reduction of the triazolylidene selenium adducts.

An increase of the scan rate in cyclic voltammetry resulted in the appearance of a re‐oxidation wave for all selenium adducts. The shape of the reduction at different scan rates of **MesPhTrzSe** is depicted in Figure 5 (further in the Supporting Information, Figures S9–20). At a scan rate of 50 V s^−1^, the peak current ratio of the re‐oxidation wave to the reduction wave is relatively high. This is a strong indication that the reduction can be described with an EC mechanism (Scheme 2). By increasing the scan rate, the kinetically now inferior reaction to species Z does not take place. Analysis of the DFT‐derived geometries of the reduced species was performed to shed light on the decomposition process to species Z. A weakening of the bonds of the triazoline moieties is observed except for the carbene–C connecting bonds, which is expected as the negative charge is mainly located there. Furthermore, in the DFT‐optimized structures of the reduced species, the C^methyl^−N bond of reduced species is bent out of the triazoline plane to a large extent (Figure 4, Table S16 in the Supporting Information). This could be one of the destabilizing factors in the structure.

From the cyclic voltammograms at 50 V s^−1^, half‐wave potentials *E* ^1/2^ for the selenium adducts can be determined, which can be approximated as the formal potentials *E*°’_red_ of the adducts. The expected trend of a more negative *E* ^1/2^ with more electron‐donating substituents at the triazolylidene moiety is observed (Figure S20, Table S17 in the Supporting Information).

### Correlation of electrochemical data with DFT calculations: Computational screening of triazoline selones and triazolylidenes with specific reduction potentials

**Figure 6:**
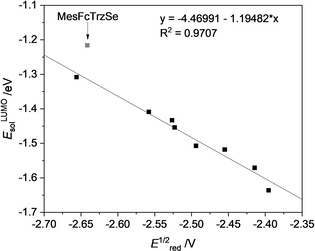
Correlation of *E*_sol_^LUMO^ and *E* ^1/2^_red_ (≅ *E*°’_red_) with a goodness‐of‐fit *R* ^2^=0.97. **MesFcTrzSe** was treated as an outlier and is not included in the correlation calculations.

Plotting the DFT‐derived LUMO energy levels of the molecules in acetonitrile *E*_sol_^LUMO^ against the formal potential *E*°’_red_, a strong correlation is observed (Figure 6). The ferrocenyl‐substituted selenium adduct seems to be an outlier here. This could be due to strong conformational effects, which are often pronounced for ferrocenyl substituents.

In the case of the investigated series of selenium adducts, the LUMO levels can be estimated from experimentally available formal potentials of the substances, and vice versa [Eqs. 1 and 2]. These equations are likely to be applicable as general equations for selenium adducts of monodentate triazolylidenes. The slope of −1.19 in Equation (1) points to the assumption that the specific energy term *E*_specific_ in our series correlates with the formal redox potential (more information describing the linkage of *E*_sol_^LUMO^ and *E*°’_red_ is given in the Supporting Information, Figure S30). This might be due to the structural similarity of the investigated molecules. Structurally similar molecules often show this correlation of properties, which are known to be associated with electron transfer, like changes in solvation energy.^[12]^

(1) $$E_{sol}^{LUMO\;\left(TrzSe\right)}=-\left(1.19\;\times {E{{}^{\circ }}^{'}}_{red}+4.47\right)$$

(2) $${E{{}^{\circ }}^{'}}_{red}=-\left(0.81\;\times \;E_{sol}^{LUMO\;\left(TrzSe\right)}+3.70\right)$$

The formula might find application in the design of selones with a specific reduction potential. Such selenium adducts are promising ligands for (transition) metal complexes.[^4c^, ^5^] With easily available calculated LUMO levels, promising candidates could be detected prior to time‐ and cost‐intensive laboratory synthesis and electrochemical analysis. The advantages of this approach over calculating the redox potential by quantum mechanical calculations directly^[13]^ are: it is less computationally intensive and requires less advanced calculations for a faster and more convenient screening of substances.

**Figure 7:**
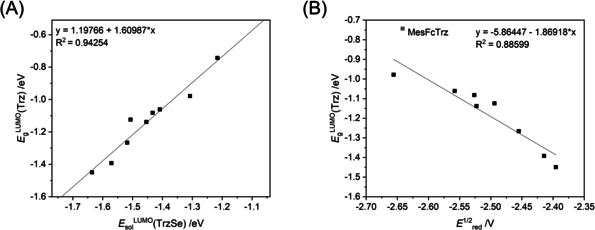
(A) Correlation of the LUMO energy level of the selenium adduct in acetonitrile *E*_sol_^LUMO^(TrzSe) with the LUMO energy level of the triazolylidene in gas‐phase *E*_g_^LUMO^(Trz). (B) Plotting the LUMO energy level of the triazolylidenes against the half‐wave potential of the first reduction of the selenium adducts. **MesFcTrzSe** was treated as an outlier and is not included in the correlation calculation.

**Figure 8:**
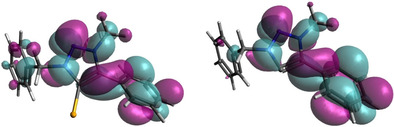
Illustration of the LUMO orbitals of BnPhTrzSe and BnPhTrz.

On comparing the LUMO levels of the selenium adducts in acetonitrile with calculated LUMO levels of the free triazolylidenes, a linear correlation with a goodness‐of‐fit of *R* ^2^=0.95 is observed (Figure 7). This fact can be explained with very similar orbital composition of the LUMO levels of the singlet triazolylidene‐based MICs and the corresponding selenium adducts (Figure 8). Taking this correlation into account, the determination of the formal reduction potential of the triazoline selones can be an indirect experimental method to not only approximate the LUMO level of the selone itself but also of the corresponding, challenging to isolate triazolylidenes (Figure 7). As the LUMO level of a ligand in the case of NHCs sometimes is related to their π‐accepting abilities,[^6b^, ^14^] we wanted to figure out if this is the case for triazolylidenes. Examination of the orbital compositions of the MICs displayed no significant population of the LUMO on the carbene carbon, so the direct relation between the LUMO energies of the triazolyidenes and their π‐accepting properties is questionable. The LUMO level might still indicate the trend of how prone the triazolylidene is for uptake of electron density in a coordinated situation.

For the determination of the selenium adducts’ half‐wave potential, very high scan rates must be applied. This can sometimes be an instrumental challenge. We wanted to know if also the peak potential of the reduction at common scan rates can provide insight into LUMO levels of the selones and triazolylidenes. In our investigated series of selones, the peak potentials *E* ^p^_red_ show correlation with the half‐wave potentials with *R* ^2^=0.90 (Figure S31 in the Supporting Information). It seems to be possible to take *E* ^p^_red_ as a measure for this series of molecules. Nevertheless, the correlation of *E* ^p^_red_ to the LUMO energy levels is less accurate.

### UV/Vis/NIR and EPR spectroelectrochemical investigations and TD‐DFT calculations on a triazoline selone: (Quasi‐)reversible electrochemical behavior of triazoline selones displaying a specific reduction potential

Remarkably, **PhDippTrzSe** and **MesPh1,5TrzSe** show a re‐oxidation wave with a high peak current ratio to the reduction wave in cyclic voltammetric investigations at 0.1 V s^−1^ (Figure 2). **PhDippTrzSe** (peak current ratio of 0.89 and a peak‐to‐peak separation of 75 mV) and **MesPh1,5TrzSe** (peak current ratio 0.83, peak‐to‐peak separation of 81 mV) display at least quasi‐reversibility. The relatively high peak‐to‐peak separations might also be due to high reorganization energies upon reduction (e.g., bending of C^methyl^−N bond as described before). The two selenium adducts seem to meet a “reversiblity range”/“sweet spot” as their half‐wave potentials *E* ^1/2^=−2.46 V (**PhDippTrzSe**) and *E* ^*1*/2^=−2.49 V (**MesPh1,5TrzSe**) are very similar. The first sign of a re‐oxidation wave for **PhPhTrzSe** with a similar reduction potential supports this assumption. Triazoline selones that show reduction potentials in the described range seem to stabilize the radical anion better than other triazoline selones. However, this observation is purely empirical and would require further work for a sound understanding. Through Equation (2), which was proposed before, further triazoline selones with a reduction potential in the range of −2.46 to −2.49 V might be detected in theoretical calculations and the existence or coincidence of the “reversibility range” could be investigated. As for **[PhDippTrzSe]^.^** ^−^ and **[MesPh1,5TrzSe]^.^** ^−^ in DFT geometry optimization calculations, the bending of the methyl group out of the triazoline plane is observed to be less distinct (Table S16 in the Supporting Information) and the hypothesis, that this bending is a destabilizing factor, is supported.

**Figure 9:**
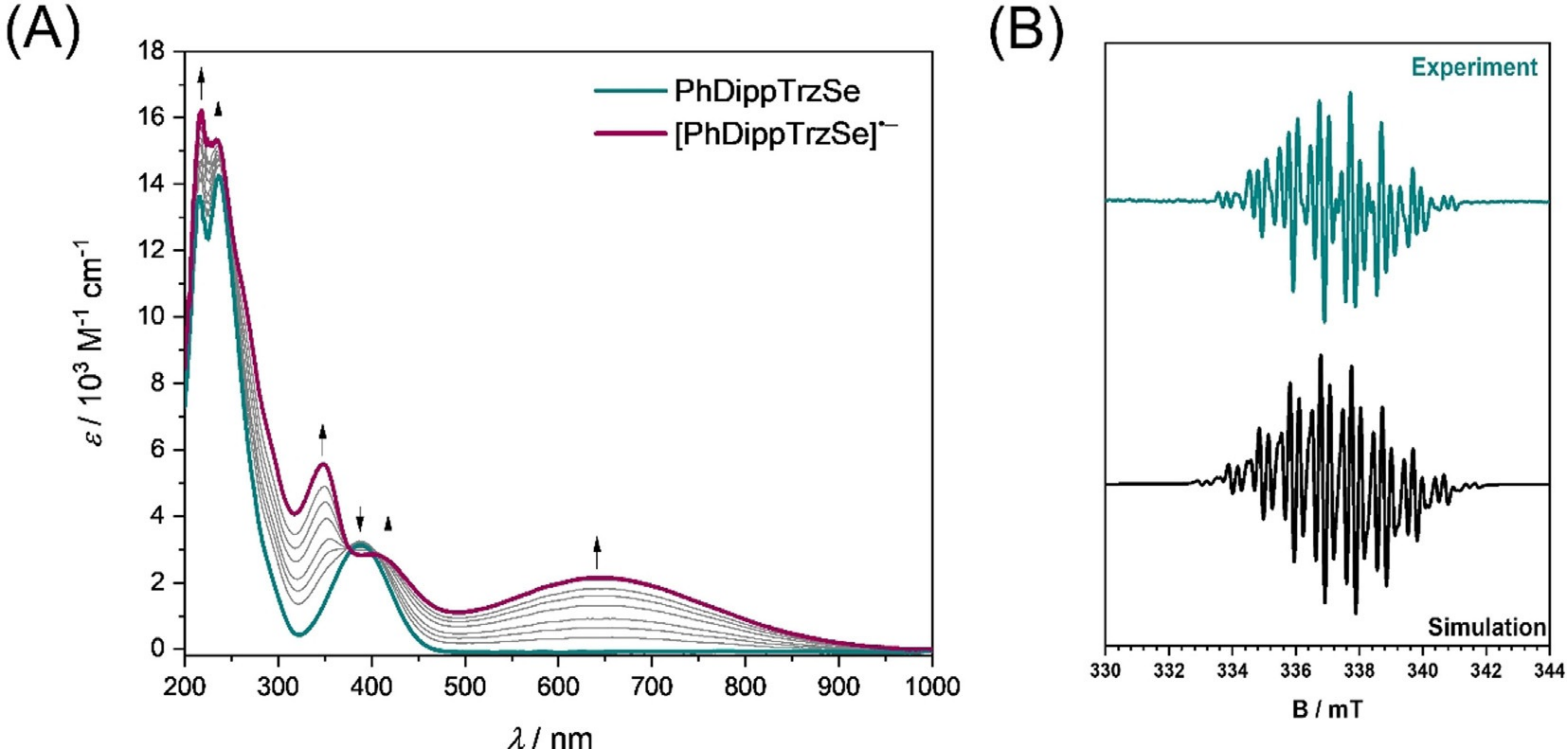
(A) UV/Vis/NIR spectroelectrochemistry of **PhDippTrzSe** to **[PhDippTrzSe]^.−^** measured in acetonitrile/NBu_4_PF_6_ with a gold electrode. (B) Experimental EPR spectrum of the in situ electrochemically generated **[PhDippTrzSe]^.−^** in MeCN/NBu_4_PF_6_ with a platinum electrode and the simulated spectrum.

The electrochemical reduction of **PhDippTrzSe** was analyzed extensively by a combination of UV/Vis/NIR and EPR spectroelectrochemical (SEC) methods and time‐dependent DFT (TD‐DFT) calculations. Investigation through UV/Vis/NIR spectroelectrochemistry was performed in acetonitrile in an optically transparent thin‐layer electrochemical (OTTLE) cell. In the initial spectrum of **PhDippTrzSe** (Figure 9), the bands at 218, 236, and 387 nm can be assigned to transitions from the electron‐rich selenium to the π‐orbitals of the aromatic substituents and the triazolylidene moiety (for TD‐DFT calculations, see Tables S1–5, Figures S27 and S28 in the Supporting Information). Upon reduction, new bands at 351, 410, and 645 nm are observed. These bands in general display a charge transfer character from the triazolylidene ring to the adjacent π‐systems. The broad band in the range of visible light (645 nm) is often typical for organic radicals. This result points further to a triazolylidene‐centered reduction. After re‐oxidation, the initial spectrum is mainly recovered (about 90 %; Figure S24 in the Supporting Information). Longer electrolysis at reductive potentials led to a smaller recovery (about 80 %) of the native spectrum after complete reduction and re‐oxidation cycles during the spectroelectrochemical measurements (Figure S25 in the Supporting Information). Over a small timescale, the reduction displays reversible behavior but overall partial chemical reversibility of the process is observed.

EPR spectroscopy of the in situ electrochemically generated species **[PhDippTrzSe]^.^** ^−^ shows a signal with a *g*‐value of 2.003 and hyperfine coupling to several nuclei (Figure 9). This spectrum could be simulated with sufficient accuracy by considering the interaction of the unpaired electron with three ^14^N and four ^1^H nuclei (Table S8 in the Supporting Information). These results are an additional indication for the localization of the anionic radical on the triazolylidene moiety.

A reversible reduction on the MIC moiety is extremely rare. A heteroleptic triazolylidene gold complex and a cationic homoleptic triazolylidene gold complex showed low chemical reversibility of the reduction at −40 °C, which was detected through a proportionally small re‐oxidation wave.^[15]^ Quasi‐reversible or reversible reduction have been observed in chelating ligands containing triazolylidenes (for example, with additional pyridyl substituents).^[16]^ However, in those cases, the radicals are often stabilized through the help of the second ligand that is part of the chelating system. The above results are on a mono‐dentate triazolylidene, which makes the reversible reduction unique.

Amongst NHCs, the ability to stabilize radicals or show reversible reductions on the ligand is a typical property of cyclic alkyl(amino) carbenes (CAACs).[^2f^, ^3a^, ^3b^, ^3c^, ^3d^, ^17^] In these cases, the spin density of the reduced species is mainly located on the carbene carbon. This is a significant difference to the triazolylidenes investigated here, with the spin density of the reduced species mainly located on the nitrogen atoms of the triazolylidene moiety. For the CAACs, the small HOMO–LUMO gap is believed to be the cause of their radical stabilization abilities. With 4.25 eV to 4.47 eV, the HOMO–LUMO gap for the herein investigated triazolylidenes is also comparably small, but no hint for a higher reversibility within the series for a smaller HOMO–LUMO gap or lower lying LUMO energy level could be found.

### Synthesis and characterization of gold(I) chloride complexes: First attempt to transfer the radical anion stabilizing property from a triazoline selone to a metal complex

**Scheme 3:**
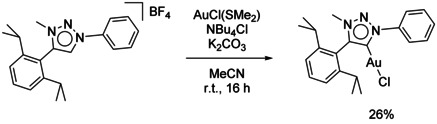
Synthesis of **(PhDippTrz)AuCl**.

To investigate the synthetic potential of **PhDippTrz** and to explore if the reversibility of reduction also remains when incorporating these triazolylidenes in transition‐metal complexes, a gold chloride complex with the **PhDippTrz** ligand was synthesized (Scheme 3). Gold triazolylidene complexes have recently found use in homogeneous catalysis, in photochemistry, and they have also been investigated for their biological activity.[^15^, ^18^] The synthetic strategy was deprotonation of the corresponding triazolium salt and coordination through a weak base route, introduced by Nolan and co‐workers for normal NHCs.^[19]^ Potassium carbonate was added as the base and the reaction could be performed under ambient conditions at room temperature. Usually, to generate NHC gold chloride complexes that way, azolium chlorides have been used and it was shown that a dihalide aurate is an intermediate in this reaction. As the synthesis of triazolium chlorides is challenging, the triazolium tetrafluoroborate was deployed as the ligand precursor. To achieve formation of the aurate, tetrabutylammonium chloride was added as a chloride source. Work‐up through fast filtration over silica yielded **(PhDippTrz)AuCl** with a yield of 26 %. The low yield might be due to decomposition reactions as a dark‐purple precipitate is formed during the reaction and work‐up. The absence of the signal corresponding to the triazolium proton in the ^1^H NMR spectrum points to the successful synthesis of the target molecule. Furthermore, the compound was characterized through ^13^C NMR spectroscopy and ESI mass spectrometry (Figures S1 and S2 in the Supporting Information).

**Figure 10:**
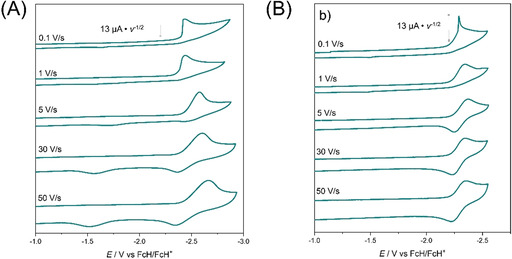
First reduction of **(PhDippTrz)AuCl** at various scan rates. The current was scaled by multiplying with (scan rate)^−1/2^. The cyclic voltammograms were measured in DMF with (A) NBu_4_PF_6_ and (B) NBu_4_Cl as the electrolyte and a glassy carbon working electrode. The sharp peak of the curve at 0.1 V s^−1^ cannot be explained yet. No adsorption at the electrode was observed upon optical examination after measurement.

**Scheme 4:**
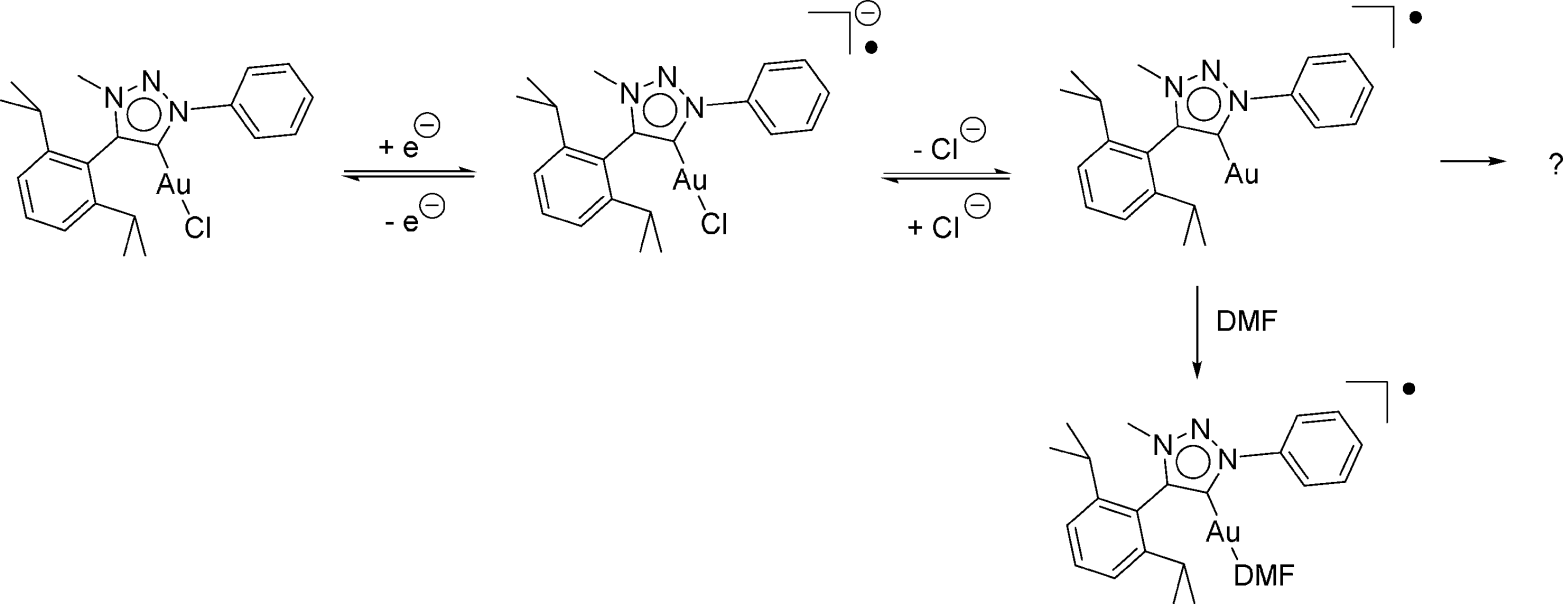
Proposed EC mechanism of the reduction of **(PhDippTrz)AuCl** with follow‐up chloride abstraction and solvent coordination.

The cyclic voltammograms of **(PhDippTrz)AuCl** at 0.1 V s^−1^ displayed an irreversible reduction process at *E* ^p^_red_=−2.43 V (Figure 10 A) and no oxidation process. At first sight, this points to a restricted transferability of the radical anion stabilizing properties of the ligand from the selenium adduct to a gold complex. Measurements at higher scan rates up to 50 V s^−1^ led to the appearance of a re‐oxidation wave and a second re‐oxidation wave shifted to more positive potentials (*E* ^p^_re‐ox1_=−2.34 V; *E* ^p^_re‐ox2_=−1.51 V). Similar observations have been made with MIC rhenium CO chloride complexes in previous studies.^[16a]^ In those cases, the shape of the cyclic voltammogram was attributed to chloride abstraction and further coordination of DMF to the radical rhenium species. To investigate if chloride dissociation is also the follow‐up reaction after reduction of **(PhDippTrz)AuCl**, measurements with a chloride‐containing supporting electrolyte (NBu_4_Cl) were performed (Figure 10 B). Here, the re‐oxidation wave already appeared at lower scan rates and no second re‐oxidation wave was observed. This is a strong indication for an EC mechanism taking place with chloride abstraction as the follow‐up reaction. So, overall chloride abstraction is proposed upon reduction with subsequent partial coordination of DMF at high scan rates to the formed radical gold species (Scheme 4). The cleavage of the Au−Cl bond is an important elementary step in Au^I^‐catalyzed reactions.^[20]^ Our observations show that it should be possible to cleave that bond through application of a redox potential when using gold complexes in electrocatalysis.

### Synthesis and cyclic voltammetry of gold(I) phenyl complexes: Revised attempt to transfer the radical anion stabilizing property from a triazoline selone to a metal complex

**Scheme 5:**
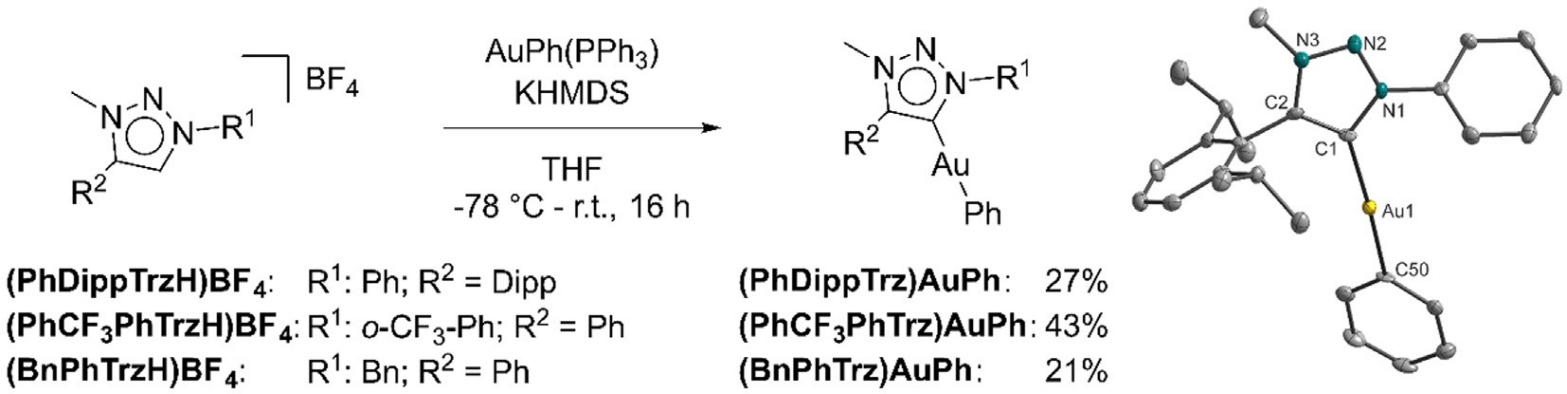
Synthesis of the phenyl triazolyidene gold complexes and molecular structure of **(PhDippTrz)AuPh** in the crystal. Thermal ellipsoids are drawn at the 50 % probability level. Hydrogen atoms are omitted for clarity.

**Figure 11:**
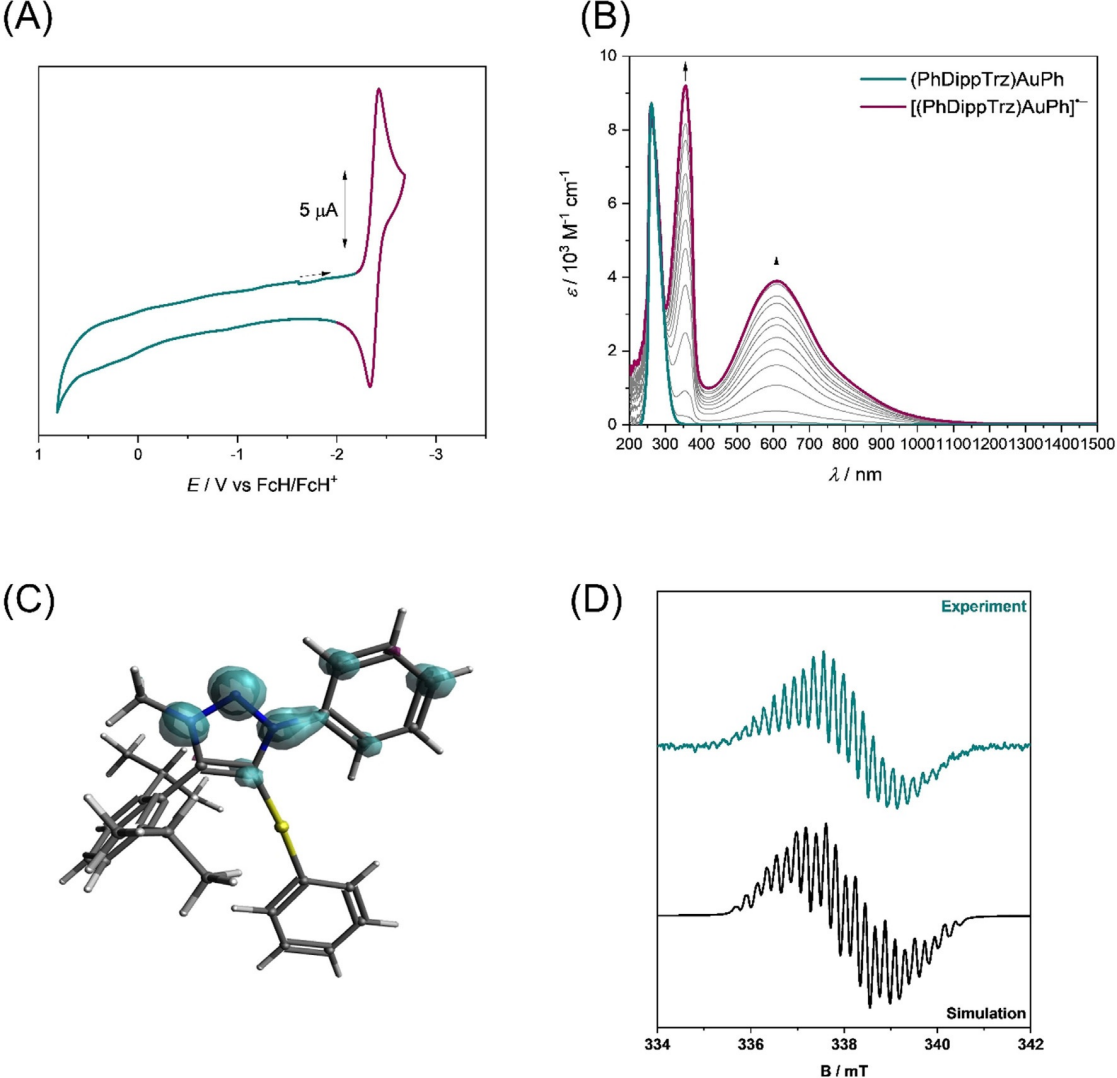
(A) Cyclic voltammogram of **(PhDippTrz)AuPh** in DMF at 0.1 V s^−1^ with NBu_4_PF_6_ as electrolyte and a glassy carbon working electrode. The colors indicate the potential ranges in which the neutral (cyan) and reduced (magenta) species are observed in the SEC experiment. (B) UV/Vis/NIR spectroelectrochemistry of **(PhDippTrz)AuPh** to **[(PhDippTrz)AuPh]^.−^** measured in DMF/NBu_4_PF_6_ with a gold electrode. (C) Spin population plot of **[(PhDippTrz)AuPh]^.−^** (isovalue=0.004). (D) Experimental EPR spectrum of the in situ electrochemical generated **[(PhDippTrz)AuPh]^.−^** in DMF/NBu_4_PF_6_ with a platinum electrode and the simulated spectrum.

To avoid this chloride abstraction mechanism (see above), a further MIC gold complex was synthesized with phenyl as the second ligand instead of chloride. Following a synthetic route introduced by Bertrand, Bezuidenhout, and co‐workers,[^18a^, ^18i^] the gold complex **(PhDippTrz)AuPh** was synthesized (Scheme 5). Recrystallizing in CH_2_Cl_2_/hexane resulted in the complex with 27 % yield. The absence of the signal corresponding to the triazolium proton in the ^1^H NMR spectrum points to the successful synthesis of the target molecules. **(PhDippTrz)AuPh** was characterized through ^13^C NMR spectroscopy and ESI mass spectrometry (Figures S3 and S4 in the Supporting Information). The purity of the complex was confirmed through elemental analysis. Furthermore, single crystals were grown in CH_2_Cl_2_ layered with hexane, and analyzed by single‐crystal X‐ray diffraction (XRD; Scheme 5). The C‐Au‐C angle (173.3°) is slightly bent from 180°. Cyclic voltammetric investigations indeed displayed a reversible reduction process at 0.1 V s^−1^ (Figure 11 A) and at lower scan rates measured down to 0.025 V s^−1^ (Figure S20 in the Supporting Information) with a half‐wave potential of *E* ^1/2^_red_=−2.38 V. To investigate if there was really a transfer of the reduction properties from the selenium adduct to the gold complex, two further complexes, **(PhCF_3_PhTrz)AuPh** and **(BnPhTrz)AuPh**, were synthesized (Scheme 5) and characterized through ^1^H, ^13^C NMR spectroscopy, ESI mass spectrometry (Figures S5–8 in the Supporting Information). The purity was confirmed through elemental analysis. Cyclic voltammetric investigations displayed a fully irreversible reduction process as expected from the results of the triazoline selones (Figures S22 and S23 in the Supporting Information).

### UV/Vis/NIR and EPR spectroelectrochemical investigations and DFT calculations on a gold phenyl complex: Detailed analysis of the metal‐bound MIC radical

The reduction of **(PhDippTrz)AuPh** was analyzed extensively through a combination of UV/Vis/NIR and EPR spectroelectrochemical methods and DFT calculations.

UV/Vis/NIR spectroscopic investigations of the gold complex showed a single band in the UV region (*λ*_max_=263 nm) for the native species in DMF (Figure 11 B). Upon reduction, two new bands in the near UV and visible spectral region arise (*λ*_max_=355 nm, *λ*_max_=611 nm). After running a complete redox‐cycle, the spectrum of the native species was regenerated quantitatively (Figure S26 in the Supporting Information), proving the reversible nature of the electrochemical reduction. This spectroelectrochemical behavior is comparable to the first reduction of CAAC‐stabilized organic anionic radicals observed by Jana and our group.^[21]^

DFT spin density calculations of the reduced species of the gold complex indicate that the first reduction is centered on the triazolylidene moiety as this was shown for the corresponding selenium adduct (Figure 11 C). The spin density for **[(PhDippTrz)AuPh]^.^** ^−^ also appears to be located mainly on the triazolylidene moiety and its aromatic substituents (71 % on the triazolylidene ring). Interestingly, the calculations predict the spin density is located on the carbene carbon at 5 % probability. The observed hyperfine coupling constant (HFCC) values in the EPR spectra of **[(PhDippTrz)AuPh]^.^** ^−^ and **[PhDippTrzSe]^.^** ^−^ support the calculated spin density distribution. They display the resulting lower spin density at the nitrogen atoms of the ring in the reduced gold complex compared with the reduced triazoline selone. The radical anionic gold species can be placed between the reduced selenium adduct with no spin density at the carbene carbon and CAACs with the spin density mainly located on the carbene carbon.

EPR spectroscopy of the electrochemically in situ generated species **[(PhDippTrz)AuPh]^.^** ^−^ shows a signal with a *g*‐value of 2.004 and hyperfine couplings to several heteroatoms (Figure 11 D). This spectrum was simulated with good accuracy with the signal arising from the interaction of the unpaired electron with three ^14^N and six ^1^H nuclei (Table S9 in the Supporting Information).

To the best of our knowledge, this is the first time a reversible reduction on a monodentate MIC metal complex at room temperature has been observed and the corresponding radical anion unambiguously characterized.

### Chemical reduction of a gold phenyl complex in bulk: First analysis of the stability of the reduced species

**Figure 12:**
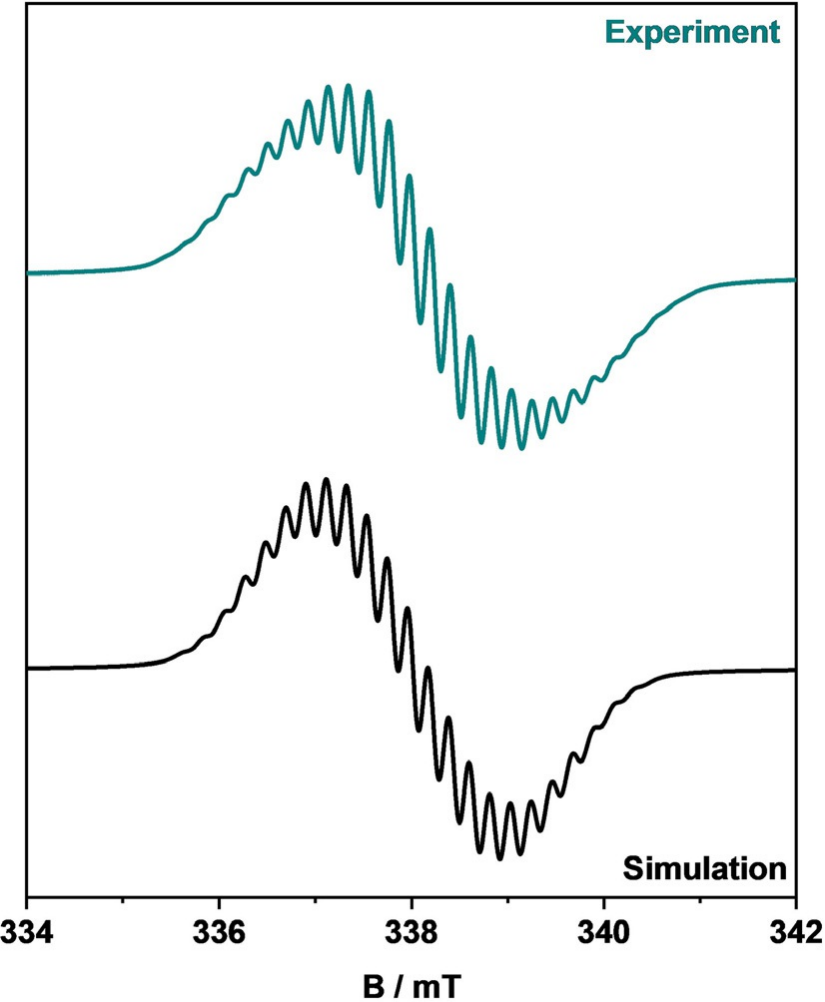
Experimental EPR spectrum of the in situ in bulk generated **[(PhDippTrz)AuPh]^.−^** through chemical reduction and the simulated spectrum at −40 °C in THF.

EPR spectroelectrochemistry does not usually afford a quantitative conversion and only displays the species generated on the electrode. As we wanted to estimate the stability of the generated radical, we decided to generate the radical anionic **[(PhDippTrz)AuPh]^.^** ^−^ through chemical reduction. When potassium or potassium graphite was added to a colorless solution of **(PhDippTrz)AuPh** in THF in a Schlenk tube at −78 °C under inert conditions, the formation of a blue species was observed. This fits to the expected color of the reduced species, which displayed a band with a maximum at 611 nm in the UV/Vis spectroelectrochemical investigations discussed before. Attempts to transfer the blue solution with Schlenk techniques under careful exclusion of air to analyze the in situ generated species through EPR spectroscopy were not successful. The color of the solution changed immediately from blue to yellow. The EPR spectrum only displayed a (singlet) signal of an unidentified species, which probably belongs to a decomposition product of the complex. Therefore, we decided to perform in situ reduction in the EPR tube. To **(PhDippTrz)AuPh** in an EPR tube, an excess of potassium graphite was added in a quartz capillary. The EPR spectrum of the solids displayed a very strong singlet signal, which we assigned to potassium graphite. At −40 °C, dry freshly degassed THF was added, which led to the immediate appearance of a new signal with a rich hyperfine coupling pattern. The hyperfine pattern became better resolved over 20 min, presumably owing to an increase of the **[(PhDippTrz)AuPh]^.^** ^−^ signal as the reaction went forward. After about 20 min, a well‐resolved signal with hyperfine coupling and a *g*‐value of 2.0023 was recorded with substantially higher intensity and better signal‐to‐noise ratio compared with the signal from the electrochemically generated species (Figure 12). The spectrum of the chemically reduced species could be simulated with good accuracy with the signal arising from the interaction of the unpaired electron with three ^14^N nuclei and six ^1^H nuclei, divided in groups of 3, 2, and 1 equivalent nuclei (Table S10 in the Supporting Information). The spectra of the electrochemically and chemically generated species are quite similar, and display compatible hyperfine patterns, most notably the splittings from the three ^14^N nuclei in the triazolylidene. However, the chemically generated species displays a much better resolution, which allowed a more accurate simulation of the small ^1^H hyperfine splittings. From the similar hyperfine patterns, we conclude that both signals arise from the same species, and variations arise from the different conditions (solvent, temperature, concentration). DFT calculations suggest (see above) that the methyl protons and the *ortho*‐ and *para*‐phenyl protons have appreciable spin density and could explain the experimental hyperfine splittings. After recording the EPR spectra at −40 °C, we increased the temperature to 0 °C, expecting to observe a slow decay of the signal. However, on the first measurement, we only observed an unresolved singlet signal, which again is most likely attributed to KC_8_/K or to a decomposition product of the gold complex. This indicates that the **[(PhDippTrz)AuPh]^.^** ^−^ species likely decomposes over a period of time on increasing the temperature. It should be mentioned here that the set‐up used by us for performing the EPR analyses would allow the diffusion of air into the EPR tube over a period of time. Thus, the question as to whether the decomposition of the radical is temperature‐driven or due to contact with air still remains open. The solution in the EPR tube appeared to be red/yellowish with a dark precipitate after removing it from the spectrometer. Overall, the reduced species **[(PhDippTrz)AuPh]^.−^** was formed successfully in bulk through chemical reduction at lower temperatures.

As for the MIC selenium adducts, it would be interesting to identify the formed decomposition product in future research. This might help in the process of avoiding the decomposition and stabilizing the reduced species. In general, it has to be noted, that **[(PhDippTrz)AuPh]^.−^** is a very strong reducing agent and highly challenging to handle.

## Conclusion

Extensive studies on the electrochemical behavior of triazolylidene selenium adducts and triazolylidene gold complexes have been performed.

The selenium‐centered oxidation observed in cyclic voltammetry is not reversible and not fully selenium centered. Plotting the peak potentials against the DFT‐derived Mulliken charges exhibits an only qualitative correlation of the oxidation potential to the net donor strength of the triazolylidenes in the adduct.

The first reduction of all investigated triazoline selones displays a re‐oxidation wave at high scan rates. This enables the determination of the formal potential of the reduction process. This potential shows excellent correlation to the LUMO levels of the triazoline selones and correlation to the LUMO levels of the corresponding triazolylidenes. Easily performed prediction of the redox behavior through calculation of the LUMO levels of triazoline selones appears to be possible.

The triazolylidene‐centered reduction investigated through cyclic voltammetry at low scan rates surprisingly displayed quasi‐reversible behavior for **PhDippTrzSe** and **MesPh1,5TrzSe**. These two selones exhibit very similar reduction potentials, which led to the proposal of a “reversibility range”. Further detailed investigations of **PhDippTrzSe** showed that this reduction is mainly centered on the nitrogen atoms of the triazolylidene moiety.

Additionally, the transferability of the radical anion stabilizing property from the MIC selenium adduct to the corresponding MIC gold complex has been shown. Consequently, the easily available selenium adducts are excellent probes for triazolylidene ligands in radical anion stabilizing complexes. The synthesis of the complexes is usually synthetically more demanding and pricier. The derived gold complex was reduced chemically in bulk and analyzed through EPR spectroscopy.

**Figure 13:**
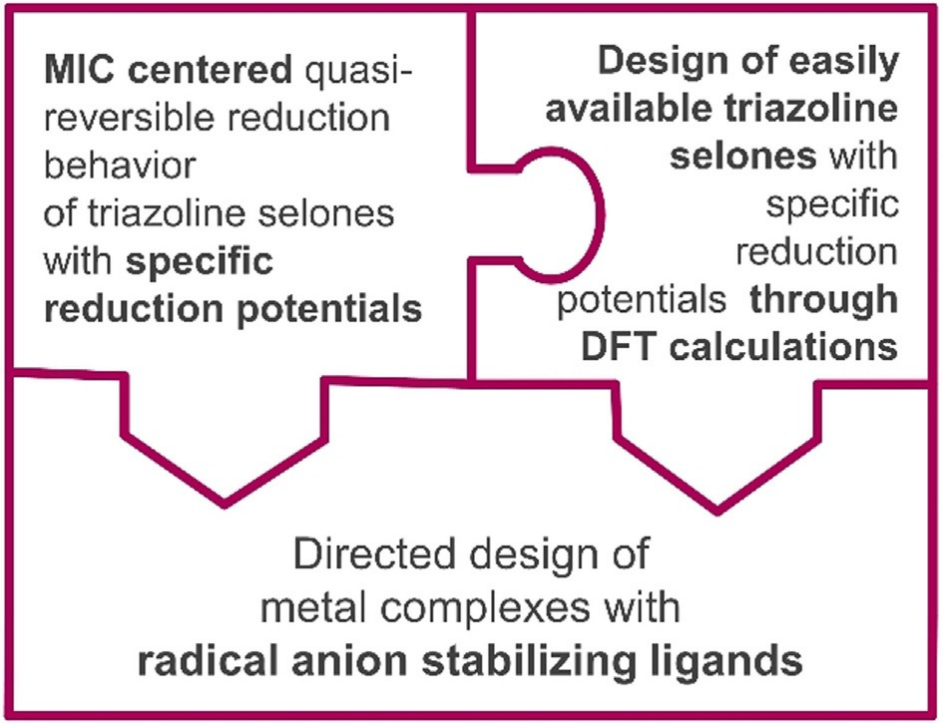
Summary of results.

Overall, the electrochemical investigation of the triazolylidene selenium adducts gives only qualitative insight into the electron‐donating and ‐accepting abilities of the triazolylidene MICs. Instead, these investigations pave the way to the directed design of redox active metal complexes with triazolylidenes as ligands through pre‐synthetic DFT calculation (Figure 13). In the future, through inexpensive, fast, and easy computational screening, further triazolylidenes with corresponding selenium adducts having the reduction potential in the reversibility range could be discovered. Through easy synthesis of the selones, the hypothesis of the “reversibility range” could be verified. The corresponding MICs might be promising candidates to stabilize radical anions similar to CAACs. Furthermore, it would be of high interest to compare the performance of triazolylidenes versus CAACs in this field and to investigate how the different spin density location of the unpaired electron influences the properties of these different radical classes. Concluding, we have presented here the first example and complete spectroscopic analysis of a monodentate triazolylidene‐based MIC radical anion. Additionally, we have presented guidelines for predicting the stability of MIC‐based anion radicals.

## Experimental Section

### Crystallographic data

Deposition number 2052185 contains the supplementary crystallographic data for this paper. These data are provided free of charge by the joint Cambridge Crystallographic Data Centre and Fachinformationszentrum Karlsruhe Access Structures service.

## Conflict of interest

The authors declare no conflict of interest.

## Supporting information

As a service to our authors and readers, this journal provides supporting information supplied by the authors. Such materials are peer reviewed and may be re‐organized for online delivery, but are not copy‐edited or typeset. Technical support issues arising from supporting information (other than missing files) should be addressed to the authors.

Supplementary
