# Supplementary material for: Directed Design of a AuI Complex with a Reduced Mesoionic Carbene Radical Ligand: Insights from 1,2,3‐Triazolylidene Selenium Adducts and Extensive Electrochemical Investigations
Source: Chemistry. 2021 Mar 17;27(21):6557–68. doi: 10.1002/chem.202100105 (PMC8252451; doi:10.1002/chem.202100105)
Supplement: Supplementary file 1 — Supplementary [file CHEM-27-6557-s001.pdf]

Chemistry–A European Journal

Supporting Information

Directed Design of a Au^I Complex with a Reduced Mesoionic Carbene Radical Ligand: Insights from 1,2,3-Triazolylidene Selenium Adducts and Extensive Electrochemical Investigations

Julia Beerhues,[a, b] Maren Neubrand,[a] Sebastian Sobottka,[b] Nicolás I. Neuman,[a] Hannes Aberhan,[b] Shubhadeep Chandra,[a] and Biprajit Sarkar*[a, b]

Supporting Information

Table of Contents

1 Synthesis and Characterization 3
2 NMR Spectra 5
3 Cyclic Voltammetry 9
4 UV/Vis/NIR Spectroscopy and Spectroelectrochemistry 19
4.1 Supplementary Spectra 19
4.2 TD-DFT Simulation 21
4.2.1 DFT Calculation for **PhDippTrzSe** (singlet state) 21
4.2.2 DFT Calculation for **PhDippTrzSe_1red** (doublet state) 22
5 Electron Paramagnetic Resonance (EPR) 25
5.1 General Remarks 25
5.2 Simulation 25
6 Single-Crystal X-Ray Diffraction Analysis 26
7 Correlations 28
8 DFT 31
9 Table of Data 45
10 References 46

1 Synthesis and Characterization

Schlenk-line techniques were performed under an inert atmosphere of argon (Air liquide, ALPHAGAZ™ 1, purity ≥99.999%). Commercially available chemicals were used without further purification. Dry solvents were available from GS GLOVEBOX solvent system or Innovative Technology PURESOLV solvent system. The selenium adducts were synthesized according to previous published methods.[1] The triazolium salts are literature known.[1,2] All solvents were degassed by standard techniques prior to use. ^{1}H NMR and $^{13}C\{^{1}H\}$ NMR spectra were recorded on a Bruker Avance III HD 700 NMR Spectrometer at 25 °C. Chemical shifts are reported in ppm (relative to the TMS signal) with reference to the residual solvent peaks.[3] Multiplets are reported as follows: singlet (s), duplet (d), triplet (t), quartet (q), quintet (quint), septet (sept), and combinations thereof.

Mass spectrometry was performed on a microTOFQ Bruker Daltonics. Elemental analysis was performed on a Elementar VarioMICRO cube.

(PhDippTrz)AuCl

In a glass vial under air atmosphere **(PhDippTrz)BF$_4$** (1.00 equiv, 10 mg, 0.025 mmol), K_2CO_3 (4.00 equiv, 13.5 mg, 0.098 mmol) NBu_4Cl (1.00 equiv, 7 mg, 0.025 mmol) and $[Au(SMe_2)Cl]$ (1.00 equiv, 7.4 mg, 0.025 mmol) were stirred in acetonitrile at 43 °C for 27 h. The solvent was removed under reduced pressure. The crude product was solved in CH_2Cl_2 and filtered over Silica. After removal of the solvent under reduced pressure colourless **(PhDippTrz)AuCl** (26%, 0.0065 mmol, 3.5 mg) was obtained.

^{1}H NMR (700 MHz, $CDCl_3$) δ (ppm) 7.86-7.85 (m, 2H, aryl H), 7.70-7.67 (m, 4H, aryl H), 7.42-7.41 (m, 2H, aryl H)f, 3.95 (s, 3H, N-CH_3), 2.34 (sept, 2H, *i*Pr-CH, 1.42 (d, 7 Hz, 6H, *i*Pr-CH_3), 1.19 (d, 7 Hz, 6H, *i*Pr-CH_3).

$^{13}C\{^{1}H\}$ NMR (176 MHz, $CDCl_3$) δ (ppm) 149.3, 143.0, 139.8, 135.6, 133.2, 132.0, 129.9, 126.0, 124.9, 120.6, 38.3, 32.1, 25.2, 24.5.

MS (ESI): *m/z* $[M\text{-}Cl]^+$ found 516.1704 calc. 516.1709.

(PhDippTrz)AuPh

In an oven dried Schlenk flask under argon **(PhDippTrz)BF$_4$** (1.00 equiv, 26.6 mg, 0.065 mmol) was dissolved in THF (2 mL) and cooled to -78 °C. Potassium hexamethyldisilazide (1.05 equiv, 13.6 mg, 0.068 mmol) was added. After 10 min of stirring a precooled solution of $(PPh_3)AuPh$ (1.00 equiv, 35 mg, 0.065 mmol) in THF (1 mL) was added. The reaction mixture was stirred under slow warming to room temperature for 16 h. The solvent was removed in vacuum and the crude product was washed with hexane (3 x 3 mL). After extraction with toluene (2 x 4 mL), filtration over celite and removing the solvent in vacuum the gold complex with minor impurities. Crystallization from a nearly saturated solution of the complex in CH_2Cl_2 layered with hexane yielded crystalline, colourless **(PhDippTrz)AuPh** (47%, 0.030 mmol, 18 mg).

^{1}H NMR (700 MHz, $CDCl_3$) δ (ppm) 8.50-8.49 (m, 2H, aryl H), 7.57-7.50 (m, 4H, aryl H), 7.35-7.34 (m, 2H, aryl H), 7.30-7.29 (m, 2H, aryl H), 7.11 (t, 2H, aryl H), 6.92 (t, 2H, aryl H), 3.86 (s, 3H, N-CH3), 2.54 (sept, 2 H, *i*Pr-CH), 1.36-1.35 (d, 7Hz, 6H, *i*Pr-CH_3) 1.20-1.19 (d, 7Hz, 6H, *i*Pr-CH_3).

$^{13}C\{^1H\}$ NMR (176 MHz, $CDCl_3$) δ (ppm) 180.6, 169.4, 149.3, 146.8, 140.8, 139.6, 131.4, 130.0, 129.3, 127.1, 124.5, 123.7, 123.7, 123.6, 36.8, 31.4, 25.3, 23.6.

MS (ESI): *m/z* $[M+H]^+$ found 594.2167 calc. 594.2178.

EA: found C 54.58, H 5.114, N 7.07 calc. C 54.64, H 5.09 N 7.08.

($PhCF_3PhTrz$)AuPh

In an oven dried Schlenk flask under argon **($PhCF_3PhTrz$)BF_4** (1.00 equiv, 36.5 mg, 0.093 mmol) was dissolved in THF (2 mL) and was cooled to -78 °C. Potassium hexamethyldisilazide (1.05 equiv, 19.5 mg, 0.098 mmol) was added. After 10 min of stirring a precooled solution of (PPh_3)AuPh (1.00 equiv, 50 mg, 0.093 mmol) in THF (2 mL) was added. The reaction mixture was stirred under slow warm-up to room temperature for 16 h. The solvent was removed in vacuum and the crude product was washed with hexane (3 x 3 mL). After extraction with toluene (2 x 4 mL), filtration over celite and removing the solvent in vacuum the gold complex with minor impurities. Crystallization from a nearly saturated solution of the complex in CH_2Cl_2 layered with hexane yielded crystalline, colourless **($PhCF_3PhTrz$)AuPh** (43%, 0.040 mmol, 23 mg).

^1H NMR (700 MHz, $CDCl_3$) δ (ppm) 8.05-8.04 (m, 1H, aryl H), 7.89-7.88 (m, 3H, aryl H), 7.80 (t, 1H, aryl H), 7.74 (t, 1H, aryl H) , 7.57-7.54 (m, 3H, aryl H), 7.30-7.29 (m, 2H, aryl H), 7.10 (t, 2H, aryl H), 6.91 (t, 1H, aryl H), 4.24 (s, 3H, N-CH_3).

$^{13}C\{^1H\}$ NMR (176 MHz, $CDCl_3$) δ (ppm) 183.1, 169.2, 147.9, 140.6, 137.2, 132.9, 131.0, 130.3, 130.0, 129.8, 129.2, 127.8, 127.1, 126.8, 126.8, 124.7, 124.4 (q, 264 Hz, CF_3), 38.1.

MS (ESI): *m/z* $[M+H]^+$ found 578.1113 calc. 578.1113.

EA: found C 45.82, H 2.969, N 7.19 calc. C 45.77, H 2.97, N 7.28.

(BnPhTrz)AuPh

In an oven dried Schlenk flask under argon **(BnPhTrz)BF_4** (1.00 equiv, 31.4 mg, 0.093 mmol) was dissolved in THF (2 mL) and was cooled to -78 °C. Potassium hexamethyldisilazide (1.05 equiv, 19.5 mg, 0.098 mmol) was added. After 10 min of stirring a precooled solution of (PPh_3)AuPh (1.00 equiv, 50 mg, 0.093 mmol) in THF (2 mL) was added. The reaction mixture was stirred under slow warm-up to room temperature for 16 h. The solvent was removed in vacuum and the crude product was washed with hexane (3 x 3 mL). After extraction with toluene (2 x 4 mL), filtration over celite and removing the solvent in vacuum the gold complex with minor impurities. Crystalization from a nearly saturated solution of the complex in CH_2Cl_2 layered with hexane yielded crystalline, colourless **(BnPhTrz)AuPh** (21%, 0.019 mmol, 10 mg).

^1H NMR (700 MHz, $CDCl_3$) δ (ppm) 7.76-7.75 (m, 4H, aryl H), 7.54-7.53 (m, 2H, aryl H), 7.50-7.48 (m, 3H, aryl H), 7.42-7.37 (m, 3H, aryl H), 7.21 (t, 2H, aryl H), 7.00 (t, 1H, aryl H), 5.81 (s, 2H, CH_2), 4.08 (s, 3H, N-CH_3).

$^{13}C\{^1H\}$ NMR (176 MHz, $CDCl_3$) δ (ppm) 180.1, 170.0, 148.3, 140.7, 134.6, 130.0, 129.7, 129.4, 129.1, 129.0, 129.0, 127.3, 127.2, 124.8, 58.9, 37.7.

MS (ESI): *m/z* $[M+H]^+$ found 446.0922 calc. 446.0926.

EA: found C 50.49, H 3.85 N 8.14 calc. C 50.49, H 3.85 N 8.03.

2 NMR Spectra

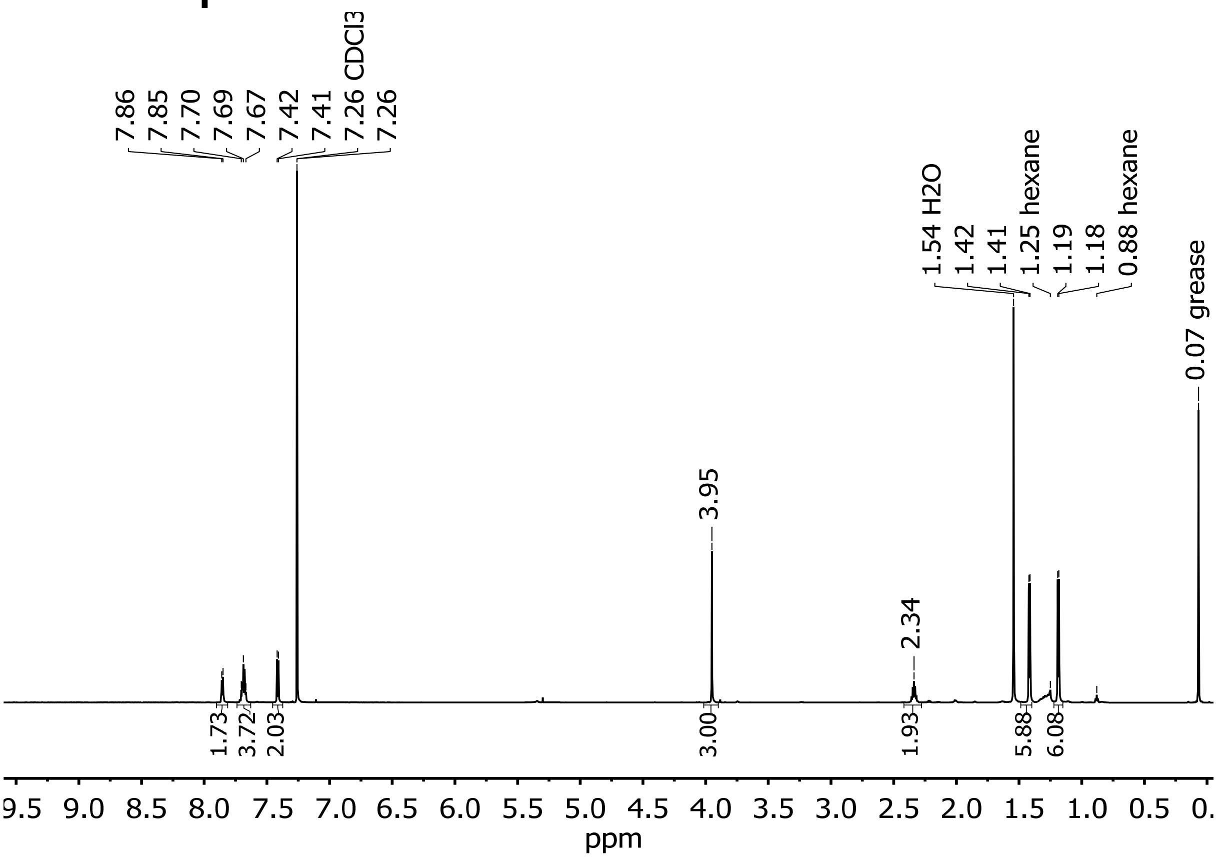

Figure S1. ^{1}H NMR spectrum of **(PhDippTrz)AuCl** in chloroform-d.

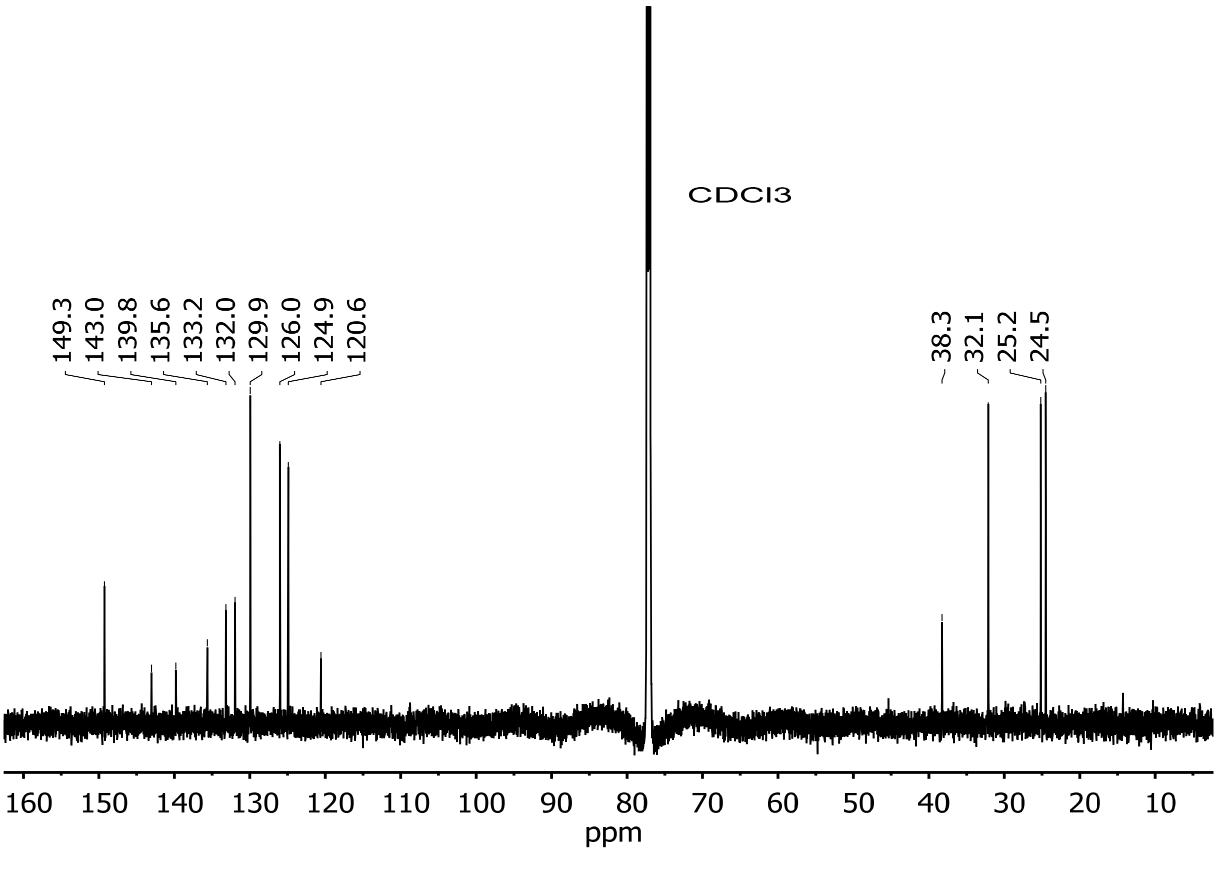

Figure S2. $^{13}C\{^{1}H\}$ NMR spectrum of **(PhDippTrz)AuCl** in chloroform-d.

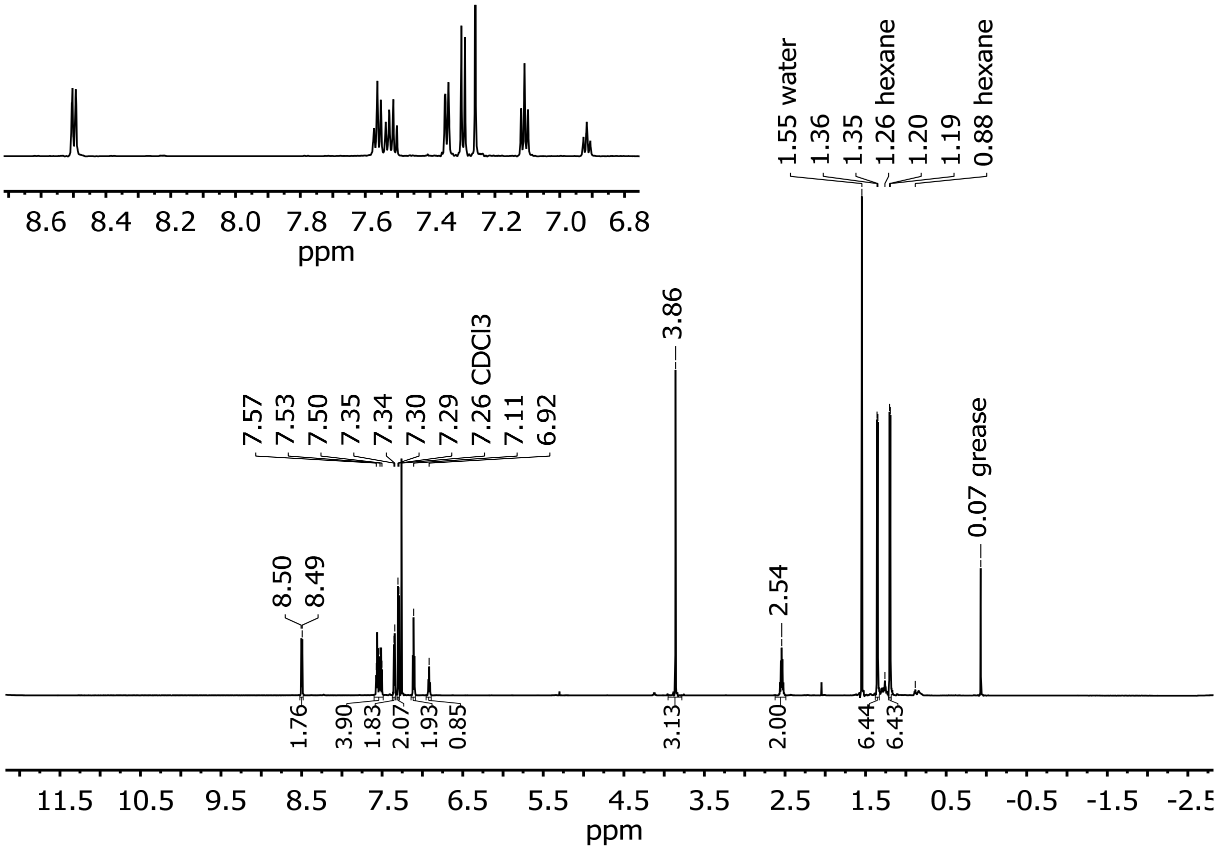

Figure S3. ^{1}H NMR spectrum of **(PhDippTrz)AuPh** in chloroform-d.

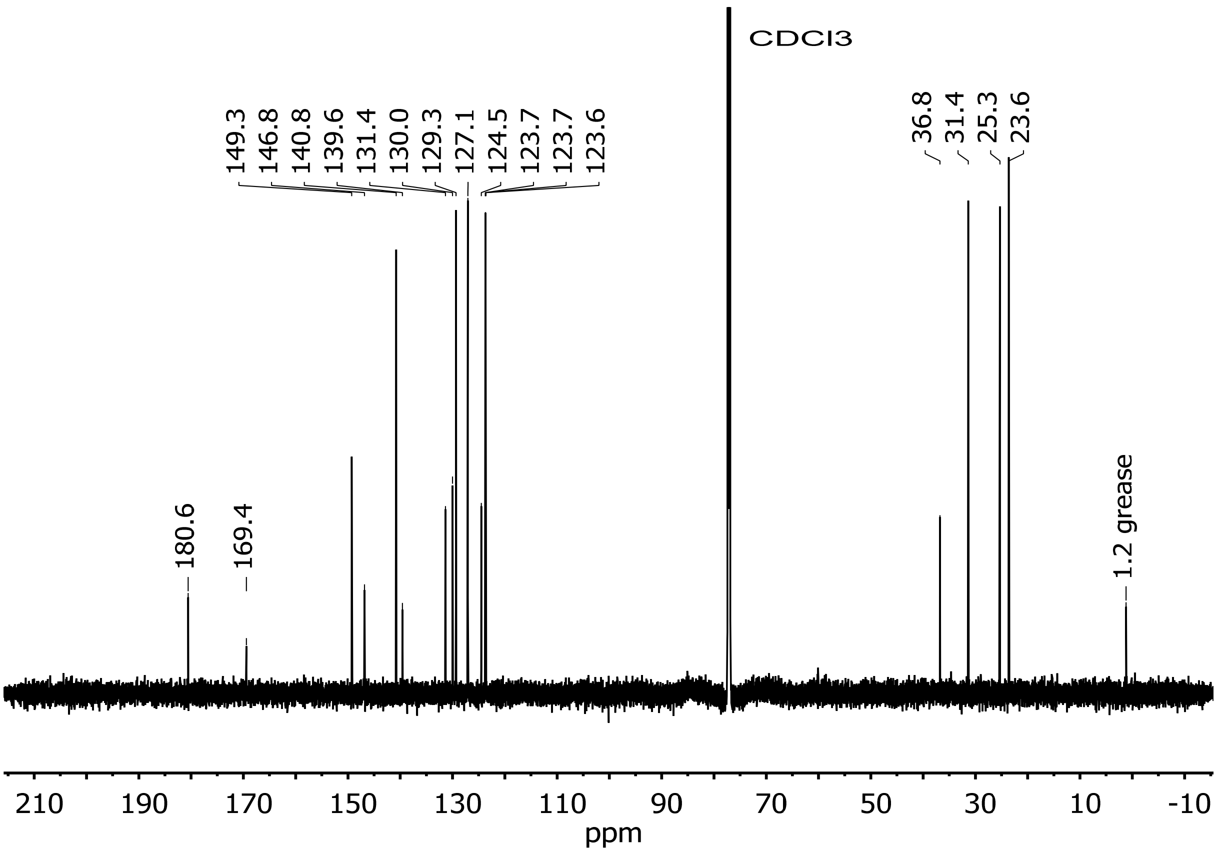

Figure S4. $^{13}C\{^{1}H\}$ NMR spectrum of **(PhDippTrz)AuPh** in chloroform-d.

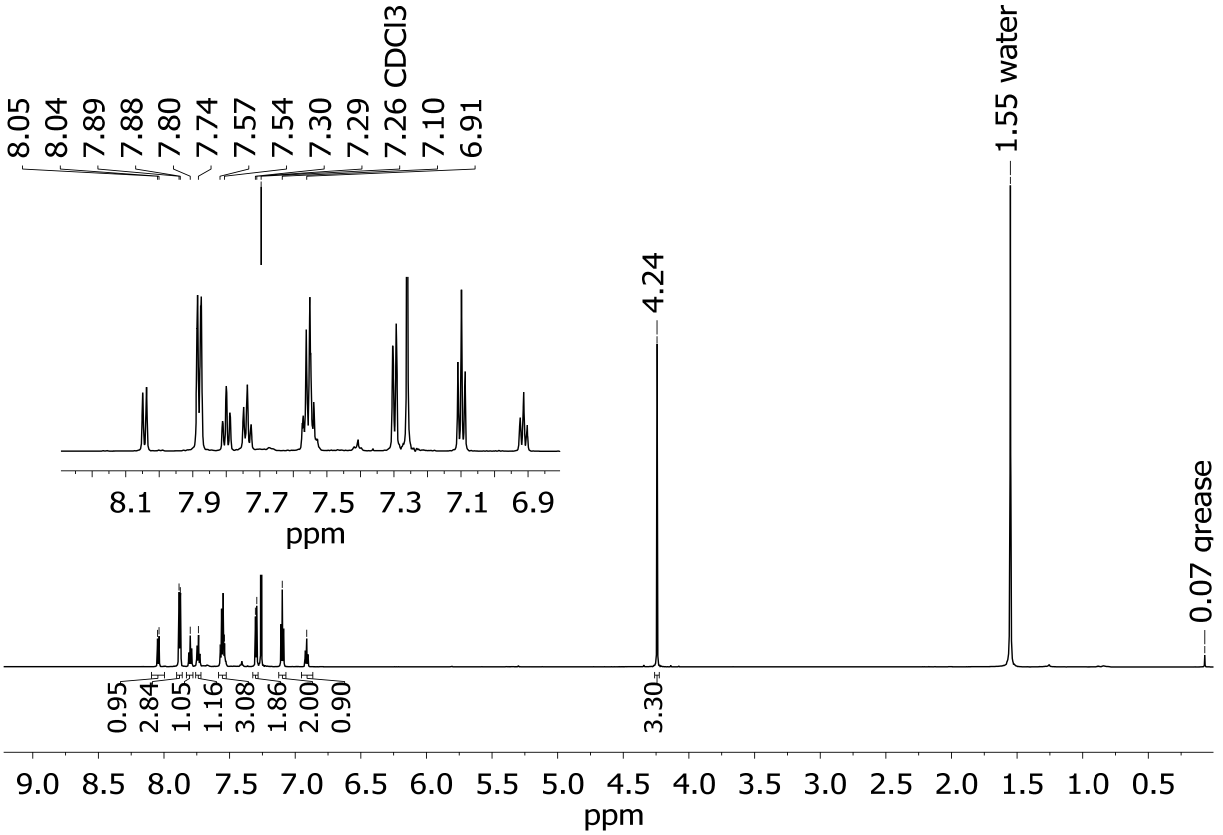

Figure S5. ^{1}H NMR spectrum of **(PhCF$_3$PhTrz)AuPh** in chloroform-d.

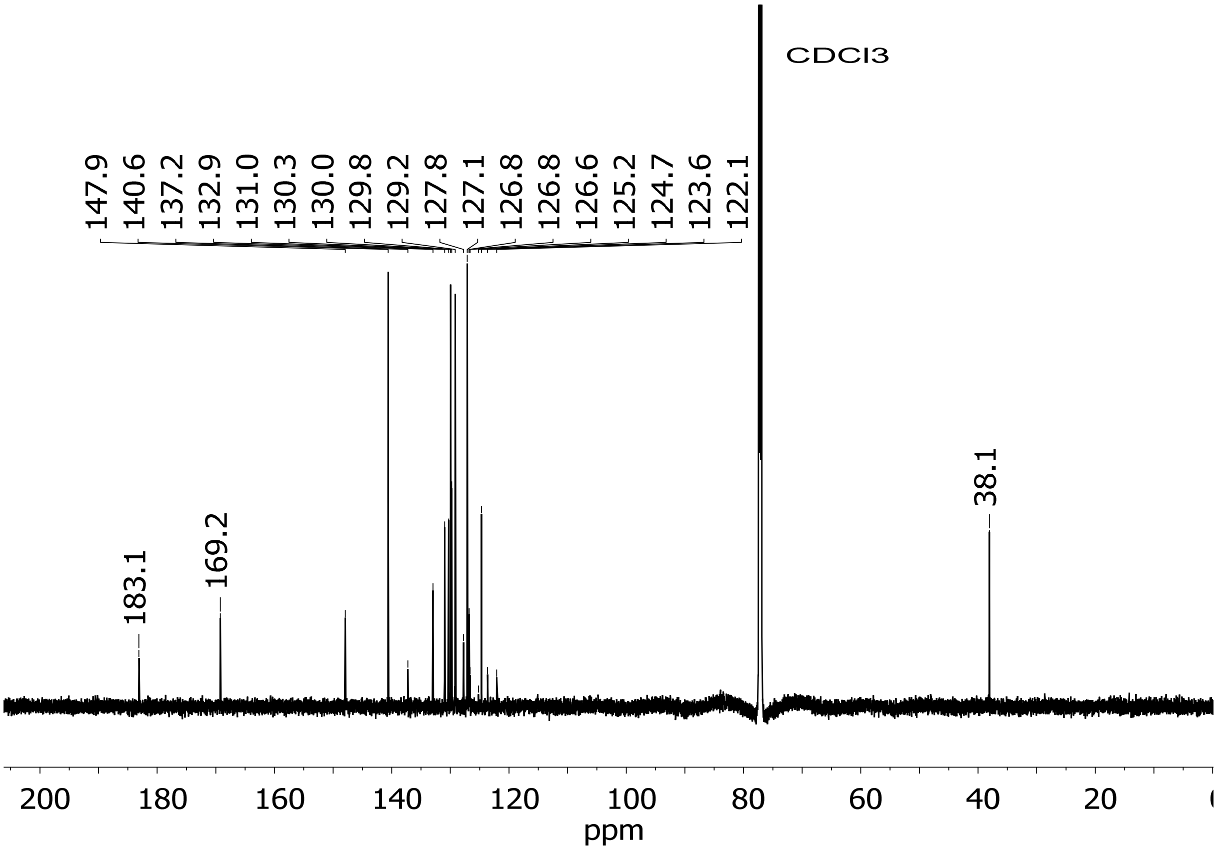

Figure S6. $^{13}C\{^1H\}$ NMR spectrum of **(PhCF$_3$PhTrz)AuPh** in chloroform-d.

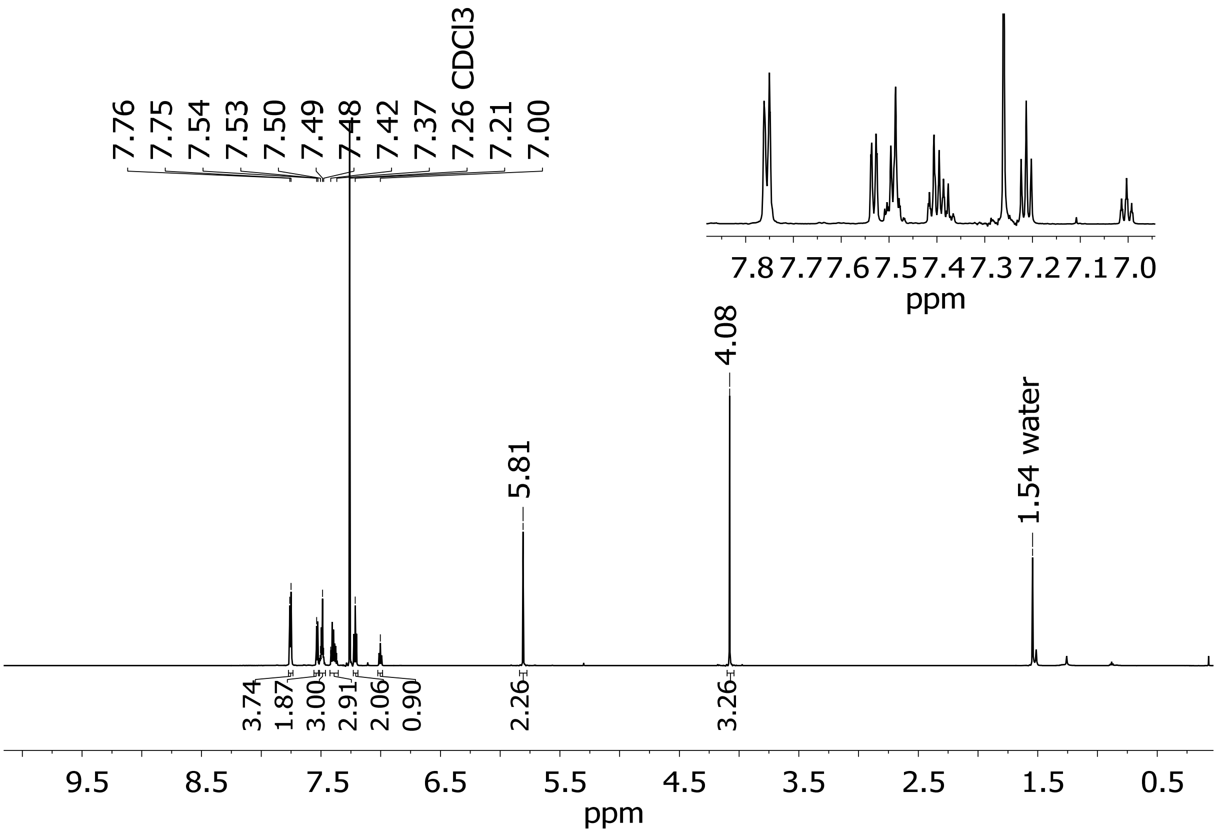

Figure S7. ^1H NMR spectrum of **(BnPhTrz)AuPh** in chloroform-d.

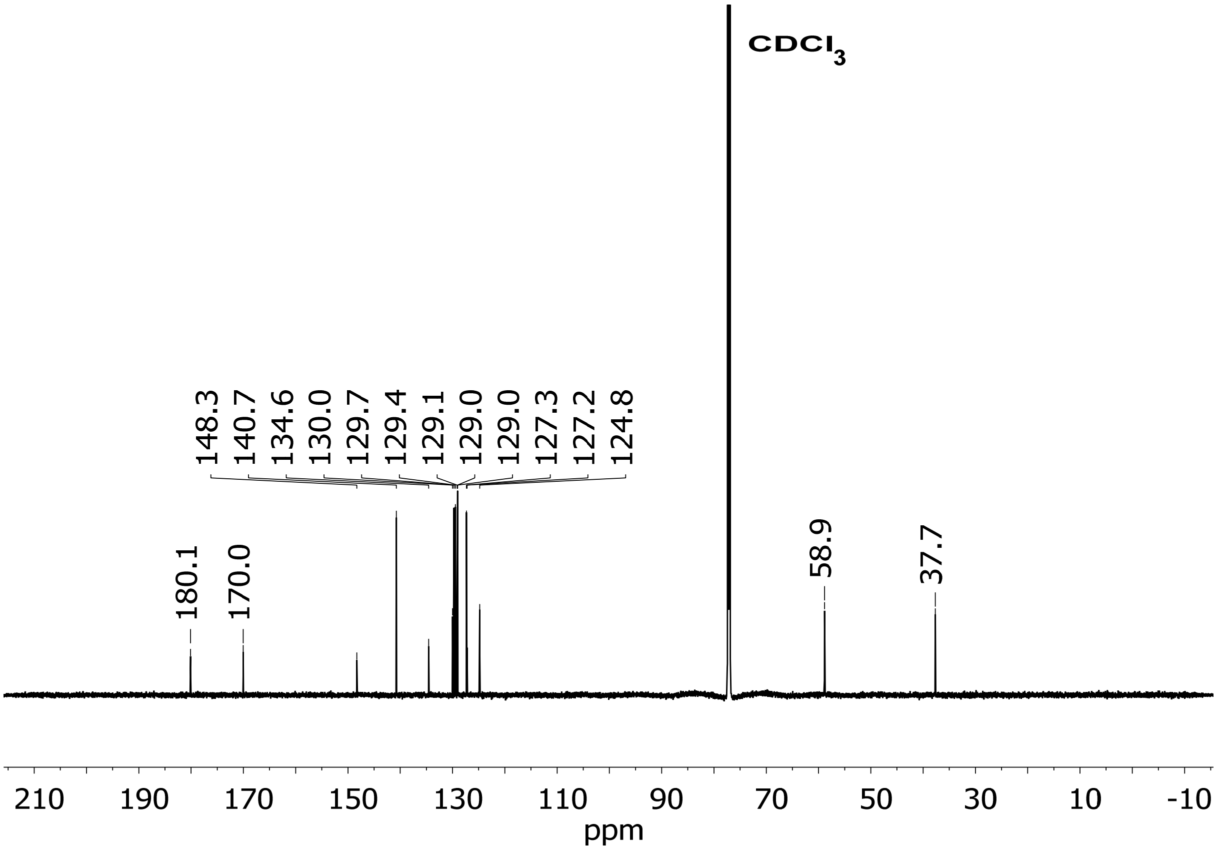

Figure S8. $^{13}C\{^{1}H\}$ NMR spectrum of (**BnPhTrz)AuPh** in chloroform-d.

3 Cyclic Voltammetry

Cyclic voltammograms were recorded with a PalmSens4 by working in freshly distilled and degassed MeCN (99.8% extra dry, Acros Organics) or degassed DMF (anhydrous, 99.8%, Sigma-Aldrich) with 0.1 M NBu_4PF_6 (dried, >99.0%, electrochemical grade, Fluka) as the supporting electrolyte. Concentrations of the selenium adducts and gold complexes were about 1.4-1.9 × 10^{-3} M. A three-electrode setup was used with glassy carbon as the working electrode, a coiled platinum wire as the counter electrode, and a coiled silver wire as the pseudoreference electrode. The HFc^*/HFc^{*+} couple was used as the internal reference for the selenium adducts. The HFc/HFc^+ couple was used as the internal reference for the gold complexes.

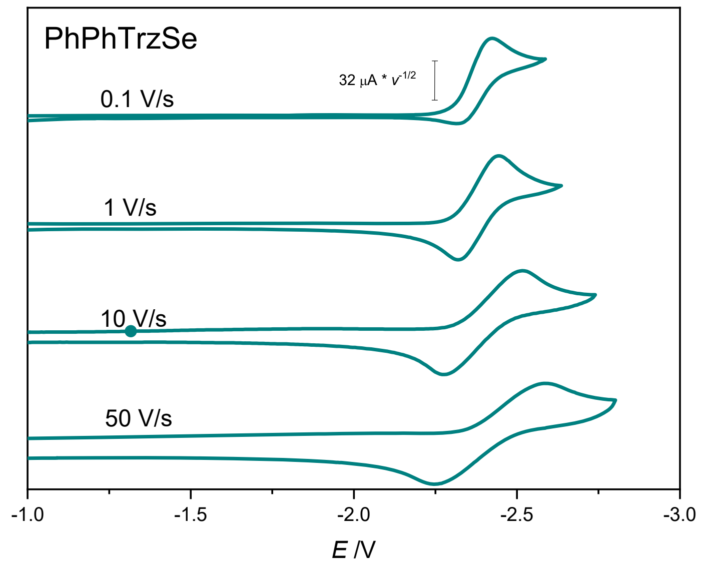

Figure S9. First electrochemical reduction of **PhPhTrzSe** at various scan rates. The current was scaled by multiplying with (scan rate)$^{-1/2}$ (in MeCN, electrolyte: NBu_4PF_6, electrode: glassy carbon, at scan rate of 50 V/s: IR drop compensation of 50 Ω).

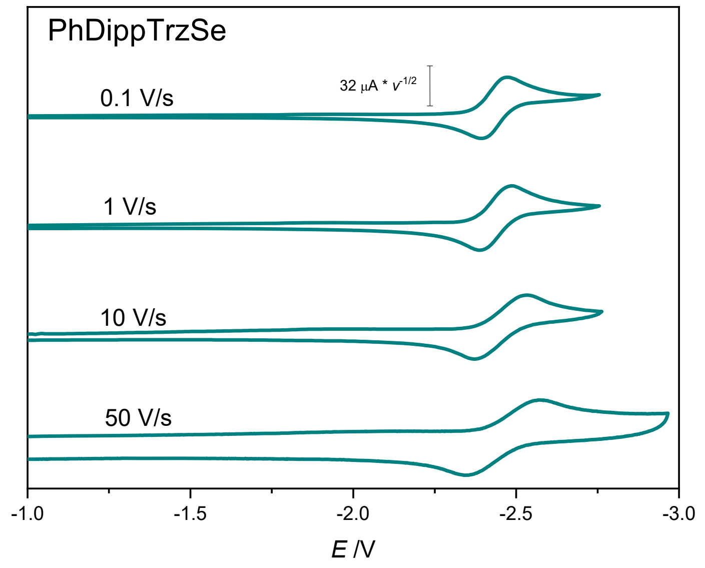

Figure S 10. First electrochemical reduction of **PhDippTrzSe** at various scan rates. The current was scaled by multiplying with (scan rate)$^{-1/2}$ (in MeCN, electrolyte: NBu_4PF_6, electrode: glassy carbon, at scan rate of 50 V/s: IR drop compensation of 50 Ω).

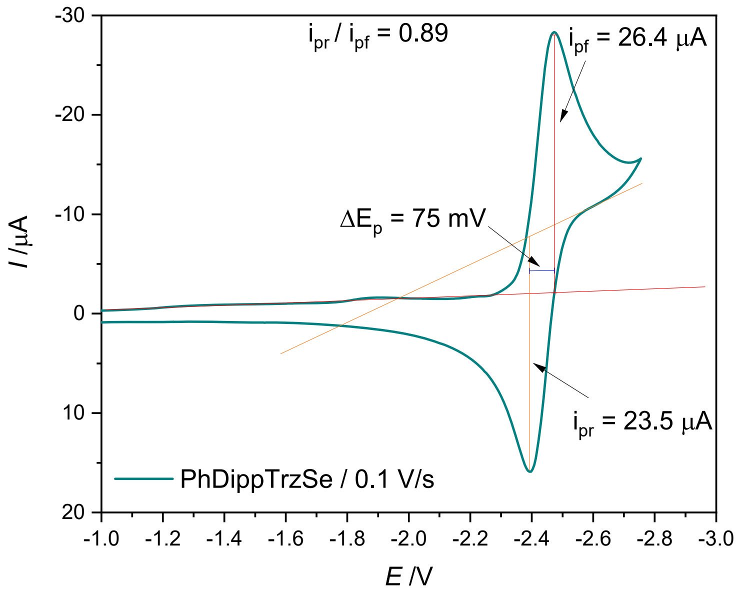

Figure S11. Analysis of the cyclic voltammogram of PhDippTrzSe under the aspect of reversibility (in MeCN, electrolyte: NBu_4PF_6, scan rate: 100 mV/s, electrode: glassy carbon).

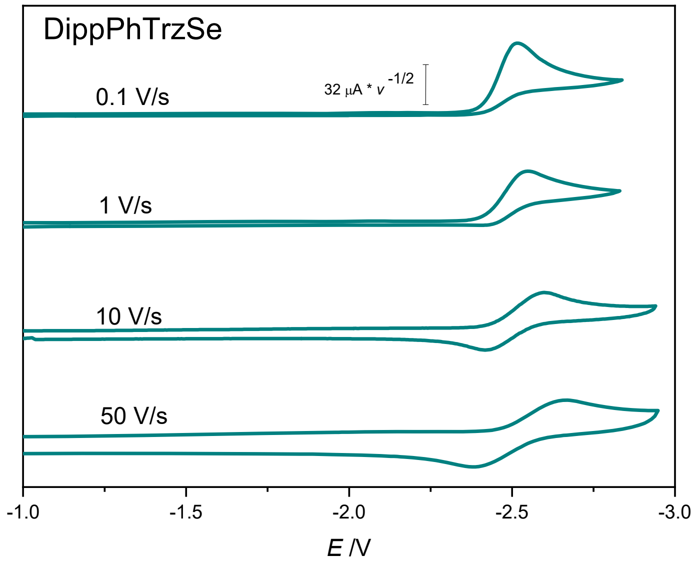

Figure S12. First electrochemical reduction of **DippPhTrzSe** at various scan rates. The current was scaled by multiplying with (scan rate)$^{-1/2}$ (in MeCN, electrolyte: NBu_4PF_6, electrode: glassy carbon, at scan rate of 50 V/s: IR drop compensation of 50 Ω).

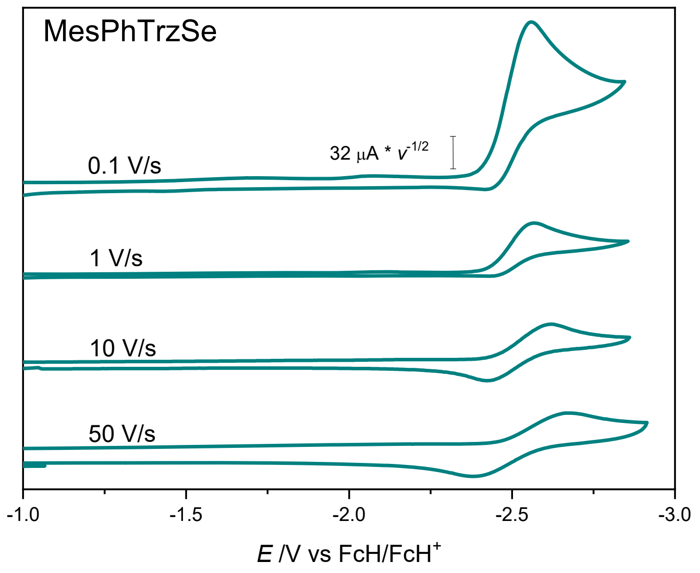

Figure S13. First electrochemical reduction of **MesPhTrzSe** at various scan rates. The current was scaled by multiplying with (scan rate)$^{-1/2}$ (in MeCN, electrolyte: NBu_4PF_6, electrode: glassy carbon, at scan rate of 50 V/s: IR drop compensation of 50 Ω).

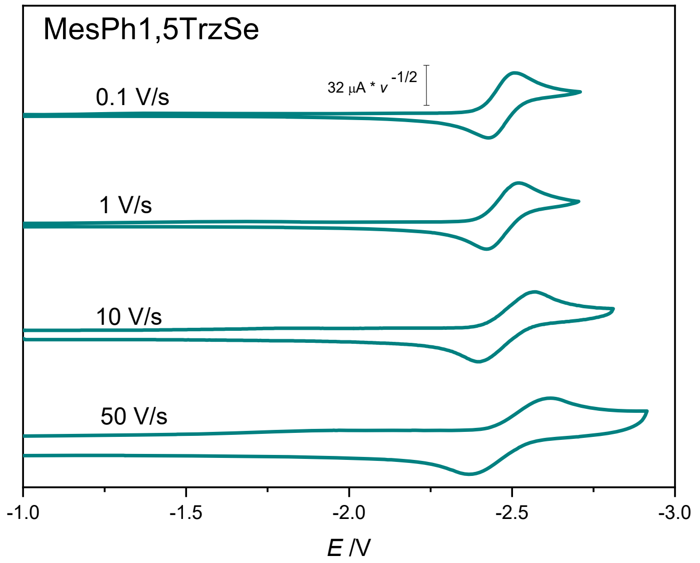

Figure S14. First electrochemical reduction of **MesPh1,5TrzSe** at various scan rates. The current was scaled by multiplying with (scan rate)$^{-1/2}$ (in MeCN, electrolyte: NBu_4PF_6, electrode: glassy carbon, at scan rate of 50 V/s: IR drop compensation of 50 Ω).

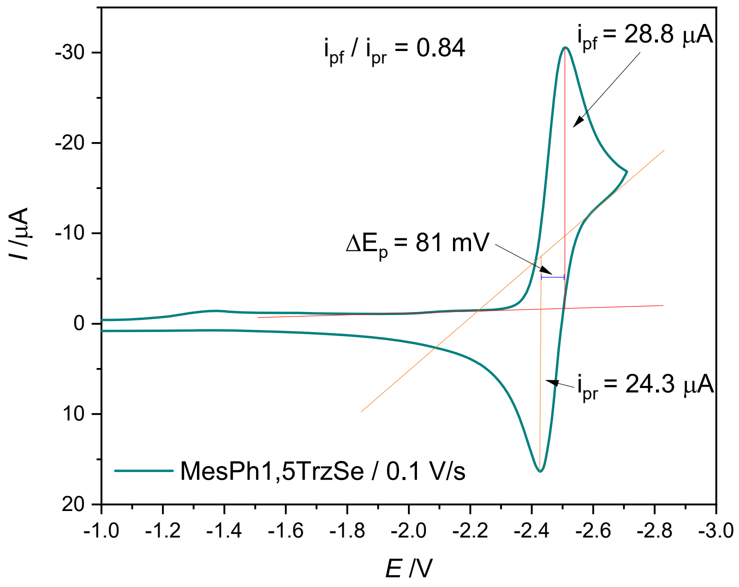

Figure S15: Analysis of the cyclic voltammogram of PhDippTrzSe under the aspect of reversibility (in MeCN, electrolyte: NBu_4PF_6, scan rate: 100 mV/s, electrode: glassy carbon).

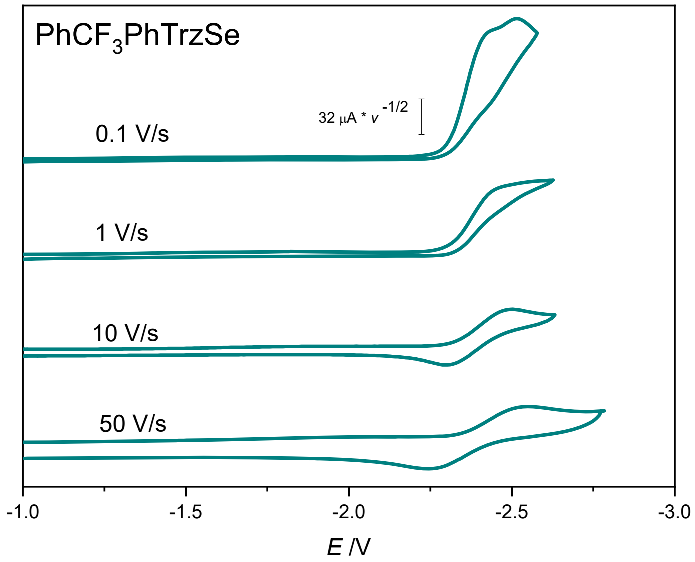

Figure S16. First electrochemical reduction of **$PhCF_3PhTrzSe$** at various scan rates. The current was scaled by multiplying with (scan rate)$^{-1/2}$ (in MeCN, electrolyte: NBu_4PF_6, electrode: glassy carbon, at scan rate of 50 V/s: IR drop compensation of 50 Ω).

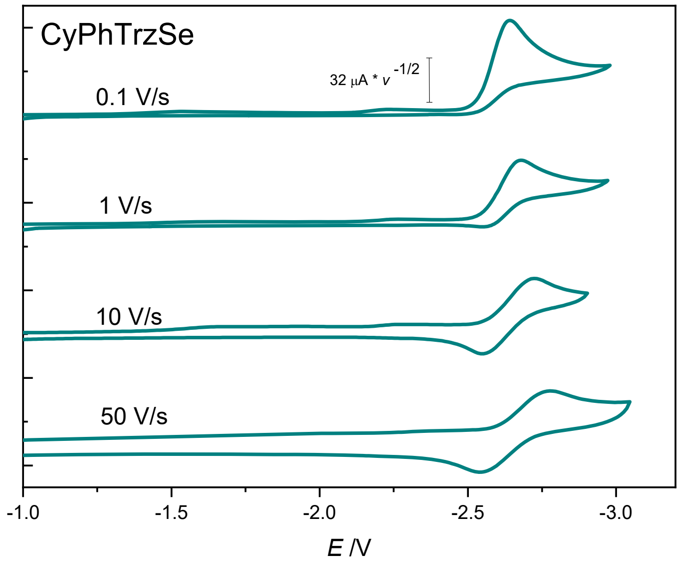

Figure S17. First electrochemical reduction of **CyPhTrzSe** at various scan rates. The current was scaled by multiplying with (scan rate)$^{-1/2}$ (in MeCN, electrolyte: NBu_4PF_6, electrode: glassy carbon, at scan rate of 50 V/s: IR drop compensation of 50 Ω).

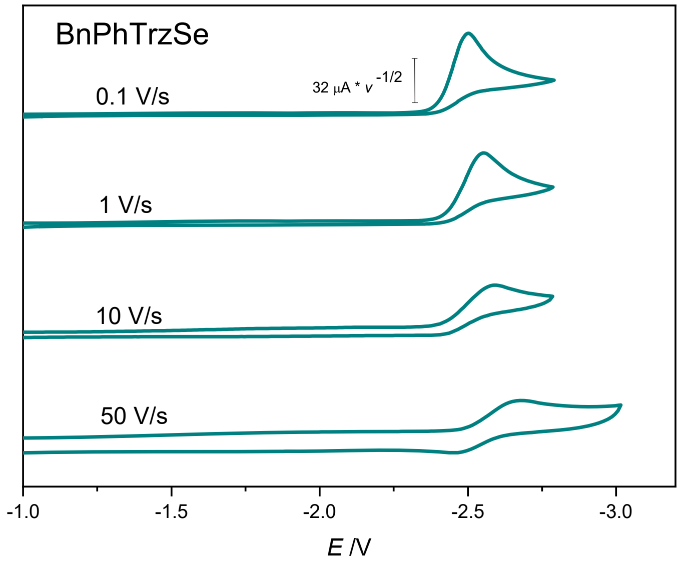

Figure S18. First electrochemical reduction of **BnPhTrzSe** at various scan rates. The current was scaled by multiplying with (scan rate)$^{-1/2}$ (in MeCN, electrolyte: NBu_4PF_6, electrode: glassy carbon, at scan rate of 50 V/s: IR drop compensation of 50 Ω).

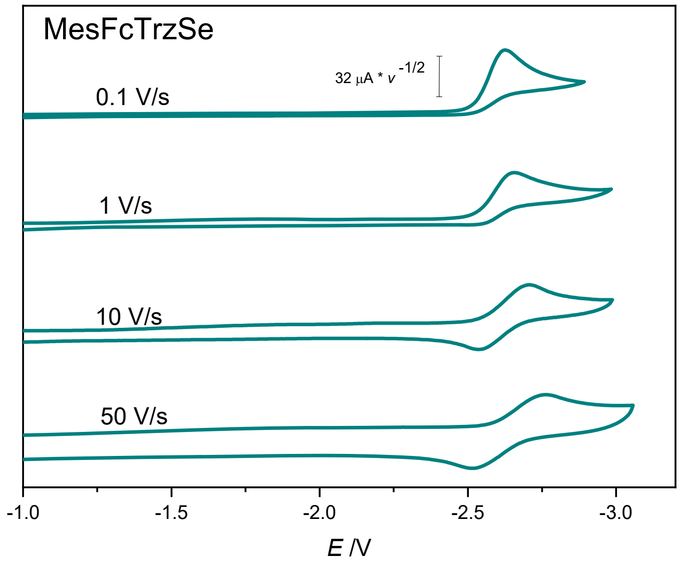

Figure S19. First electrochemical reduction of **MesFcTrzSe** at various scan rates. The current was scaled by multiplying with (scan rate)$^{-1/2}$ (in MeCN, electrolyte: NBu_4PF_6, electrode: glassy carbon, at scan rate of 50 V/s: IR drop compensation of 50 Ω).

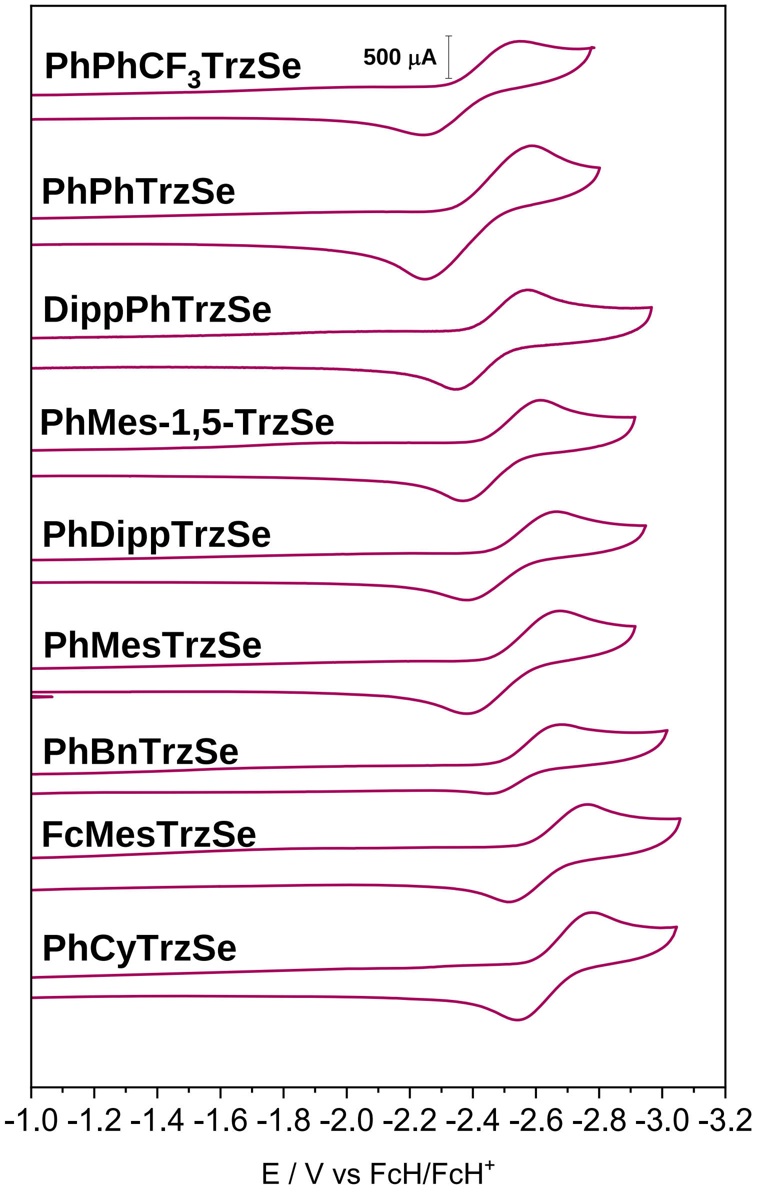

Figure S20. First reduction of the selenium adducts at 50 V/s. (in MeCN, electrolyte: NBu_4PF_6, electrode: glassy carbon)

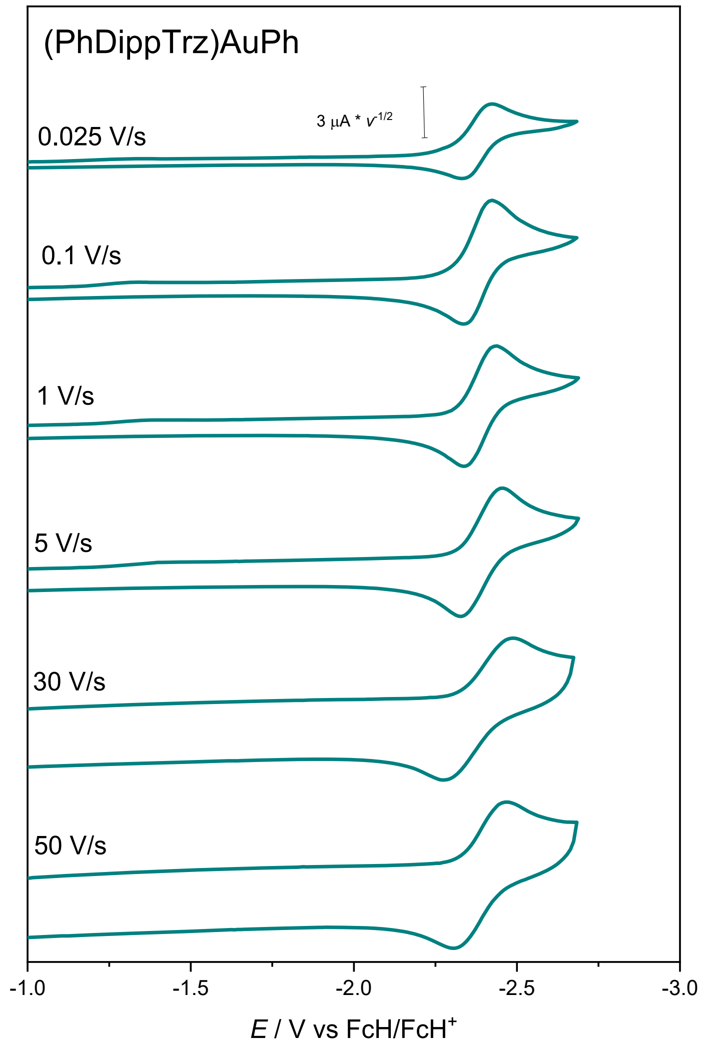

Figure S21. First electrochemical reduction of (**PhDippTrz)AuPh** at various scan rates. The current was scaled by multiplying with (scan rate)$^{-1/2}$ (in DMF, electrolyte: NBu_4PF_6, electrode: glassy carbon)

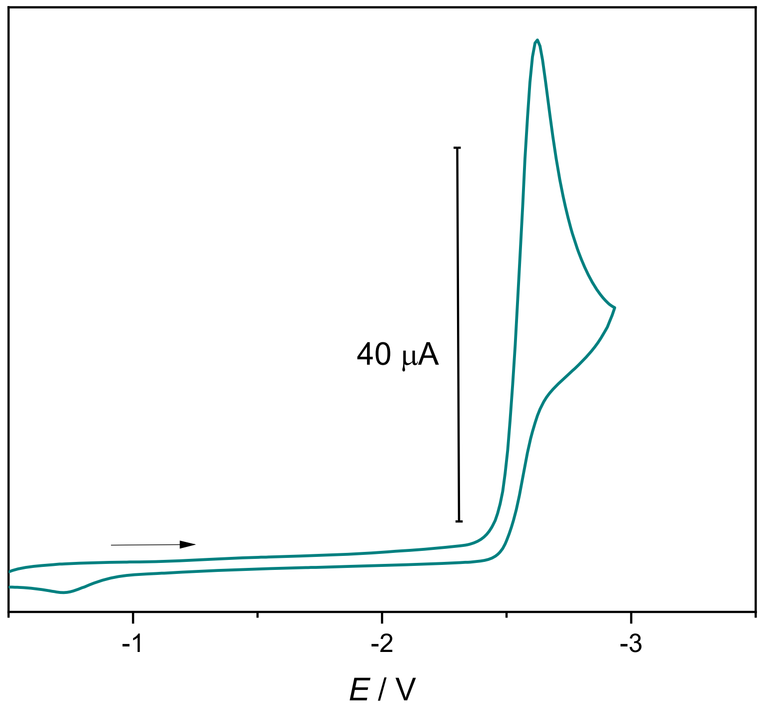

Figure S22. First electrochemical reduction of **(BnPhTrz)AuPh**. (scan rate of 0.1 V/s, in DMF, electrolyte: NBu_4PF_6, electrode: glassy carbon)

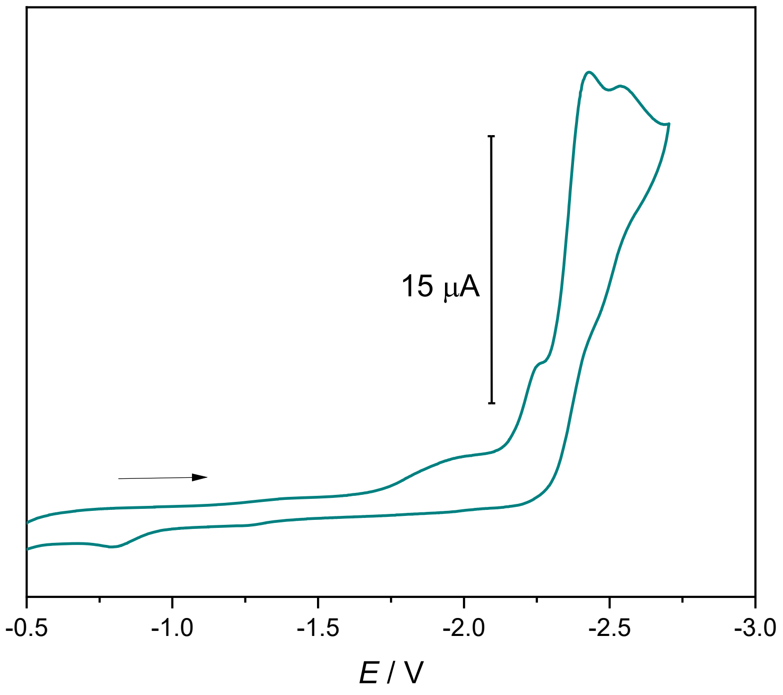

Figure S203. First electrochemical reduction of **(PhCF$_3$PhTrz)AuPh**. (scan rate of 0.1 V/s, in DMF, electrolyte: NBu_4PF_6, electrode: glassy carbon)

4 UV/Vis/NIR Spectroscopy and Spectroelectrochemistry

UV/Vis/NIR spectra were recorded with an J&M TIDAS spectrophotometer Spectroelectrochemical measurements were carried out in an optically transparent thin-layer electrochemical (OTTLE) cell[4] (CaF_2 windows) with a gold-mesh working electrode (100 mesh woven from 0.064 mm diameter wire; 99.99% (metals basis)), a platinum-mesh counter electrode, and a silver-foil pseudoreference electrode. Anhydrous and degassed acetonitrile (H_2O ≤ 0.005%, puriss., Sigma Aldrich) distilled from P_2O_5 or anhydrous or degassed DMF (anhydrous, 99.8%, Sigma-Aldrich) with 0.1 M NBu_4PF_6 as electrolyte was used as the solvent.

4.1 Supplementary Spectra

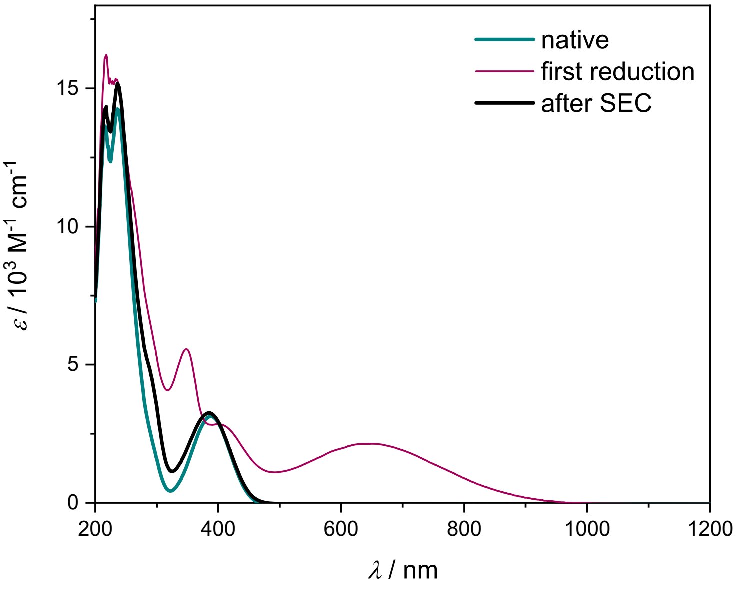

Figure S214. Comparison of the UV/VIS/NIR spectra of **PhDippTrzSe** before, during and after the spectroelectrochemistry in MeCN (0.1 M NBu_4PF_6). The electrolysis was stopped after the spectrum did not change anymore.

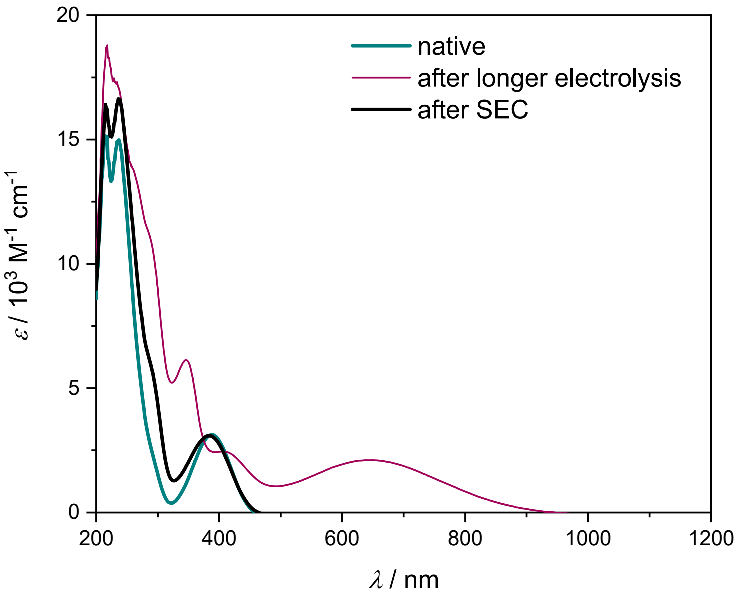

Figure S225. Comparison of the UV/VIS/NIR spectra of **PhDippTrzSe** before, during and after the spectroelectrochemistry in MeCN (0.1 M NBu_4PF_6). The electrolysis was stopped after the spectrum did not change anymore for three minutes.

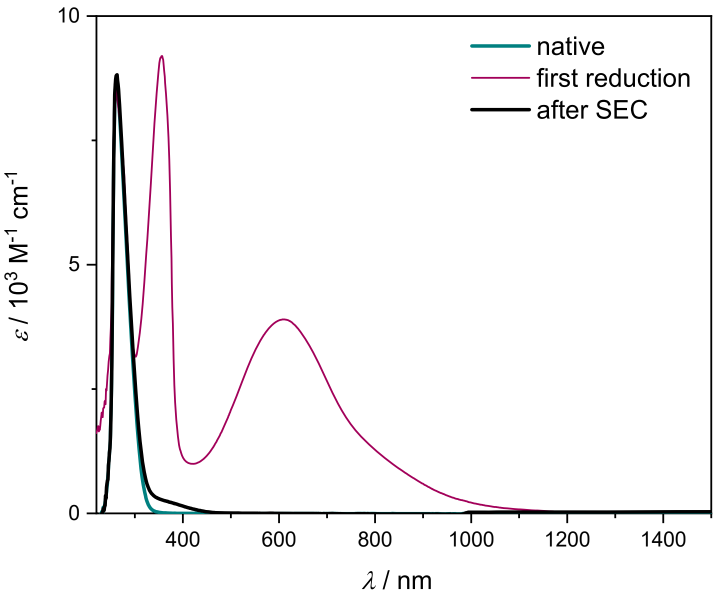

Figure S236. Comparison of the UV/VIS/NIR spectra of **(PhDippTrz)AuPh** before, during and after the spectroelectrochemistry in DMF (0.1 M NBu_4PF_6). The electrolysis was stopped after the spectrum did not change anymore for three minutes.

4.2 TD-DFT Simulation

4.2.1 DFT Calculation for **PhDippTrzSe** (singlet state)

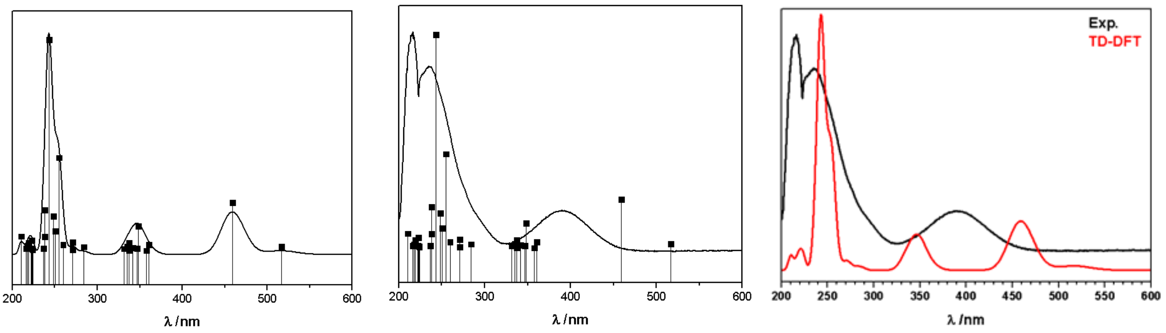

Figure S247. Calculated TD-DFT spectrum of **PhDippTrzSe** with discrete transitions (left), experimental spectrum with discrete calculated transitions (middle) and experimental (black) and calculated TD-DFT spectrum (red) (left).

Table S1. TD-DFT transitions for **PhDippTrzSe.**

State	Difference density (iso value 0.002)	Transition	Calculated Transition energy	Osc. strength	Exp. transition energy	Molar absorption coefficient 10^3
1	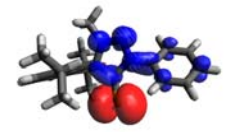	HOMO-1 -> LUMO (0.64) HOMO-> LUMO (0.34)	516.5	0.0043	387	3.1
2	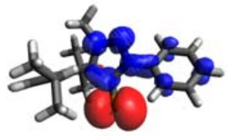	HOMO-1 -> LUMO (0.34) HOMO -> LUMO (0.63)	459.0	0.0484		
5	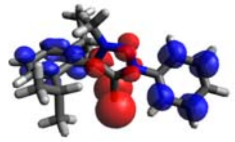	HOMO-1 -> LUMO+1 (0.86)	348.4	0.0249	236	14.3
14	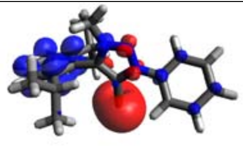	HOMO-4 -> LUMO (0.57) HOMO -> LUMO +5 (0.27)	254.6	0.0940	218	13.6
16	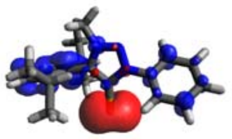	HOMO-4 -> LUMO (0.31) HOMO -> LUMO +5 (0.40)	243.0	0.2135		

Table S2. Selected molecular orbitals for **PhDippTrzSe**.

HOMO-1	HOMO	LUMO	LUMO+5
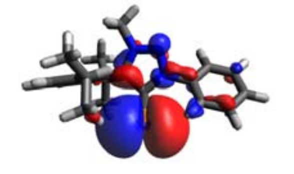	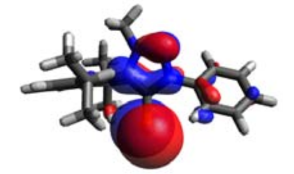	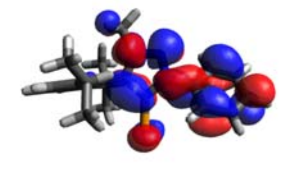	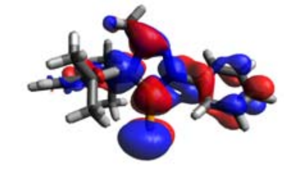

Table S3. Orbital energies for **PhDippTrzSe_1red** (doublet state).

Orbital No.	HOMO/LUMO	Energy (Eh)	Energy (eV)
98	HOMO-4	-0.263027	-7.1573
99	HOMO-3	-0.258008	-7.0208
100	HOMO-2	-0.245084	-6.6691
101	HOMO-1	-0.197476	-5.3736
102	HOMO	-0.191335	-5.2065
103	LUMO	-0.055791	-1.5182
104	LUMO+1	-0.021658	-0.5894
105	LUMO+2	-0.017549	-0.4775
106	LUMO+3	-0.014019	-0.3815
107	LUMO+4	-0.012782	-0.3478
108	LUMO+5	0.018706	0.5090
109	LUMO+6	0.029319	0.7978

4.2.2 DFT Calculation for **PhDippTrzSe_1red** (doublet state)

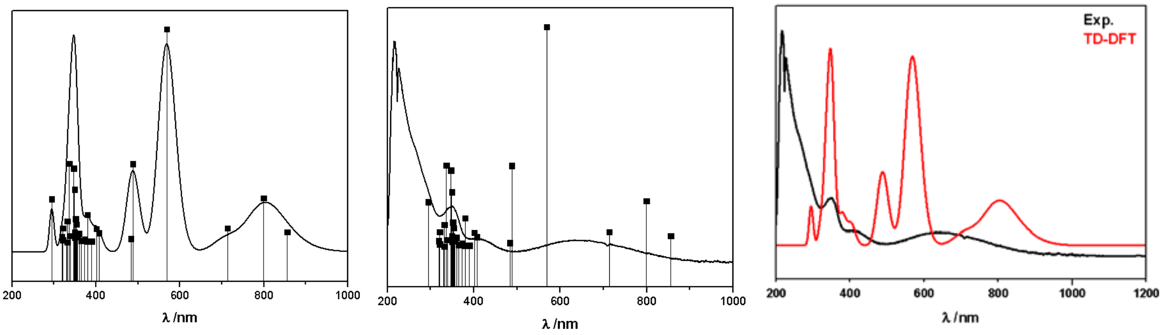

Figure S258. Calculated TD-DFT spectrum of **PhDippTrzSe_1red** with discrete transitions (left), experimental spectrum with discrete calculated transitions (middle) and experimental (black) and calculated TD-DFT spectrum (red) (left).

Table S4. TD-DFT transitions for **PhDippTrzSe_1red.**

State	Difference density (iso value 0.002)	Transition	Calc. Transition energy	Osc. strength	Exp. transition energy	Molar abs. coeff.
3	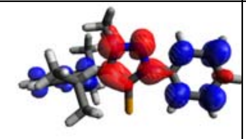	HOMOα -> LUMOα+1 (0.99)	855.4	0.0049	645	2.1
1	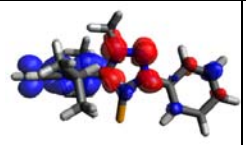	HOMOα -> LUMOα (0.90)	799.2	0.0200		
4	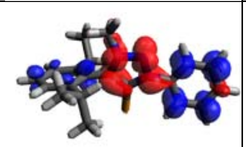	HOMOα -> LUMOα+3 (0.92)	568.4	0.0956	410	2.8
5	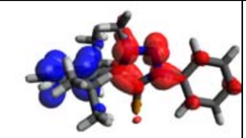	HOMOβ -> LUMOβ (0.87)	487.9	0.0354	351	5.6
28	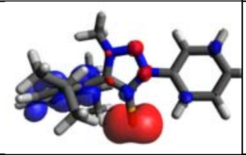	HOMOα-2 -> LUMOα+2 (0.21)<br>HOMOβ-1 -> LUMOβ+3 (0.52)	346.4	0.0334	236	15.3
17	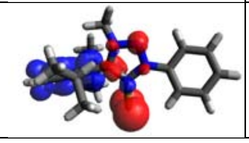	HOMOβ-2 -> LUMOβ (0.19)<br>HOMOβ-1 -> LUMOβ+1 (0.25)	336.1	0.0354	218	16.2

Table S5. Selected molecular orbitals for **PhDippTrzSe_1red**.

	HOMOβ-2	HOMOβ-1	HOMOβ	LUMOβ	LUMOβ+1
	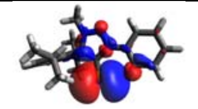	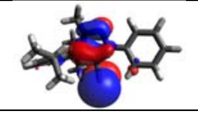	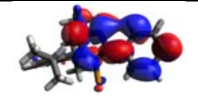	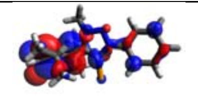	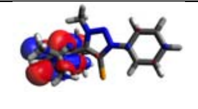
HOMOα-2	HOMOα	LUMOα	LUMOα+1	LUMOα+2	LUMOα+3
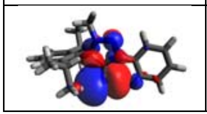	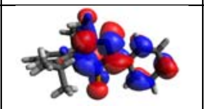	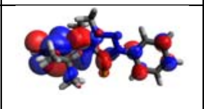	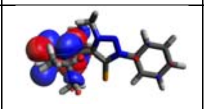	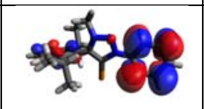	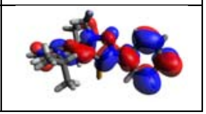

Table S6. Orbital energies for **PhDippTrzSe_1red** (doublet state).

Spin up Orbitals alpha				Spin down orbitals beta			
Orbital No.	Orbital	Energy (Eh)	Energy (eV)	Orbital No.	Orbital	Energy (Eh)	Energy (eV)
100	HOMOα-3	-0.216977	-5.9043	100	HOMOβ-2	-0.201328	-5.4784
101	HOMOα-2	-0.164608	-4.4792	101	HOMOβ-1	-0.163121	-4.4387
102	HOMOα-1	-0.160979	-4.3805	102	HOMOβ	-0.153073	-4.1653
103	HOMOα	-0.093157	-2.5349	103	LUMOβ	-0.017598	-0.4789

104	LUMOα	0.002123	0.0578	104	LUMOβ+1	0.003046	0.0829
105	LUMOα+1	0.009688	0.2636	105	LUMOβ+2	0.009969	0.2713
106	LUMOα+2	0.012491	0.3399	106	LUMOβ+3	0.014429	0.3926
107	LUMOα+3	0.020342	0.5535	107	LUMOβ+4	0.034790	0.9467

5 Electron Paramagnetic Resonance (EPR)

5.1 General Remarks

EPR spectra at X-band frequency (ca. 9.5 GHz) were obtained with a Magnettech MS-5000 benchtop EPR spectrometer equipped with a rectangular TE 102 cavity. The measurements were carried out in synthetic quartz glass tubes. For EPR spectroelectrochemistry a three-electrode setup was employed using two Teflon coated platinum wires (0.005" bare, 0.008" coated) as working (or a Teflon coated gold wire (0.003" bare, 0.0055" coated) as working electrode) and counter electrode and a Teflon-coated silver wire (0.005" bare, 0.007" coated) as pseudoreference electrode.
The EPR spectra have been simulated with MatLab R2012b using Easyspin 5.2.23.[5]

5.2 Simulation

PhDippTrzSe

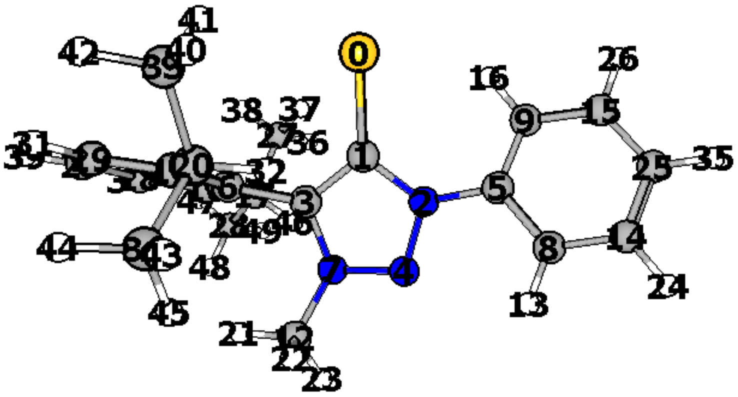

Figure S269. Numbering scheme for the table below.

Table S7. Computed hyperfine coupling constants (HFCC) in MHz from EPR calculation (B3LYP/def2-TZVP).

Atom	HFCC / MHz	Atom	HFCC / MHz
0Se	0.78	21H	2.13
2N	5.76	22H	24.7
4N	20.40	23H	7.48
7N	22.14	21H	2.13
16H	3.27	24H	2.08l
		35H	6.19

Table S8. HFCC used for simulation of the EPR spectrum of **[PhDippTrzSe]$^{\bullet-}$** with Easyspin.

Nucleus	HFCC / MHz	Nucleus	HFCC / MHz
^{14}N	7.97	^{1}H	19.73
^{14}N	26.99	^{1}H	27.26
^{14}N	26.86	^{1}H	27.15
^{1}H	19.92	^{1}H	27.15

g-value used for simulation: 2.0033

Table S9. HFCC used for simulation of the EPR spectrum of the electrochemically generated **[(PhDippTrz)AuPh]$^{\bullet-}$** with Easyspin.

Nucleus	HFCC / MHz	Nucleus	HFCC / MHz
^{14}N	17.83	^{1}H	12.52
^{14}N	17.11	^{1}H	12.52
^{14}N	8.91	^{1}H	1.78
^{1}H	5.92	^{1}H	1.78
^{1}H	5.92		

g-value used for simulation: 2.004

Table S10. HFCC used for simulation of the EPR spectrum of the chemically reduced **[(PhDippTrz)AuPh]$^{\bullet-}$** with Easyspin.

Nucleus	HFCC / MHz	Nucleus	HFCC / MHz
^{14}N	18.0	^{1}H	5.7
^{14}N	12.0	^{1}H	12.05
^{14}N	8.7	^{1}H	12.05
^{1}H	5.7	^{1}H	12.0
^{1}H	5.7		

g-value used for simulation: 2.0023

6 Single-Crystal X-Ray Diffraction Analysis

Single crystals suitable for X-ray diffraction analysis were grown by diffusion of hexane into a solution of the substance in dichloromethane.

X-ray data of **(PhDippTrz)SeAuPh** were collected on a STOE STADIVARI using $Mo_{K\alpha}$ radiation (λα = 0.71073 Å). The data were collected by rotation method (omega scans). The data were scaled and reduced using the STOE X-Area software. The structure was solved by intrinsic phasing using SHELXT-2014/7. They were refined by full matrix least-squares using SHELXL-2014/7 and was refined on F^2. Non-hydrogen atoms were refined anisotropically.[6] CCDC 2052185 contains the supplementary crystallographic data for this paper.

Table S11. Crystallographic data

	**(PhDippTrz)AuPh**
Chemical formula	C_{27} H_{30} Au N_3
*M*r	593.5
Crystal system	monoclinic
Space group	$P2_1/n$
a (Å)	11.3995(5)
b (Å)	16.1806(6)
c (Å)	13.1819(6)
α (°)	90
β (°)	105.533(4)
γ (°)	90
V (Å^3)	2342.61(18)
Z	4
Densitiy (g cm^{-3})	1.683
F(000)	1168
Radiation Type	MoK$_\alpha$
μ (mm^{-1})	6.299
Crystal size	0.2 x 0.1 x 0.1
Meas. Refl.	40044
Indep. Refl.	4776
Obsvd. [$I > 2\sigma(I)$] refl.	3450
R_{int}	0.0777
R [$F^2 > 2\sigma(F^2)$], wR(F^2), S	0.0328, 0.0778, 0.980
Δρmax, Δρmin (e Å^{-3})	1.912, -1.079

7 Correlations

It is known that a correlation of the formal redox potentials of a series of structurally similar molecules and their frontier orbital levels or electron affinities can exist.[7]

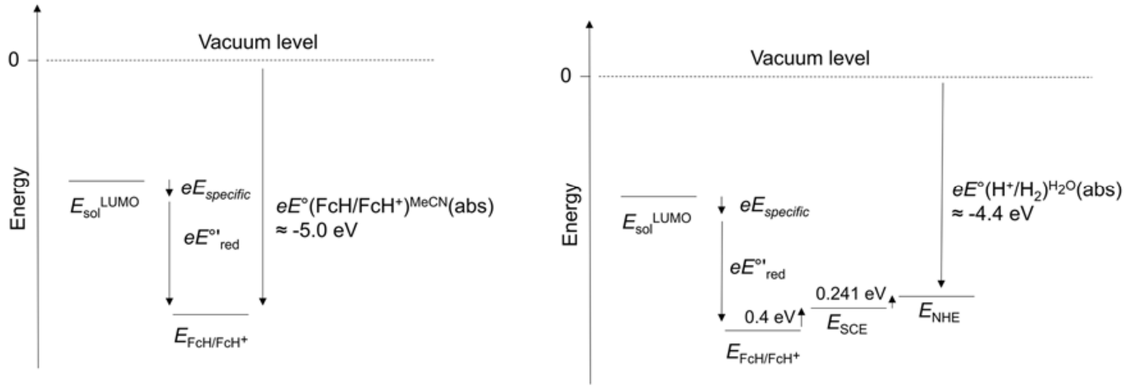

Figure S30. Schematic energy diagram describing the relationship between the formal reduction potential $E°'_{red}$ and the LUMO levels in acetonitrile E_{sol}^{LUMO} of the selenium adducts. (*e*: unit charge, $E_{specific}$: energy term including activity of the molecules (E°' -> E°), entropic changes and specific interactions of molecules in solution). The determination of the absolute electrode potential $E°(FcH/FCH^+)MeCN(abs)$ is determined from the abolute potential of the normal hydrogen electrode $E°(H^+/H_2)^{H_2O}(abs)$[8] and the values referencing the saturated calomel electrode[9] and the FcH/FcH$^+$ potential[10]).

Often the easy to obtain electrochemical values are used to estimate the orbital energies or the ionization energies/electron affinities. This must be interpreted with care, as apart from the redox potential further effects influence the absolute energy as entropic changes or specific interactions of the solvated molecules (summarized here as $E_{specific}$) (Figure S) (Formula (S1)).[11]

$$-E_{\mathrm{sol}}^{\mathrm{LUMO}} = e(E°'_{\mathrm{red}} + 5.0\ \mathrm{V} + E_{\mathrm{specific}}) \qquad \text{(S1)}$$

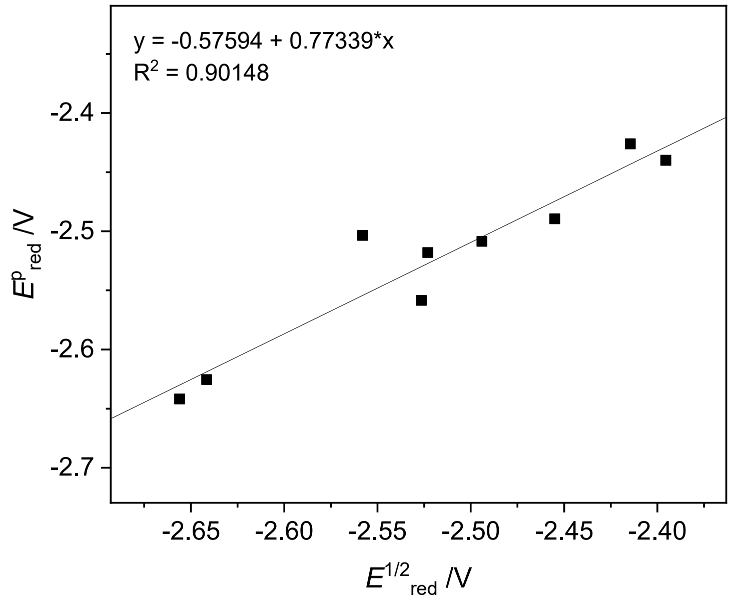

Figure S271. Correlation of the peak potential E^{p}_{red} and the half-wave potential $E^{1/2}_{red}$.

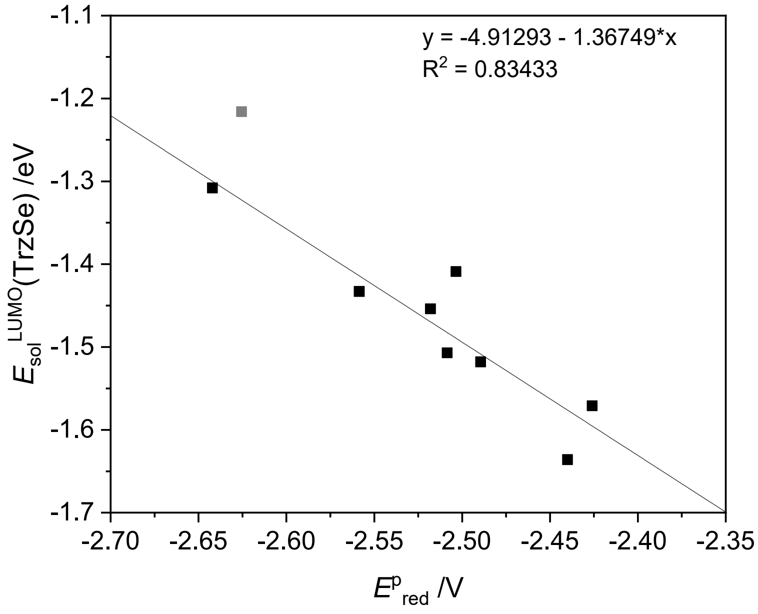

Figure S282. Correlation of E_{sol}^{LUMO}(TrzSe) and E^{p}_{red}. **MesFcTrzSe** was treated as an outlier.

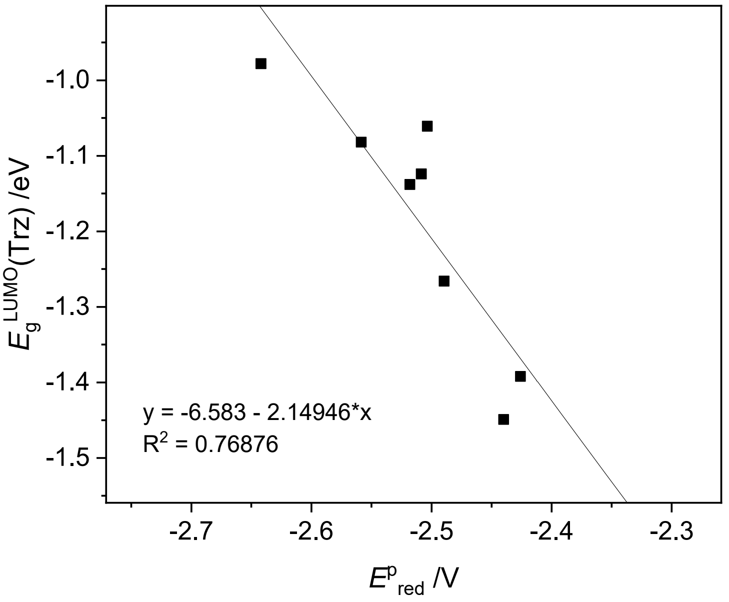

Figure S293. Correlation of E_{sol}^{LUMO}(Trz) and $E^{1/2}_{red}$. **MesFcTrzSe** was treated as an outlier.

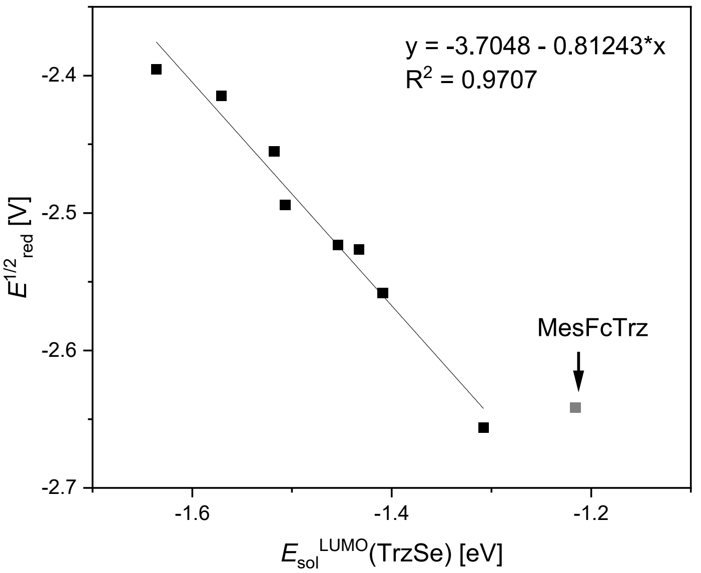

Figure S304. Correlation of $E^{1/2}_{red.}$ and E_{sol}^{LUMO}(TrzSe). **MesFcTrzSe** was treated as an outlier.

8 DFT

All density functional theory (DFT) calculations were run through the program package ORCA 4.1.2.[12] Starting from the molecular structure obtained from single crystal X-ray diffraction analysis geometry optimizations were carried out using the PBEh-3c composite method[13] and no symmetry restrictions were imposed during the optimization. For the geometry optimization of the reduced gold complex the BP86 functional[14] and the Triple-ζ-valence basis set def2-TZVP[15] was used. For the geometry optimization of the triazolylidenes the geometry of the selenium adducts without the selenium atom was used as the starting geometry. Subsequent single-point calculations were performed on the optimized geometries using the B3LYP functional[16] and the Triple-ζ-valence basis set def2-TZVP[15]. All calculations were run with empirical van der Waals correction (D3).[17] The restricted and unrestricted DFT methods were employed for closed and open shell molecules respectively unless otherwise stated. Convergence criteria were set to default for geometry-optimization (OPT), and tight for SCF calculations (TightSCF). Relativistic effects were included with the zeroth-order regular approximation (ZORA).[18] The RIJCOSX (combination of the resolution of the identity and chain of spheres algorithms) approximation and the auxiliary basis set def2/J were used for single point calculations using the B3LYP functional.[16] The conductor-like polarizable continuum model (CPCM)[19] was used for calculation of the selenium adducts to take solvent effects of acetonitrile into account. Low-lying excitation energies were calculated with time-dependent DFT (TD-DFT). Solvent effects were taken into account with the conductor-like screening model (COSMO).[20] For all calculations spin densities were calculated according to the Löwdin population analysis.[21] In the absence of imaginary frequencies, spin densities, molecular orbitals and difference densities were visualized with the modified Avogadro 1.2.0 program with extended ORCA support.[22]

Optimized geometries of the selenium adducts

PhDippTrzSe

Se	5.71784687553354	16.82507139520392	4.11346901984289
C	4.81246706978744	15.25060884483873	3.95622962960898
N	3.48222515265410	14.98481409519862	4.21948794777629
C	5.26963582385053	14.01431819280551	3.49752057405261
N	3.12403115690637	13.74267608361430	3.95883294051984
C	2.47365539785453	15.87491435690244	4.66802937811523
C	6.60258246804398	13.59409931966580	3.04614029550135
N	4.20772762417406	13.18669861439322	3.52484714968948
C	1.24219461216508	15.86601702598171	4.02911911432921
C	2.72039510084569	16.72476200119639	5.73507460147747
C	7.47257525690262	12.97783476168216	3.95752517174611
C	6.97596207646495	13.80910461238909	1.71289216833306
C	4.19172298991631	11.78737764791543	3.15982656388675
H	1.06840360548036	15.20116180803256	3.19436705154265
C	0.24185767227469	16.71625885429133	4.47025938995852
C	1.71272092284409	17.57487285722242	6.16183880569337
H	3.68445517646486	16.72725263397787	6.22130028824413
C	7.12270162730472	12.83235644571747	5.42306235654692
C	8.71642189929558	12.55824729591987	3.50239196749381
C	8.22826306921604	13.36937741282741	1.29920810034168
C	6.07483165760422	14.51701716480825	0.72489301973911
H	4.81864356976057	11.21896890211711	3.84331317103628
H	4.57570336412296	11.66936785272079	2.14976681024656
H	3.16779345394316	11.43278745444999	3.21491483693389
H	-0.71902421875702	16.71250908217463	3.97383310766399
C	0.47619453410674	17.57442438171176	5.53405439471969
H	1.89998647036171	18.24135005589946	6.99263733895049
C	7.86226068577102	13.89382912226179	6.23975455092637
C	7.39147038773112	11.42783145056093	5.95999997251676
C	9.08926435480397	12.74530532267877	2.18297207335642
H	9.41080726020774	12.08850803481648	4.18747385794930
H	8.53988665928481	13.52488342291583	0.27368621105603
H	5.15246686892968	14.80072699681245	1.23579887576400
C	6.71746731308655	15.81444095115133	0.23574890527011
C	5.69588740238177	13.60479781830365	-0.44169266399133
H	-0.30362680569982	18.24348950019419	5.87234424012387
H	7.57793795000622	13.83656785763231	7.29188481558067
H	7.62908779016530	14.89517608051977	5.87742183179630
H	8.94308941410390	13.75357014356261	6.17796238066029
H	10.06197031957055	12.41202087973012	1.84492390196818
H	6.03458995451491	16.35066216034779	-0.42485015971608
H	6.95142553757674	16.46447638064940	1.07777793599143
H	7.63630238992277	15.62612727861565	-0.32269076100403
H	5.00381359974382	14.11173015061659	-1.11549626361338
H	6.56871784366547	13.31372605825889	-1.02829763402139
H	5.21318074607104	12.68751765303839	-0.09925420228805
H	6.05452731470392	13.02639969082833	5.54901072250318
H	8.45522956776727	11.18679724914536	5.96728309255009
H	6.88642063881603	10.66201798217988	5.36870576960865
H	7.03768839775325	11.34261866152112	6.98811735302222

PhPhTrzSe

Se	1.61289683344192	1.89925778398628	6.69706378477540
C	2.43030632599054	3.47987626300672	6.31387863825684
C	3.76110536472532	3.75856757346786	5.96714408428855
N	1.85618847851729	4.73513842855439	6.26781752929004
C	0.50163242879171	5.09442771464065	6.48943962034331
C	4.90392454490762	2.86054655477684	5.84235595322054
N	3.81590526589279	5.08959288066286	5.73703836504428
N	2.68686470295475	5.69290230195709	5.91620318335600
C	-0.15842395730224	4.64843193778378	7.62365426691713
C	-0.13361680193572	5.90684734329757	5.56234394241398
C	4.77275972666059	1.67094100003287	5.12674523347129
C	6.12847679156530	3.16139007468423	6.43850805866581
C	4.94892062662696	5.88444889175095	5.31057534521422
H	0.34588162544708	4.01179180461220	8.33519516720179
C	-1.47576281665290	5.02779410970546	7.82471773647564
H	0.39527444100133	6.23992182807863	4.68001315077282
C	-1.44906256143937	6.28343823388706	5.77826657809618
H	3.81860593501142	1.41665184408634	4.68696531210372
C	5.85432980029577	0.81681833832723	4.99296213009924
C	7.20873742549464	2.30501791092202	6.30021777631258
H	6.23414471921479	4.05210862830379	7.04532173942653
H	4.57066086955753	6.76036798610898	4.79317905439228
H	5.56902268634363	5.29575861634140	4.64107575124737
H	5.54190221834060	6.19938584018453	6.16611880557238
C	-2.12275823599389	5.84189505881183	6.90688550664203
H	-1.99730588449238	4.68223180524286	8.70676865335449
H	-1.94872786187004	6.91597917272379	5.05721083707072
H	5.74097769689886	-0.10269107524136	4.43457080353761
C	7.07453512392023	1.13174859897548	5.57363359555837
H	8.15052231409939	2.54763474405385	6.77408616526491
H	-3.15235075134510	6.13070373083602	7.07049313410496
H	7.91564292533128	0.45923407543736	5.46934009750833

DippPhTrzSe

Se	2.32416034578135	3.31573149415025	9.88243619853711
C	2.81939615103566	4.02069525328691	8.27830611353229
N	1.95619375250240	4.50820184258131	7.32773543076388
C	4.06715407237838	4.22005265357904	7.66941582380705
N	2.54091105147725	4.98167559812207	6.25310243788726
C	0.53442686196927	4.55363371928063	7.41385078273608
N	3.80379240489293	4.80395551272981	6.47478108747402
C	5.40468356765587	3.90053961469518	8.15268171100375
C	-0.18990525518978	3.45809639411729	6.94961438015889
C	-0.05722642375803	5.68774773788705	7.96553545767058
C	4.73901866080700	5.26977955679793	5.47187351564583
C	5.74848713885828	4.20235308904783	9.47047484232097
C	6.34753753425680	3.27914098740899	7.33306381984550
C	0.46932839479625	2.21411868086305	6.39854628454557
C	-1.57562085032369	3.54047543326916	7.01425695901857
C	0.74475570426247	6.84722440078091	8.51177001130823
C	-1.44578247285062	5.72147385201524	8.00858352882814
H	5.00765681731926	4.46508874104510	4.79124080335595
H	5.63512765618248	5.64105721988168	5.96026413013108
H	4.26282941518946	6.06924255847742	4.91319459481789
H	5.01375565094746	4.65474562783165	10.12169701314992
C	7.01666735370594	3.91104886333454	9.94340341028066
C	7.61612184856015	2.99219593702205	7.80980750909538
H	6.08325726458217	2.98248356824931	6.32586307423276
H	1.55196384178582	2.31637673663644	6.48937364874978
C	0.08014045978101	0.98302648204951	7.21516802258241
C	0.14957238371486	2.03849759116920	4.91442484765254
C	-2.19780008772739	4.66179392163334	7.53352656567483
H	-2.17771579372487	2.71330296542271	6.66004348705580
C	0.45503091806822	7.05738118662122	9.99708001622942
C	0.49755153665230	8.11581169491269	7.69631079915814
H	1.80748039585823	6.61204983715361	8.43338534360928
H	-1.94697629074811	6.58566867492551	8.42593098232356
H	7.27130707354727	4.15121327876028	10.96685877694919
C	7.95511132660365	3.31121289810336	9.11594377486594
H	8.33426464860018	2.50419373240576	7.16454392188988
H	0.61351615397657	0.10370901300168	6.85124982732699
H	-0.98788170685675	0.76960092066692	7.14634329416516
H	0.33619699465086	1.12395870099031	8.26462046717938
H	0.45949936425665	2.90919450314842	4.33579411185164
H	-0.91888582943278	1.89290723819347	4.74720855326346
H	0.66543864799459	1.16503088785879	4.51280849899879
H	-3.27813952962140	4.70663531655616	7.57600230438389
H	-0.58418620855956	7.33972144379767	10.17351198809092
H	1.08289929209806	7.85542176111076	10.39617107556014
H	0.66355524142001	6.14739090342310	10.55881889770219
H	0.73833158545828	7.96344706058400	6.64382726384883
H	1.11327906192559	8.93605747038827	8.06832054123244
H	-0.54391292783642	8.43613127529266	7.75457593724417
H	8.94373280307638	3.08261616873959	9.49110813226355

MesPhTrzSe

Se	3.67155145255385	9.62360908350609	1.67389256715450
C	2.19144776278878	9.74699960644991	2.72533820336061
C	1.34340255400583	8.78693020717515	3.29674205291420
N	1.62673988400229	10.92230009217199	3.15745034516140
N	0.41675581794451	9.48564469922511	3.99618564438670
C	1.38727855285465	7.33228410570392	3.21163802891683
N	0.57030009839029	10.76864039333799	3.92135207214563
C	2.08144586936027	12.24050092817355	2.86643104376633
C	-0.66281700500480	8.98004438340196	4.81843824266859
C	0.23874965308264	6.57978424439594	2.96414260139076
C	2.60773290414038	6.67323118646564	3.36070224416831
C	3.00888263047343	12.82718970346479	3.72076388605491
C	1.59541189348549	12.87347074450587	1.73136628462144
H	-1.54329966805093	8.77189319190009	4.21483898738486
H	-0.90489365084615	9.73684955812065	5.55788227871757
H	-0.34260779795609	8.06719034343789	5.31227468898907
C	0.30218184574025	5.19749277524895	2.89682799543773
H	-0.70886360367771	7.07236064332403	2.78536500565964
C	2.66678509053882	5.29181553392679	3.28964325618930
H	3.50798701441139	7.25010109995440	3.52062870705847
C	3.55971061431693	12.07543827742258	4.89340322124551
C	3.42558492548584	14.11585086573622	3.42533835100770
C	2.04219939952926	14.16443716131068	1.47635390315998
C	0.65317365781403	12.17462689279857	0.79997535402409
H	-0.59628446490664	4.62852531166171	2.69766213665496
C	1.51656561393706	4.54951766831446	3.06342688517138
H	3.61948975788156	4.79320150683442	3.40824066567729
H	2.77216100950795	11.72933299766105	5.56415536101182
H	4.23588945837933	12.70112092935504	5.47349264616709
H	4.11920395551344	11.20093162167728	4.55455862848147
H	4.14873205527883	14.59453425929103	4.07630498452904
C	2.95084570469745	14.80015608139666	2.31089303789526
H	1.67649157999155	14.67982015010663	0.59578089627532
H	0.34283854000905	12.83588947488170	-0.00718793162552
H	-0.24757135331163	11.83159866517892	1.31092589865284
H	1.13627621357375	11.30322354579149	0.35265575719957
H	1.56830013906370	3.47028315998044	3.00553787779714
C	3.42614572572819	16.19357717310166	2.02102276641016
H	4.51033873824859	16.22383834249375	1.90509614817643
H	3.16863785535557	16.87650813120896	2.83195814117470
H	2.98581957566898	16.58698525990604	1.10606113476689

MesFcTrzSe

Se	8.32064792815312	6.79110940899316	2.66366922217549
C	6.92159966555343	5.71126137477874	3.12327181599179
N	6.93234403658948	4.85575395989721	4.19452489578853
C	5.65241642741194	5.48153595104927	2.57078824057557
N	5.84312548273435	4.13861816479025	4.32891212305723
C	7.99114909616500	4.68030746684364	5.13165541915603
N	5.09204730479347	4.51952995253849	3.34261649601587
C	5.01416814402568	6.05353592776206	1.40248687945029
C	8.94958911648816	3.70665647210866	4.87619264905639
C	8.02652758092615	5.49875926026482	6.25228095507493
C	3.80928566904842	3.86795221468765	3.18765420170021
Fe	4.61736016897481	8.03269536260913	1.05930512351308
C	3.61537321066261	6.26233185424051	1.18712581833614
C	5.69698363877703	6.48747668217753	0.22964696742410
C	8.90545729577361	2.89251370744010	3.61964390198156
C	9.95949326956221	3.54140200006097	5.81251063666026
C	9.05684489537780	5.29660902202272	7.16134429948937
C	7.01027435572593	6.58089898975428	6.45175264151667
H	3.63015651017431	3.66295355052287	2.13607809318645
H	3.00934895169254	4.49447507110640	3.57592012911802
H	3.83978328639562	2.93794621153448	3.74620207179549
C	4.73191276712997	6.94649231351525	-0.69206726061579
C	3.44921360547982	6.80382427276561	-0.10825079247331
C	5.29274262896878	9.10641443990226	2.67763754668367
C	5.93759645387770	9.54632237333874	1.49721074897830
C	4.94250732498348	10.01028080617763	0.60244106251845
C	3.68015163378884	9.85269434926007	1.22795440827403
C	3.89822073117682	9.29469722604279	2.51356596432054
H	2.81414942365467	6.06755156529548	1.88435615324312
H	6.76878064465328	6.48133674351182	0.10149781571318
H	9.01961664742765	3.53669219433679	2.74516817996744
H	7.96361990346067	2.35213151161576	3.51540749594472
H	9.71048375736678	2.15994890652600	3.60276736522239
H	10.71773591366168	2.78659918710856	5.63686259894166
C	10.02836750442716	4.32521666774215	6.95896024525652
H	9.10411948886359	5.92139904425036	8.04589155160651
H	7.10954237294626	7.34037252645185	5.67274972687337
H	7.14653434244157	7.07046334557862	7.41447498849846
H	5.98995813585731	6.19672451135871	6.41675223351426
H	4.94052194668403	7.34682509018386	-1.67293621958943
H	2.50794873530348	7.07815492768002	-0.56065695029985
H	5.79244311324544	8.67740143933478	3.53378655375002
H	7.00002812890400	9.49455437634991	1.31442178328273
H	5.11312914703272	10.40286755437600	-0.38930439746867
H	2.72221118236775	10.11121323361359	0.80076513554629
H	3.13325170273922	9.05448764489076	3.23756654477230
C	11.15250933797733	4.14506551599541	7.93634087598234
H	12.06101794545113	4.63130817938018	7.57612440775999
H	11.38743936185760	3.09157126642286	8.08790506781693
H	10.91483008326517	4.57669618181089	8.90769458491612

$PhCF_3PhTrzSe$

Se	20.97888488962355	3.35217598657207	4.16081483768076
C	20.66698229598793	4.16297715358693	5.75662616052019
N	19.43359221715908	4.37347953108567	6.33431848150910
C	21.52708261947866	4.72329266867479	6.71288815416297
N	19.47143651556800	4.99217008840163	7.49375244733273
C	18.17242063270369	4.03689402485456	5.78312466716767
N	20.72974452566367	5.19763674464364	7.69838033304394
C	22.98159766161412	4.82253156854362	6.71317828314502
C	17.88619719363276	2.69504402131050	5.60162875593042
C	17.25851145172307	5.02140708621404	5.41675310216038
C	21.11008949834490	5.91565570971206	8.89800713530199
C	23.73377769504486	4.47998757779388	7.83685270374216
C	23.64059101426314	5.25378023513670	5.56227244670998
H	18.62919156622896	1.95532257432734	5.86296725913878
C	16.66183670714814	2.31734741700675	5.08047163617327
C	17.54851800757081	6.49047006900259	5.56977200403130
C	16.02919987185445	4.62632615208013	4.90260013000404
H	21.97495323071276	6.53639870213319	8.68373709093159
H	21.35307081342479	5.22301370612584	9.70045253265495
H	20.27181122774250	6.53689255672825	9.19684441591355
C	25.11450741338907	4.59216082916289	7.82101612910166
H	23.24584498672953	4.08938991632983	8.72117015086859
C	25.02083122148004	5.36166414031981	5.55013919229033
H	23.06651502692886	5.49454863171409	4.67844755735013
C	15.73161762946118	3.28446042147041	4.73752834016106
H	16.43932082920781	1.26868529221805	4.94001743114245
F	16.77163039177920	7.22163666999549	4.76592821311510
F	18.81259433816899	6.79470409230567	5.27134071129131
F	17.31760173443766	6.91259509687837	6.81731540351510
H	15.29820732474282	5.36768549328226	4.61416780995802
H	25.68546829977350	4.31678676610976	8.69770076081550
C	25.76106071107769	5.03688645031278	6.67790308432675
H	25.52064614024343	5.69900932568184	4.65209995705498
H	14.77203545814920	2.99730879464183	4.33006177198365
H	26.83967885894120	5.12018450564273	6.66251090977059

MesPh1,5TrzSe

Se	3.33727345780641	5.99816611368488	13.45550040457218
C	5.03935578135381	5.59255432020941	13.97090781783957
N	5.35709057307786	4.58275612350427	14.84168150838017
C	6.30058744472041	6.11547812002368	13.64917831562749
N	6.63425121748441	4.45357770876122	15.08525522602237
C	4.41329100567701	3.69836354589491	15.47904629594827
N	7.19120054359813	5.37874994020178	14.36380924132077
C	6.66312135352817	7.21633395155782	12.76489109101799
H	3.84317090163297	3.16727251568984	14.71995052008609
H	3.71558316655191	4.27995722021648	16.07761633356348
H	4.96609648572198	3.00206627530234	16.10210239392667
C	8.60974664490563	5.52093771693974	14.43260131778156
C	7.78385472919417	7.15898327312095	11.93464884729063
C	5.86754642162503	8.36337854427380	12.74685008877266
C	9.40041333866742	4.56217971845954	13.80677612341000
C	9.13951412984102	6.61620922226279	15.10823279242080
C	8.11153768804461	8.23139742569912	11.12243022308590
H	8.40000530136188	6.27103298317067	11.90272815704665
C	6.19967046832370	9.43144631604332	11.93039703943828
H	4.98440904688673	8.41013074377127	13.36866237881736
C	8.79862071690756	3.39204905366504	13.08677827786103
C	10.77759321402219	4.72902861845808	13.86931152819744
C	10.52209726223046	6.73710760609947	15.13849818796910
C	8.26490951768754	7.63744992896169	15.76975581481497
H	8.98146214112200	8.16940969950155	10.48231264480291
C	7.32304924565286	9.37227452733205	11.11920503872091
H	5.57341454207769	10.31354332382316	11.92794340089176
H	9.55227209475570	2.87594149398313	12.49502500860424
H	7.99659701237637	3.69188502610903	12.41002495743622
H	8.37442270222367	2.67170712172026	13.78662430129249
H	11.41466880803629	3.99816326806851	13.38498231266540
C	11.35505983197066	5.80796643030721	14.52696846200809
H	10.95889785556041	7.58316272721563	15.65658371109403
H	7.80604321098384	8.30140614169397	15.03492638461448
H	8.84574335121462	8.25454841407682	16.45292255208127
H	7.45911675622689	7.17848445928654	16.34279196415688
H	7.57866319506641	10.20768637815983	10.48084081203389
C	12.84576234087128	5.95702252313370	14.60150185560129
H	13.34868845608415	5.36452071525442	13.83864567738424
H	13.22128394525875	5.62687486838363	15.57175995102504
H	13.15120409966735	6.99513589597844	14.47283104037539

CyPhTrzSe

Se	-0.36279151455085	6.21936759138830	12.82890941582803
C	0.25770632688953	5.64915574717979	11.20743880149578
N	0.75703525718374	4.40004090671159	10.93271074090144
C	0.37591509656510	6.29546586370112	9.97049059606524
N	1.15089960555863	4.24058063005861	9.69738516881360
C	0.92721901098638	3.31747264820405	11.89468047818643
N	0.91177221764951	5.37928529843050	9.12849491512020
C	0.02016474884918	7.65621505039086	9.58699003713912
H	0.12348869906677	3.47026000392150	12.62072925667943
C	0.77670462127286	1.94702183861244	11.24779268442225
C	2.26373568339222	3.46184466397857	12.61691935221943
C	1.19764743367571	5.50722480728311	7.71544859718680
C	-1.20610203575969	8.18492105792361	9.98982695590117
C	0.88657619502057	8.45296161564261	8.83800057005840
H	1.54217674597275	1.80411213197827	10.48058398040707
H	-0.19268488097557	1.87000512543827	10.75022163218169
C	0.91618196325521	0.86367878019439	12.31419722793219
C	2.41246680893912	2.36710658217449	13.66760279998790
H	2.31889679716854	4.44823260494222	13.08146877247341
H	3.07544379859252	3.39311089919118	11.88462906127540
H	1.15705844808537	4.51734120044837	7.27204171058977
H	2.18634639651852	5.93228265923370	7.55688245735239
H	0.45357978892895	6.14957225888586	7.25358506219156
H	-1.87026375424699	7.58537564584128	10.59646662447966
C	-1.56268737274846	9.47243980289831	9.62665299015000
C	0.52445772880085	9.73978443043319	8.47434011623504
H	1.86699132654861	8.08373628371425	8.56470453996537
H	0.08880429363143	0.94453625209376	13.02664493153358
H	0.82530606608451	-0.11987816596287	11.84887022630992
C	2.24193834056489	0.97963526160794	13.05912554380956
H	3.38561084795462	2.45449691309779	14.15465332837205
H	1.66302269427580	2.51986337780669	14.45073521631861
H	-2.51694594399486	9.87031941270542	9.94453966694269
C	-0.70359604747119	10.25151375982416	8.86486027961282
H	1.20980031265096	10.34793678310590	7.89926811328046
H	2.30487672136658	0.21480339988473	13.83620168645743
H	3.06563421077088	0.78292215191974	12.36454272698402
H	-0.98544663647270	11.25808472511629	8.58600373514013

BnPhTrzSe

Se	2.73108556508568	1.19986836796365	6.82815566967262
C	3.50642414893965	2.85511758326707	6.80181620424617
N	2.96281687825059	4.01210511420684	7.29895219009014
C	4.74156994711061	3.29836081903550	6.31106506825991
N	3.72418467032759	5.06648868493306	7.16829198765159
C	1.64628639441043	4.17904081609283	7.89494052600474
N	4.78811264714090	4.62539179477076	6.57806436248484
C	5.80609996841329	2.55634579746496	5.64592096204282
H	1.76188955432049	4.83377850053540	8.75825647876513
H	1.34668141853410	3.19098892171888	8.24362879109256
C	0.65945968193404	4.74400806091766	6.91116809582719
C	5.86314768878918	5.56073860282043	6.32200351048858
C	6.40677505708999	3.03373416983245	4.48098836455725
C	6.22542595198335	1.33497896173281	6.17242750906188
C	0.04492930323322	3.91485606808312	5.97742545693199
C	0.36987906796914	6.10310858878525	6.91212981866911
H	5.78807725086880	5.96761933053867	5.31608894188675
H	6.81670734070904	5.05316424928454	6.43414512795484
H	5.78651136956144	6.36854801591648	7.04287528180977
C	7.42491009159630	2.31987374867342	3.87010975318186
H	6.05651998107518	3.95045117130542	4.02345050705438
C	7.24131575983182	0.62356223558867	5.55696375397178
H	5.74444169760987	0.94338984240189	7.05793033286771
H	0.27768989049755	2.85654631014576	5.96967990549271
C	-0.84891113512128	4.44481728566520	5.06006296836884
H	0.84805045657690	6.75397394969231	7.63442294575219
C	-0.52795900373818	6.63178394204934	5.99669268569984
C	7.84727928485373	1.11427992993481	4.40924822542620
H	7.87656777961974	2.69903988203224	2.96314408501603
H	7.55848319828355	-0.32199742878872	5.97576160158620
H	-1.32290644153256	3.79375980180808	4.33748524684590
C	-1.13828041168749	5.80213283447309	5.06837281724529
H	-0.75092670303266	7.69053433538923	6.00946840527198
H	8.63845756082766	0.55317336995023	3.92998209879296
H	-1.84015591033168	6.21182634177864	4.35386031992823

bicaSe$_2$

Se	4.12625616552090	19.66379098908636	6.62378303801536
C	5.48625412070164	20.47218831164079	5.70778613725697
N	6.71661394350309	19.93493911612503	5.42654992903498
C	5.58839600169878	21.74294593601488	5.13627832475004
N	7.51532024331580	20.74162487439609	4.77790738349691
C	7.22235799498625	18.63025033150394	5.83053038807925
N	6.83295802776241	21.81802224411621	4.60158541802693
C	4.64875483927057	22.83427386843843	5.13627128461179
H	7.73366226555176	18.19928183045273	4.97022102634356
H	6.34333914449713	18.02479374463839	6.05026512526524
C	8.13977517982970	18.72998649125678	7.01678596303483
C	7.42667018802503	22.93099335471415	3.88964591996508
N	3.40418460870601	22.75915495502111	4.60160884755922
C	4.75085554407688	24.10502753815646	5.70779929351478
C	7.61711701191158	18.88529395710929	8.29717828735259
C	9.51724921322108	18.68902028687771	6.84141444082513
H	8.38865330150283	22.60731476272433	3.50652498101052
H	7.54365628498411	23.77321938083723	4.57142265464256
H	6.77736475666734	23.21996568851159	3.06649407508689
N	2.72180290927161	23.83554417101211	4.77790364753366
C	2.81048130406388	21.64613452523983	3.88973982037580
Se	6.11079464226349	24.91344102067181	6.62387051007318
N	3.52048982193409	24.64225219308589	5.42654085187840
H	6.54320278661246	18.92616649247796	8.43600709166563
C	8.46786418608412	18.99710928222330	9.38523582726013
C	10.36788490410589	18.79555267047362	7.93162774227567
H	9.92997798544684	18.57377270705251	5.84624857996763
H	1.84847833648440	21.96976837425872	3.50663134376800
H	2.69353819950914	20.80393788766363	4.57156079633557
H	3.45977120892014	21.35714021987594	3.06658357989206
C	3.01471211049095	25.94692761445463	5.83052100739241
H	8.05451166094188	19.12050012204289	10.37735447987921
C	9.84321593394890	18.95082760116598	9.20533683036463
H	11.43930053247535	18.75930557407400	7.78530454128405
H	2.50339617958726	26.37788408130488	4.97021256305734
H	3.89371589401836	26.55240609925388	6.05025568869174
C	2.09730058893254	25.84716615380538	7.01677831106702
H	10.50475177718297	19.03611443791685	10.05735489807879
C	2.61996732710602	25.69186494406512	8.29716777997871
C	0.71982504646891	25.88810115721765	6.84141191682029
H	3.69388313596544	25.65101622999682	8.43599214166519
C	1.76922705656422	25.58002552765624	9.38522815084243
C	-0.13080395433215	25.78154474355899	7.93162809336709
H	0.30708988615554	26.00334333147045	5.84624808053348
H	2.18258621670459	25.45663982315546	10.37734467649648
C	0.39387353562658	25.62627657401910	9.20533454494487
H	-1.20222097942073	25.81776781036141	7.78530919031717
H	-0.26765706884565	25.54097096882337	10.05735479632076

Optimized geometries of the triazolylidenes

PhPhTrz

C	2.28367327645642	9.72638137325728	3.00996542051660
C	1.27553307023069	8.79776681793587	3.24888377725142
N	1.54858686358961	10.87356887597560	3.05562731436796
N	0.10210074222735	9.47312264088344	3.42035333653846
C	1.41656441064728	7.34630101032062	3.33406160108652
N	0.26052998663679	10.75191606282030	3.29854224364305
C	2.06810587607171	12.18235411396273	2.86875048870351
C	-1.21711361851614	8.98358346765278	3.75612496025648
C	0.45500263103343	6.44798176573259	2.87198878954755
C	2.59902050550935	6.83717355317755	3.87386984750289
C	3.42079436094520	12.32320451512756	2.59479987065213
C	1.24479996799175	13.29696059781536	2.95799628592206
H	-1.76613935510761	8.69847131372619	2.86043910409637
H	-1.75796000119356	9.77300812689186	4.26872508799128
H	-1.12584513700535	8.12042152768809	4.40961958237027
C	0.65614443506325	5.07975084841808	2.97626533788836
H	-0.44513778132236	6.80581567611160	2.38929891323394
C	2.79991283381729	5.47218557063156	3.97252344610952
H	3.35749130069391	7.53357716178435	4.20540068889154
C	3.94875416971266	13.59026469272388	2.41163859147143
C	1.78738338964157	14.55844942482648	2.77174600448752
H	-0.09962138547286	4.39883726853246	2.60737867458108
C	1.82560322976819	4.58637000058026	3.53199946011495
H	3.72191613306568	5.09662278206883	4.39642345481809
H	5.00382726852191	13.69791919382818	2.19806515970496
C	3.13795006200776	14.71242809719369	2.49866897890778
H	1.14496646323813	15.42612588805330	2.84158688370070
H	1.98240735621097	3.51887593906672	3.61196434204569
H	4.03440537622479	11.43559746979498	2.53006861874405
H	0.19157560583176	13.18521514016916	3.17015811950623
H	3.55522646026444	15.69993811350709	2.35414187513696

PhCF$_3$PhTrz

C	20.70156798306056	4.19154509822174	5.76853862644142
N	19.48616476277488	4.52389745490699	6.28812837114420
C	21.52116833908816	4.76806410416083	6.73604977834254
N	19.47097112437216	5.19914335067223	7.41868418781234
C	18.24364931336507	4.15365825044432	5.71604764960239
N	20.72659974579050	5.35670214623780	7.67812946942911
C	22.98152345676592	4.77960160955062	6.75843602726519
C	18.13719884391731	2.84341135674667	5.27437383595296
C	17.17114480033313	5.03593034864196	5.57509983138096
C	21.08026624100119	6.14143187904120	8.84235853505972
C	23.73675662762052	4.68443654167795	7.92705120145616
C	23.65126361813973	4.84937490594560	5.53549496655461
H	19.00392415730764	2.20327121823253	5.35975706066312
C	16.95467156041215	2.38391158785433	4.72646304459078
C	17.23791525898514	6.49576699447887	5.94381200992937
C	15.98358803908498	4.55163055552110	5.03366861403135
H	21.98331561632050	6.70773021905931	8.63264126250023
H	21.24983282335265	5.50079365659262	9.70565037901022
H	20.26329850644354	6.82298194657937	9.05749239852259
C	25.12266400453484	4.69080382934962	7.87652270981229
H	23.25205907135930	4.56533968798955	8.88734707065813
C	25.03364029967583	4.85017131440537	5.48707360234074
H	23.06560349627302	4.89553114002784	4.62717517616945
C	15.86856736256130	3.23677769304773	4.61974146283829
H	16.88300327464294	1.35894507065917	4.38898584683299
F	16.30338124534771	7.19492834161047	5.28703952412197
F	18.41202194331584	7.04595248996128	5.62938708887046
F	17.01975387967834	6.70464242299485	7.24389484979116
H	15.13710569003283	5.21208795040457	4.91501448542388
H	25.69136698025401	4.61168250349731	8.79369303712209
C	25.77641083448323	4.77948984664600	6.65806896424469
H	25.53503042267916	4.90789657155330	4.52995996094236
H	14.93511607043349	2.88726802403257	4.20063833298363
H	26.85761971696894	4.78353390268226	6.61955580047786

MesPhTrz

C	2.23346578598136	9.73791146614478	2.85520752891873
C	1.28737574435256	8.81795692006076	3.30802614769785
N	1.57187882071722	10.89206581880063	3.13866420512943
N	0.22074426989571	9.50952889751525	3.80540027741567
C	1.39879104113807	7.36172279271763	3.28915454298748
N	0.38618563145235	10.79060502194377	3.70438351318853
C	2.05751608572780	12.20465054294294	2.87004624234880
C	-0.98232937443342	9.03422213899463	4.45361937001814
C	0.31981724039508	6.50209650713694	3.08287527412472
C	2.67024121197182	6.80629868017185	3.44762668687729
C	3.10438992426096	12.70015455787792	3.64344627741333
C	1.48949255258830	12.93356234554206	1.83291272646699
H	-1.74881095313399	8.78834344073548	3.72080514868952
H	-1.35364750557084	9.81820856849658	5.10633464329603
H	-0.75238681013582	8.14926156031784	5.04070402010636
C	0.50155209996315	5.12714001165264	3.06960197972853
H	-0.66930643408922	6.89681489642795	2.89122057587742
C	2.85012538805235	5.43519602992536	3.42908522338152
H	3.51206955331555	7.47354533162493	3.57523885140270
C	3.71597160829154	11.87862630499886	4.73715922493478
C	3.57010586080782	13.97539890316717	3.35888289914570
C	1.98773012073881	14.20866396491225	1.58738651807092
C	0.38468732678497	12.36538491921391	0.99286926229888
H	-0.34775744100526	4.47743091796378	2.90356538214063
C	1.76500432668367	4.58761177764986	3.24880571116781
H	3.84315308690510	5.02451421202286	3.55622294492697
H	2.97287988378074	11.57863135130024	5.47785821216894
H	4.49362653562648	12.43814736088964	5.25456562570781
H	4.15421778637671	10.96494434278204	4.33482923989012
H	4.38459494098952	14.37780047479441	3.95108484007257
C	3.02284342786445	14.74593625211293	2.33845464014111
H	1.55803344945949	14.79072725938320	0.78009233463033
H	0.20352757605012	12.98964443292459	0.11944298324618
H	-0.54932741067116	12.29662339132596	1.55160234693394
H	0.62922293507801	11.36227277355904	0.64053046537835
H	1.90588688482798	3.51497519694473	3.23718577977618
C	3.55076484723223	16.12326520398025	2.06112576527487
H	4.61794537762035	16.10122890186730	1.83618662904712
H	3.41627763247839	16.77984678100448	2.92214922857933
H	3.04517697163107	16.58456974817259	1.21393673139837

MesPh1,5Trz

C	5.04764410395800	5.71810271393130	13.95188387798613
N	5.34607262006904	4.73034994086168	14.83324747924820
C	6.33830752179175	6.15404581243487	13.64653350267940
N	6.61359882302827	4.51515073203583	15.10275625729220
C	4.37854921352185	3.86761224496638	15.47270160476568
N	7.22124267668522	5.39992323932763	14.36842883662863
C	6.69941639389411	7.24294643774621	12.74104493743639
H	4.34430796310498	2.89844388977442	14.97743667725186
H	3.40996033778601	4.34653850145958	15.38646863133668
H	4.63401502083009	3.72335468521060	16.51988935304987
C	8.64022231441907	5.48631498181089	14.46090365329221
C	7.92786620392920	7.35969514106847	12.08831318530541
C	5.72552803701945	8.21710577355460	12.50289280862833
C	9.41051811597358	4.52709207774769	13.81161116490908
C	9.19933207661646	6.54589245785600	15.17028103470818
C	8.17806004897998	8.43122998130047	11.24412914455576
H	8.69881984438236	6.61332413946102	12.21619963406898
C	5.97862562750561	9.28170323593202	11.65749405194698
H	4.76519688387704	8.11330865236243	12.99013195525630
C	8.77499527377196	3.40228376838296	13.05086309996745
C	10.79223791629586	4.65345900316915	13.88516009156071
C	10.58404660747565	6.62914570286203	15.21223575824061
C	8.34290427925320	7.57179485396988	15.84860002835026
H	9.13689128173518	8.50165971839422	10.74739896995889
C	7.21016461775459	9.39860623931401	11.02723854684826
H	5.21024880220352	10.02515153545374	11.49005927443081
H	8.03482467160491	3.76399351909862	12.33515416906220
H	8.26207753465307	2.71211652595991	13.72107637896501
H	9.52326381611917	2.83792771781249	12.49730145754756
H	11.41174715353631	3.91917510016576	13.38326457236728
C	11.39547380120730	5.69582318518124	14.57708958345900
H	11.04101638104951	7.44756529831323	15.75713124952306
H	7.87157198987348	8.23705491287721	15.12284087244604
H	8.93880996812261	8.18670854042268	16.52090962230812
H	7.54621048645765	7.11304505812674	16.43428150691089
H	7.41023203473244	10.23281984004215	10.36778435720754
C	12.88946829088970	5.80775152232457	14.65881750864073
H	13.38250318260486	5.14450626986418	13.94961540943113
H	13.24727773758336	5.54533208242879	15.65594433103709
H	13.22558034570364	6.82410496699430	14.45253542139104

DippPhTrz

C	4.54047508087589	15.23368163188515	3.61960288123317
N	3.48152206215530	14.90123569388273	4.40460502895183
C	5.22471434041636	14.01720271334212	3.63536288076470
N	3.43337639275328	13.67032971069516	4.87249499865552
C	2.41342117692018	15.77592609938791	4.76900751370755
C	6.47795455351837	13.70590982180716	2.95263936032452
N	4.51013837260606	13.13319214406320	4.38984501555495
C	1.34043229754952	15.91581562961188	3.88822265417008
C	2.49928638140346	16.45852969836891	5.98117752136593
C	7.45276554797158	12.85346954313388	3.47086490202404
C	6.72421726842939	14.33287123563763	1.72949894113281
C	4.74717000352333	11.73226711223454	4.66414234968965
C	0.31527628454337	16.77565117075044	4.26469436172021
C	1.44305389343467	17.29777331890909	6.31866625806769
C	8.62504422855302	12.60942189816689	2.77095873568695
C	7.89596087676460	14.09181427577105	1.03543761477129
H	5.20436793516994	11.26603868634920	3.79574004118080
H	3.79388480445481	11.25719527315388	4.87395018479651
H	5.40322246689541	11.60991066529455	5.52401695486436
H	-0.53482319701001	16.91828477027844	3.61091307138735
C	0.36258113686054	17.45501872199323	5.46975355395686
H	1.46532812481281	17.84030081965174	7.25478909779161
C	8.84903926464115	13.22194668783487	1.54849619744942
H	9.37108435232876	11.94847831825692	3.19228431476685
H	8.06714977722175	14.58538000835192	0.08783509579078
H	-0.44849146687598	18.11584328175322	5.74685327281861
H	9.76424216388343	13.03194475831551	1.00369604313178
C	3.67258399933770	16.27722033391914	6.91894293883757
C	1.31548117794671	15.21264350859976	2.54876606322576
C	3.32417481956662	15.26643932051339	8.01302314452048
C	4.15061726478678	17.59634966483096	7.52131611070242
H	3.41858724900715	18.03268808120389	8.20264949417089
H	4.37011529684205	18.32997324423071	6.74611035617380
H	5.06324410090099	17.43403944077699	8.09606658241310
H	3.03004479829805	14.30765010393350	7.58678056534992
H	2.49710982797945	15.62540550702308	8.62846205460881
H	4.17898030938350	15.09781607662208	8.67046179228367
H	4.51144181398760	15.87891231304036	6.34435437676077
C	-0.08194088932003	14.75822464613628	2.13621694862050
H	1.93056545928840	14.31273943629714	2.62116683817713
C	1.94043553330227	16.11394410772448	1.48114146593653
H	-0.55854949104474	14.15875789556937	2.91229506793321
H	-0.02183532975240	14.14995919722894	1.23303632899761
H	-0.74015319727635	15.59776381388424	1.90748594796853
H	1.36112515359250	17.03150835490157	1.36157996388104
H	1.96518179736346	15.60574972570469	0.51525853557298
H	2.96070810771793	16.38489730964258	1.75238054566148
H	5.98194088001621	15.01768887750146	1.34179878109145
H	7.32636719427513	12.39995535183408	4.44527725135653

BnPhTrz

C	3.54303550155036	3.03902763966812	6.83797246623939
N	3.10415626182328	4.22000067866708	7.34484001631997
C	4.76714809987735	3.45843132858920	6.31665544039325
N	3.88179092657664	5.26873451985655	7.20821140823747
C	1.80336849388280	4.42787560287303	7.95273968025502
N	4.91015783711229	4.78997456031755	6.58026739560894
C	5.75917826138731	2.63642198712502	5.62902622832948
H	1.89784392436611	5.19344686915637	8.72279971576281
H	1.54483528181847	3.48664698381312	8.43405796826443
C	0.76016114720884	4.81627028523762	6.93850505333921
C	6.02221019097259	5.67908682753083	6.32447190294277
C	6.55450397979376	3.09631470077031	4.57954540043656
C	5.88441741600421	1.30248313743009	6.02276911379433
C	0.45503830075250	3.95609495922776	5.88700229254839
C	0.08778208639873	6.02595943044408	7.04267720820036
H	5.95970184387968	6.10222954674896	5.32349468587871
H	6.95528403591878	5.13107418223192	6.42405162100803
H	5.99367454702714	6.48417728330033	7.05226022796195
C	7.47154045910082	2.25680987330403	3.96463466773788
H	6.44009423183575	4.10440606140861	4.20272181788867
C	6.79569953888921	0.46548216853412	5.40483754835616
H	5.24657685234379	0.93703100248155	6.81658026791495
H	0.98970605000507	3.01730381025399	5.79742084814507
C	-0.51112574011597	4.30625484018201	4.95874083091229
H	0.32473991285215	6.70487034785366	7.85351848516399
C	-0.88420255946175	6.37543339283627	6.11489918147727
C	7.60073353070554	0.94078795954500	4.37799719713545
H	8.07597313689966	2.63213611781710	3.14906611336544
H	6.87943066100018	-0.56448642216988	5.72648499603524
H	-0.74202398036595	3.63192005555065	4.14427412622196
C	-1.18483818062679	5.51571443050734	5.07113559720819
H	-1.40200361390588	7.32128659202732	6.20740582210302
H	8.31575547493441	0.28624193462205	3.89715427373069
H	-1.94108391044108	5.78569731225821	4.34547040108262

PhDippTrz

C	4.82931818301420	15.15713114475348	3.94908329396605
N	3.49092611609750	14.91436986168209	4.09834071063607
C	5.22577531090661	13.93163973380275	3.43373828393730
N	3.05396806833360	13.72322181719793	3.74432792793880
C	2.54657235753236	15.84450213974959	4.60692669380592
C	6.55535387300418	13.44753255388477	3.01605551514123
N	4.12867773504308	13.13004556407933	3.34047857807574
C	1.20196271044164	15.51305424140671	4.71178539481995
C	2.99725627386429	17.09715246365830	4.99804829281488
C	7.39086971386648	12.82907644970890	3.95765441388618
C	6.96113563593841	13.61612076818866	1.68437432604337
C	4.05856846621622	11.77905524175362	2.83314337882056
H	0.85240520930609	14.53763673797528	4.40645763664585
C	0.30925390389990	16.44693195632038	5.21286365006665
C	2.09309904384913	18.02000418946253	5.49663576067958
H	4.04977687342540	17.32439272111957	4.90320289303364
C	6.97467181791180	12.72351425753762	5.41010318550891
C	8.63809125275595	12.37688387735155	3.54152085444175
C	8.21614565907563	13.15008055551308	1.30907203736327
C	6.08592291743223	14.36043382020688	0.69740434760930
H	4.85896698870989	11.18578381081369	3.26795900875604
H	4.17332183223000	11.77840295125723	1.75044336822051
H	3.09620933942940	11.35395382583639	3.09912854321793
H	-0.73783740074837	16.18707961931942	5.29387824037606
C	0.74753266769996	17.70192028555850	5.60714042956369
H	2.44699421486114	18.99600748254902	5.80096519919289
C	7.35090123715977	14.01186277936425	6.14602477657532
C	7.54129329307630	11.50087051135753	6.12603746304806
C	9.04561343978596	12.53301623816538	2.22852509075169
H	9.30434631890894	11.89788734132930	4.24662576404263
H	8.55640520347404	13.26900729081288	0.28895210306025
H	5.04052573278771	14.19730703458847	0.97532762350744
C	6.35030816046466	15.86371854278327	0.81032624697829
C	6.24125010878761	13.88485092762380	-0.74417948547748
H	0.04543975969218	18.42642066975448	5.99741626527578
H	7.03171553235047	13.96779506822618	7.18900348657785
H	6.87939085077702	14.87920883764872	5.68379883898179
H	8.43214306183568	14.16139377737424	6.13134665427489
H	10.01932547025608	12.17424384731202	1.92031136793810
H	5.70685718607866	16.42117795303268	0.12711845550475
H	6.15975678043628	16.22038464251223	1.82265521604294
H	7.38730039327345	16.09198206423345	0.55708200700863
H	5.49144811671270	14.36265019940380	-1.37551276724859
H	7.21554064818368	14.14322838029443	-1.16183576763098
H	6.11483100077175	12.80499650783428	-0.83427726916530
H	5.88473916562359	12.63829037641657	5.44814655539968
H	8.62059158017112	11.56995188792533	6.27030771026277
H	7.33475313457527	10.57690706697786	5.58401415037425
H	7.09613253306976	11.41242924259734	7.11751321743962

MesFcTrz

C	5.80057470559326	-3.76009500876448	5.43290054476082
N	7.02830535895965	-3.24843524667756	5.13568221193309
C	5.00246505297633	-2.75473280910805	4.89084797237413
N	7.07018149728349	-2.09084563327759	4.50614998411914
C	8.27130015703516	-3.87603468296047	5.43605629288634
N	5.81720908740839	-1.79667532005595	4.36724480330544
C	3.55241176647790	-2.61436090118183	4.86926147994718
C	8.69524179949212	-3.92068370738386	6.75865650375043
C	9.00560875160717	-4.44668687715890	4.39963468776503
C	5.45620583774445	-0.55994838805073	3.71143873190026
Fe	2.21104047411668	-4.13428380921042	4.63222739776406
C	2.70647840297203	-2.70087225211943	6.00942995108122
C	2.72242487278595	-2.36583556752805	3.73775406846207
C	9.90531655883944	-4.54957688565166	7.02965669113004
C	7.87332226063666	-3.32232495462295	7.85936419489457
C	8.51057734275560	-4.41290034236204	2.98430873533774
C	10.20835200516254	-5.06088732348107	4.71848918430615
H	6.28263369957609	0.13798272937279	3.80295860843876
H	4.57034321032656	-0.14754846468193	4.18618179987235
H	5.24467771789396	-0.73016081251901	2.65732368121198
C	1.38015161851135	-2.30090732952816	4.18194100929381
C	1.07333743542290	-5.80091555718030	5.02984574340491
C	1.37127252040411	-2.51178760390494	5.58349765413276
C	2.37799724045755	-5.95701061710909	5.56219682801354
C	3.30391763452125	-5.87020988889616	4.49508610948035
C	2.57232031039211	-5.65704702815816	3.30125484938380
C	1.19296847974003	-5.61325585674623	3.63027021210234
H	3.04469829356468	-2.89246368095976	7.01684279183947
H	3.05614162518418	-2.26714552068663	2.71434755275246
H	10.25223003058568	-4.59214754920473	8.05579431843613
C	10.67549687563061	-5.12090000786504	6.02656636954535
H	8.38172047178717	-3.41487418483101	8.81776253015962
H	6.90410824955094	-3.81651254499660	7.93024991738771
H	7.68046369541003	-2.26245728375876	7.68578458659263
H	9.10881099317271	-5.06513803294254	2.35020564024037
H	8.55823703214953	-3.40689309916455	2.56639005617338
H	7.47307597349796	-4.74414217615942	2.91849228085891
H	10.79195205146459	-5.51218655051646	3.92353922739922
H	0.51389000999207	-2.13667495412269	3.55844316925070
H	0.15067578219748	-5.81380236759164	5.59134264248233
H	0.49616609576060	-2.53062878285321	6.21596277327451
H	2.63032775205160	-6.10021016446398	6.60229518551278
H	4.37945902839993	-5.88546760333745	4.60245419737160
H	2.99158336566366	-5.53792494914140	2.31288327696708
H	0.37784543601595	-5.46134856616357	2.93794907941908
C	11.97660173144495	-5.80123900434806	6.33784859357760
H	12.23049238823059	-5.71914951259094	7.39372457012165
H	12.79866544976481	-5.36858169008588	5.76609538134220
H	11.93617354689496	-6.86336191720632	6.09125641577425

CyPhTrz

C	0.24562110570106	5.68056236366283	11.18282406758257
N	0.93765794133124	4.52877354069251	10.98324538301794
C	0.50330000323643	6.31478214880612	9.96597568639968
N	1.55381329384404	4.38397239561870	9.83268457066656
C	1.05311120613164	3.44798299797799	11.95240368427363
N	1.27809230687028	5.48694449747673	9.20780695294268
C	0.02119323253358	7.62576591364162	9.54049050646729
H	0.42698757063097	3.78272461555851	12.78120308563238
C	0.50370703056345	2.13511814832851	11.40388296584809
C	2.48937645603847	3.29017570578301	12.44165841882729
C	1.76007863957511	5.63479016429701	7.85240510004498
C	-1.22574787062614	8.04242175427194	10.01216968889484
C	0.74725541321542	8.49709031665955	8.72827713594370
H	1.07590601057903	1.84134926365077	10.51895060826613
H	-0.53117506145830	2.27614606084262	11.08349001875544
C	0.59310417749922	1.03939189846384	12.46153270837574
C	2.57646995771346	2.19053665576820	13.49509444198937
H	2.84274857915659	4.24082029178072	12.84750779383328
H	3.13566727442905	3.04261801318456	11.59391900380554
H	1.91981044789919	4.64564670477261	7.43432468676775
H	2.69837948474926	6.18649666260239	7.82843245875984
H	1.01939175377365	6.16453372363901	7.25944714047191
H	-1.77498920231348	7.38023144157372	10.66808966661233
C	-1.73981178233653	9.27658534581417	9.65857842393304
C	0.22780215866724	9.73253159463269	8.37066118859440
H	1.74439244667188	8.23883008696894	8.39654352072059
H	-0.06486795651353	1.28904436398407	13.30074387679871
H	0.22119322103731	0.09850150198320	12.05054954777859
C	2.02072345791080	0.87084075714805	12.97065034796763
H	3.61297782375787	2.06753788162472	13.81599644110247
H	2.01393139322026	2.49401553501853	14.38443831117229
H	-2.70924362778763	9.57897813451016	10.03267091358236
C	-1.01948701650567	10.12524543748954	8.82818971917106
H	0.80961823194340	10.39504418166025	7.74309673181927
H	2.05587562660071	0.10841712459949	13.75200325595041
H	2.65585316530089	0.50847455772479	12.15525724126898
H	-1.42348689304022	11.08956821778790	8.54957470596126

Optimized Geometries of the Oxidized and Reduced Triazoline Selones

[MesPh1,5TrzSe]$^{\bullet+}$

Se	3.38879862963745	5.94792939007273	13.29418982425027
C	5.06095898124046	5.57855117815663	13.94260265013171
N	5.34937342947804	4.57757594621049	14.83426378046645
C	6.30571050068313	6.13058318892976	13.63887727095240
N	6.60530708752946	4.47747781644314	15.08181514396845
C	4.41348945575510	3.66814724255948	15.47849432720841
N	7.18833858360406	5.40302464251052	14.36845293718048
C	6.66022843181871	7.22968931419627	12.75881973569825
H	3.89791496226097	3.07937743651196	14.72478842467522
H	3.68696513121439	4.23928446103661	16.04969816455846
H	4.98044463111904	3.01879877662892	16.13704883954089
C	8.61440617204223	5.54080551018718	14.43978626314789
C	7.73685416772737	7.12405499787456	11.87561417140514
C	5.91008202813657	8.40714586476384	12.80021919604442
C	9.39500727582007	4.57675231742126	13.80747231421439
C	9.13990258427796	6.63171650391807	15.12694832004707
C	8.04755541897354	8.18126665304132	11.04073079415091
H	8.32017374886823	6.21546451076262	11.81867361092646
C	6.23474706873313	9.46305129968309	11.96787979181887
H	5.09228712807161	8.51152000180099	13.50018178150511
C	8.79417884117681	3.40437909647413	13.08883147845677
C	10.77113241900682	4.74124671785070	13.87304686213339
C	10.52200290698103	6.74390708793669	15.15827829658524
C	8.27164785297516	7.65100930619772	15.80133630817632
H	8.87341330655163	8.09121556701754	10.34903711554605
C	7.30056406386070	9.35064203651139	11.08678192200209
H	5.65758674113979	10.37611864038766	12.01059042630740
H	9.54655552922260	2.89592588879913	12.49019481460893
H	7.98752000124844	3.69176126364452	12.41100054674407
H	8.38966824942468	2.67104136869184	13.78794492551949
H	11.40697520308274	4.01046146758739	13.38814515231205
C	11.35207297808281	5.81446065154586	14.53994968081293
H	10.96384542809879	7.58092392296914	15.68566406781607
H	7.82129035175629	8.33428249510671	15.07878312804025
H	8.85763576289429	8.25494107640722	16.49063342108752
H	7.46677211051528	7.19677499202856	16.38054558111596
H	7.55089040994015	10.17639514428490	10.43477934582672
C	12.84121082287727	5.95538778547382	14.62009954584119
H	13.34524447220894	5.37362671144884	13.85065105060035
H	13.20695067974646	5.60559352660729	15.58694527558694
H	13.15158645221761	6.99402820031940	14.51370371298878

[PhDippTrzSe]$^{\bullet-}$

Se	5.81893107231308	16.83485203173554	3.84588377284609
C	4.84880807286365	15.24480252401664	3.88391382294477
N	3.51214472708868	15.02501373970122	4.27743790820973
C	5.29020987498956	14.01826891948198	3.50063260251992
N	3.12986875129739	13.72697502487404	4.09438876881109
C	2.52352184717884	15.89085208948333	4.71071529853201
C	6.62356482669420	13.57952755453993	3.06254418567308
N	4.26114477113403	13.09304671262728	3.70465278712793
C	1.18037991382806	15.48603143763425	4.60967402067453
C	2.79592174645022	17.13584906684110	5.29461639076776
C	7.43396097581405	12.84818695227009	3.95231667779572
C	7.07298709074461	13.84382035520081	1.76031026958615
C	4.05477050707368	11.96228123117754	2.84422153933199
H	0.95006160315118	14.52492065792043	4.17633515506438
C	0.16089004705194	16.30703808861421	5.05752369680318
C	1.75985585749906	17.94214535680284	5.72904414566207
H	3.81762735428830	17.46614774753619	5.40092193517981
C	7.01512389235678	12.66992674030469	5.39693491153165
C	8.66599215797408	12.37687327535945	3.51579294417633
C	8.31563371449610	13.35810154194732	1.36173948551135
C	6.23816481953594	14.62030924691751	0.76580731598994
H	4.98867674082880	11.41328317976120	2.73738969101507
H	3.70226650327977	12.25043215757365	1.84538246607320
H	3.31283929305821	11.30389552158012	3.29453528137523
H	-0.86386975031736	15.96760952737463	4.96030486880008
C	0.43158832051114	17.54694383491454	5.61805242204062
H	2.00328874744685	18.89971056130067	6.17370469579890
C	7.48414882179363	13.87829359563872	6.21088161690273
C	7.50285462732308	11.36690736694110	6.02526595757281
C	9.10583560230924	12.62482137171398	2.22574608149264
H	9.30238290164419	11.81878129979733	4.19127100403382
H	8.66933857419206	13.55637188660693	0.35632646398620
H	5.35632061249171	14.99211629092947	1.28829063990928
C	6.98616052322584	15.84195421707766	0.23722549322374
C	5.76985985925320	13.71744983019161	-0.37608777314441
H	-0.36952130847660	18.18644870535832	5.96625980937372
H	7.12669849869937	13.81197907530187	7.24126834030802
H	7.11685675552141	14.81185836923320	5.78193038199134
H	8.57575546330062	13.92643810178498	6.23490088374994
H	10.06931266565019	12.25190324659746	1.89934539659166
H	6.33791609467727	16.42874901531331	-0.41674482665346
H	7.28653513188047	16.47841333017348	1.06818774473747
H	7.87264371040902	15.56614358919124	-0.34006406767622
H	5.12595001607192	14.26934909069148	-1.06425046442150
H	6.61065857106189	13.32534987019354	-0.95338490167330
H	5.20199662935788	12.86445929501591	-0.00097807184244
H	5.92507779728840	12.65218319555306	5.43334953967678
H	8.58708682522959	11.34614382432678	6.15549708964523
H	7.22046125450680	10.49844704631690	5.42761013408504
H	7.06315689395798	11.24606330856033	7.01679646828832

[MesFcTrzSe]$^{\bullet-}$

Se	8.39488711459431	6.63312305331255	2.45457631754828
C	6.91848233795786	5.65214972327566	2.99943420750067
N	6.89895252045188	4.95146649981349	4.18593453398156
C	5.67435629108207	5.44255318036101	2.45822520344543
N	5.74153910979617	4.27473135906708	4.42200392562147
C	7.95749327358959	4.77546063589308	5.09813443811969
N	4.94829690265030	4.61347319870728	3.35039917740066
C	5.02972558930848	6.05099729229521	1.32536271336447
C	8.74567155268172	3.62946097156411	5.01072539135906
C	8.17521374716812	5.72781863794362	6.08945785518745
C	4.24275627621185	3.46233270388423	2.83198726334053
Fe	4.53321390554990	8.09127949699170	1.12961616452392
C	3.62152348041626	6.24866641002014	1.17756563003871
C	5.64389470657273	6.62376935650675	0.17533340857476
C	8.51672369233183	2.65217190075144	3.89987672549998
C	9.75247479508278	3.44159915466339	5.94806081610555
C	9.19613383241900	5.50744367705907	7.00704461851448
C	7.33939791236615	6.96826100605017	6.13699756871357
H	4.92185799166182	2.72126003768079	2.39196891999924
H	3.53252030951216	3.76987363378693	2.06715948487133
H	3.69280572461185	2.98860612774199	3.64488665433675
C	4.62938393749005	7.12216045645237	-0.68185222460065
C	3.37529705891614	6.88548102415435	-0.06765758947121
C	5.21291341243133	9.00022235816330	2.86157796918916
C	5.86958465684048	9.55014230508766	1.73605165779075
C	4.88632896620607	10.12141885628359	0.88825109129150
C	3.61697023952753	9.91841089405268	1.48939848180847
C	3.82214987301478	9.22306339432301	2.70993029339062
H	2.86996212674900	5.96269931511465	1.89952462643463
H	6.71066591800000	6.67197279126376	0.01622391532898
H	8.70440369326514	3.13129837430961	2.93679870274660
H	7.48369997980292	2.30282526975791	3.89133115773764
H	9.17691438358010	1.78910272662505	3.98924100098249
H	10.37116306523145	2.55195353098109	5.88509878685582
C	9.99308340692897	4.37215053783273	6.95196678131828
H	9.37275309454779	6.24529693208798	7.78300028555848
H	7.58242209263783	7.60586632826704	5.28331536154686
H	7.51888610848493	7.53184314337451	7.05308332200641
H	6.27639467648668	6.73361563310360	6.07753692210346
H	4.79087439088717	7.59906951034716	-1.63816526222219
H	2.40580561687428	7.14701766123835	-0.46707883530859
H	5.69678809632782	8.44887026396166	3.65475390286195
H	6.93148618576428	9.48345917445074	1.55168295701762
H	5.07001358665471	10.61510911543527	-0.05516700256655
H	2.66428693051356	10.23602460939945	1.09039685423843
H	3.05129290868396	8.90584362431870	3.39729413705379
C	11.11455447682247	4.16962602150220	7.93106839876375
H	12.06613832828854	4.51379776203063	7.51917215422294
H	11.23779818529385	3.11742985910308	8.19106406162091
H	10.94462353773121	4.72136046960807	8.85607707425202

[BnPhTrzSe]$^{\bullet-}$

Se	2.85681977179157	1.41336127373858	7.45964824441738
C	3.63808711428418	3.04149394436441	7.02450723949007
N	3.04908739017388	4.24954393818153	7.31125332475923
C	4.80122014528267	3.37607930723702	6.35855370316379
N	3.74107163012668	5.33094397493174	6.88816585191352
C	1.76139269156013	4.48721824123198	7.88753445535224
N	4.84059821697620	4.79276558127438	6.27011418962807
C	5.75121590760680	2.56756332784639	5.64557923344337
H	1.85655572701089	5.29198141246770	8.62225991878003
H	1.47540635970069	3.57473025313754	8.41353823195099
C	0.71357113427118	4.84909251658238	6.86417818931777
C	6.04954414228520	5.49291745435342	6.64720659749080
C	6.44278524785161	3.08782957901925	4.53402595405020
C	6.04232718842234	1.23998905016731	6.01089112758765
C	0.26206851983967	3.89193339788764	5.95758691294557
C	0.18909063868176	6.13332165788264	6.79990038212809
H	5.91084306894863	6.55819285950542	6.46415209744204
H	6.88963221031791	5.14567268708855	6.04913713895137
H	6.29980694875020	5.35052405850481	7.70542886785003
C	7.37467656113229	2.33457263262196	3.84189428582082
H	6.20502653974213	4.08574511760430	4.18715364472289
C	6.97088435215830	0.49450672937691	5.30933213867815
H	5.52440023749216	0.80405284882859	6.85346797077789
H	0.67988850562872	2.89132701502498	6.00039932433623
C	-0.70142927141156	4.21903210971477	5.01697802368102
H	0.55048214468299	6.88760157515956	7.48920977418214
C	-0.77822368073611	6.46255380502335	5.85960113938342
C	7.65564770576007	1.02897129127674	4.22196889809872
H	7.87634803539363	2.77006204781477	2.98510690924476
H	7.16919567237047	-0.52400676176213	5.62259429773700
H	-1.04313026393222	3.46585730112702	4.31790654393289
C	-1.22736827511597	5.50401835211114	4.96503944116907
H	-1.17352201739106	7.47027950651131	5.82111134696497
H	8.38592935728311	0.43929371291945	3.68207790942724
H	-1.97928965693920	5.75636820124453	4.22747669118055

[CyPhTrzSe]$^{\bullet-}$

Se	-1.07793759354078	5.81476441595104	12.53886751586990
C	-0.03279516341345	5.49708449184221	11.03875015464788
N	0.68006296002279	4.33511804687794	10.86301207996178
C	0.28575547706111	6.27106444641456	9.93673640887173
N	1.45720876194173	4.31728310806541	9.75624637089449
C	0.88139873727714	3.29441069102712	11.83516598513579
N	1.20823205448501	5.52518677295037	9.15614885087203
C	0.00260874546683	7.64274008362445	9.62406740253753
H	0.01010734570682	3.33716020789856	12.49550889637433
C	0.94004035038488	1.91729018722115	11.17844131786585
C	2.12920584587803	3.55891585596431	12.67944012253306
C	0.96086874915672	5.36432502785592	7.73836152824957
C	-1.15816980271093	8.30150632035833	10.07419803246209
C	0.89336665726513	8.39653496152763	8.83285710717831
H	1.76118795545056	1.88771336555336	10.45756059437141
H	0.02039674372362	1.74494957665046	10.61419821068309
C	1.13334566755346	0.82943939004937	12.23056864943960
C	2.33267316967588	2.46207489092189	13.71861824480557
H	2.01774381722384	4.53078226847215	13.16485876548425
H	2.99858604885270	3.62110577944192	12.01676845254022
H	0.03577742756476	4.81018579512119	7.53914728916321
H	1.79358464616034	4.81975138532063	7.29335032412923
H	0.88612287693177	6.33693284938179	7.25589854784748
H	-1.85958338184164	7.75935646174507	10.69256209806453
C	-1.40496465393775	9.61959951317477	9.74136868519930
C	0.63371861443893	9.71479891485188	8.50219646705133
H	1.82460252458193	7.94446409894130	8.51480959172532
H	0.25741573884112	0.79776350552091	12.88836938606256
H	1.19235132816632	-0.15122446440852	11.74961430205862
C	2.38068403261889	1.08139883883387	13.07222806970690
H	3.24661161551594	2.64663691388910	14.29020592240137
H	1.50788919021112	2.49451117841413	14.43912623395738
H	-2.31103474240987	10.09025179441851	10.10548409961582
C	-0.52110290164338	10.34481677330610	8.94686971387740
H	1.35173226635429	10.26063874861110	7.90033981800542
H	2.48872038426623	0.30442109239433	13.83498685036177
H	3.26661698541234	1.01325520124338	12.43079500340779
H	-0.72608847869241	11.37582151057225	8.68691290658615

[MesPhTrzSe]$^{\bullet-}$

Se	3.89378569128619	9.68294804526690	1.99537927445918
C	2.25283718581094	9.71987190360103	2.84797533834047
C	1.38305193059534	8.75075456146022	3.31215421831590
N	1.61384468695030	10.90648620714739	3.11511493203765
N	0.25543112034121	9.42324566165173	3.85583808776338
C	1.38207162621909	7.32381726411721	3.14713941607563
N	0.41555267856469	10.78160108991507	3.74388188914660
C	2.06621048274490	12.20995093832194	2.82564374563659
C	-0.20929175859090	9.06076807591701	5.17854067402574
C	0.17126720314646	6.60399910184868	3.12229143280763
C	2.56790753904595	6.57897457016366	3.00379009310077
C	2.77524059190301	12.91283314805622	3.79778460665853
C	1.76692438028576	12.77920735079922	1.59334444626087
H	-1.10748289485050	9.63412500960967	5.40656756619311
H	0.54181299394492	9.26605039807871	5.95102261104616
H	-0.45504531681845	8.00143521757258	5.21514808639471
C	0.14861289382687	5.22751241965919	2.98114238949747
H	-0.76467643895411	7.14660313996906	3.17021381584937
C	2.53324996775323	5.20519523215875	2.85852437812950
H	3.51482995863163	7.10067092842589	3.00313702770328
C	3.12471498993353	12.24416585105014	5.09068674988670
C	3.16939841526163	14.21375062949931	3.51999281978608
C	2.18011841819766	14.08516616597507	1.35052845488801
C	1.04661168717201	11.97862639840870	0.55413741712498
H	-0.80545128485651	4.71309618980395	2.95700328716116
C	1.32877424056277	4.50761632680023	2.85240292136706
H	3.46766565428075	4.66614834548254	2.75309088510655
H	2.23660835495229	11.84133613016726	5.57881299328367
H	3.61357111681871	12.93731889556278	5.77576679221654
H	3.80090910685450	11.40719302310863	4.90349325716475
H	3.72516863167308	14.76731634053563	4.27038307125090
C	2.87920902086754	14.81598893015896	2.30036918085200
H	1.95409521989080	14.53737782641420	0.39038168836575
H	0.79079157380170	12.59213518103520	-0.31026358155166
H	0.13021939062032	11.54374432547193	0.95358801453077
H	1.67904061116981	11.15294981507987	0.22010915552181
H	1.31187263344625	3.43042475880763	2.74362410233823
C	3.33039005798235	16.22151437423645	2.01992209805812

[MesPh1,5TrzSe]$^{\bullet-}$

Se	3.30993084232187	6.39274130425050	14.03654314685968
C	5.04780097115381	5.77445905744603	14.22214847777509
N	5.34649126913793	4.59420386892302	14.84584133623020
C	6.27385399513374	6.25924827000322	13.77676415470703
N	6.65444809136206	4.26310614209349	14.85242814938967
C	4.43665939006826	3.62260159341021	15.36467673687723
N	7.22994330321907	5.31359270580838	14.19976573391272
C	6.58725088749055	7.32706756389408	12.86593027415307
H	4.30027880384839	2.79038558084255	14.66823035564198
H	3.47446692864708	4.10059469671866	15.52832789653359
H	4.82312263220650	3.22929435766724	16.30557214541981
C	8.60975027942231	5.46962640704426	14.34450286957275
C	7.73012047481852	7.28770147482103	12.04054196279841
C	5.77226641033853	8.47296526190858	12.76400635319102
C	9.45423110891824	4.46535972501289	13.84272744457169
C	9.15467319562696	6.60489777042926	14.96392541149322
C	8.03382906702185	8.32531413445119	11.17713361194131
H	8.38339796242958	6.42652515014879	12.05651152407042
C	6.08401732543163	9.49916708641189	11.89360945052385
H	4.88714175729646	8.54156884192751	13.38001780839784
C	8.88576424830101	3.26923309761849	13.14147112491922
C	10.82759655048063	4.62048243482632	13.96138505241394
C	10.53721086724116	6.72516869147580	15.03049587699387
C	8.29080901715402	7.65688041549460	15.59245452466271
H	8.92000822016135	8.24924105128735	10.55767724630235
C	7.21980005818507	9.44653652927289	11.09064524819287
H	5.42989505295702	10.36220520350348	11.84786083192588
H	9.67806771091516	2.67495605248829	12.68530763044718
H	8.19088532406612	3.57483110615800	12.35650211718626
H	8.31698071449040	2.63584322055772	13.82145743415030
H	11.47498801663797	3.84535215185740	13.56301355223434
C	11.39488887106377	5.74847826978090	14.54212904689127
H	10.95519516322343	7.60642266696242	15.50739600755168
H	7.87429616705942	8.34019645077468	14.85115054788334
H	8.87254773201483	8.24706891590710	16.30165785369730
H	7.44790148868454	7.21896168179426	16.12590154529724
H	7.46225162818956	10.25810663918543	10.41614065556631
C	12.88443455637192	5.89119204002170	14.67077096111317
H	13.40972311437520	5.28035613283392	13.93553322213850
H	13.23629904694661	5.58008682385045	15.65797431313463
H	13.20407175558742	6.92431943113596	14.52737036323700

[PhPhTrzSe]$^{\bullet-}$

Se	1.52665744176102	1.80635330374319	6.24654251526088
C	2.42460672345281	3.42438591380426	6.17856991439667
C	3.75494334321027	3.68609728441496	5.94203817660085
N	1.85244678101890	4.69088172274864	6.34778184983527
C	0.53313213941184	5.07691540695241	6.55357192263614
C	4.90233802464155	2.81106989235484	5.87628102584001
N	3.93291003236332	5.08543641856778	5.95282585774578
N	2.74300104319108	5.70404031221938	6.16434137209529
C	-0.37150694379481	4.31512580945218	7.30057189140014
C	0.10832498582483	6.31281098350862	6.04640960511656
C	4.84296326000209	1.51527354532647	5.33968693270620
C	6.14865233582098	3.24999527409249	6.35709033280232
C	4.69784489658349	5.72970784880914	4.90673936764163
H	-0.05896683124441	3.37363245283306	7.72386550137900
C	-1.66493035289959	4.76839188403777	7.49412895390720
H	0.80539803020731	6.91891444093779	5.48713271120253
C	-1.18604006256318	6.75373286486244	6.25872676265676
H	3.89816311502129	1.14598401190519	4.96815878985861
C	5.97092335331882	0.71518049945993	5.28780408750679
C	7.27294383491289	2.44523218739514	6.29681063637611
H	6.21872011815316	4.22619887897041	6.82095689048370
H	4.23376050077765	5.60529905459215	3.92168613168391
H	5.70404370517864	5.31867339326936	4.86668036075170
H	4.76580561724006	6.79422041559818	5.12813860579316
C	-2.09074765480662	5.98543480619419	6.97737229024319
H	-2.34743047233382	4.15843295162857	8.07336865324102
H	-1.48956191415051	7.71010467770718	5.84966250251729
H	5.88969866535883	-0.27929235302319	4.86511584356129
C	7.19778548103426	1.16768065498189	5.75862080560199
H	8.21334206493084	2.81545423721095	6.68835967621943
H	-3.10383705619127	6.33107544443211	7.13971549530248
H	8.07582579456784	0.53571578101192	5.71103453763518

Optimized Geometry of the reduced gold complex [PhDippAuPh]$^{\bullet-}$

Au	1.75924886678665	10.54099638099388	6.98309714516514
C	1.42684328436678	10.59677086395858	4.95522663158894
C	2.19601516297056	10.61597563725110	8.97977453059423
N	0.59362862285739	9.87498267856263	4.07103758197653
C	2.07989585024433	11.47774783657926	4.12692520324377
C	3.17127920943686	11.50857584658682	9.48297575411741
C	1.55904469408942	9.79818539253725	9.94146712251744
N	0.69950785646869	10.29204137657143	2.74687770645928
C	-0.29331086450196	8.84367318644461	4.35372140239416
N	1.67772640054889	11.25036447936842	2.81814320307923
C	3.08925796681061	12.49989349648703	4.47330473715524
H	3.69767452635322	12.16706732467786	8.78674298404059
C	3.49020173949603	11.58242641279450	10.84317698086232
C	1.86881234129852	9.86235906354293	11.30440382682291
H	0.79520944216274	9.08770706682131	9.61431530931459
C	-1.05867055904948	8.26825722227103	3.30508362408799
C	-0.45684463505579	8.34557907302691	5.66646775625138
C	1.83195620601454	12.13094355488127	1.69074060860607
C	4.45784510407533	12.15066254244848	4.50378118391995
C	2.67227135482295	13.80807744306943	4.80463857499753
H	4.25203008170485	12.28879035165119	11.18633619395558
C	2.83934420317785	10.75736328960307	11.76601867299577
H	1.34952439460302	9.20946676175706	12.01231879265089
H	-0.93329217637842	8.64923472971034	2.29415245378056
C	-1.94982007781804	7.23527003442447	3.58044473572599
C	-1.35280442063852	7.31364298278789	5.91570247000217
H	0.12970285243204	8.78315213023705	6.47840911813299
H	2.71510612870225	12.75781739134856	1.86205021280010
H	0.94514842784659	12.77995274647076	1.55355367391923
H	1.97168161028667	11.53825091370271	0.77483800338869
C	4.90208881575535	10.72340362484471	4.23674676144906
C	5.39657693376010	13.12845969781825	4.86163047262287
C	3.63959591601665	14.75869984502466	5.15683935254424
C	1.19547079908653	14.15738982220456	4.86034024728009
H	3.08467698985037	10.81107429049291	12.82951595460830
H	-2.52975176774138	6.80676130244743	2.75886262218747
C	-2.11383644462525	6.74130852382422	4.88140903173481
H	-1.45902962254972	6.94800703676472	6.93948064135469
H	4.03040166945793	10.18397698399044	3.83789888542527
C	5.29387684229350	10.03743367593345	5.55625611204737
C	6.02959884545683	10.64131957230667	3.19786119707566
H	6.45577038760216	12.86708201135431	4.90778089726639
C	4.99311303548416	14.42390808402483	5.18009569176540
H	3.32887438365044	15.76817626090926	5.43338234007515
H	0.64963088666255	13.32794739498963	4.38521584324743
C	0.72553248388413	14.22018243055694	6.32309290395655
C	0.86148980776056	15.44788763410479	4.09937910042771
H	-2.81537814897645	5.93133879379036	5.08691842012618
H	5.57272891526910	8.98702244104503	5.38000182777140
H	4.44884613770891	10.05778066247940	6.26186758369422
H	6.14917303343905	10.54735065357539	6.02704722301229
H	6.27128750134736	9.59017335319279	2.97890002773889
H	6.95122767066750	11.12414511882871	3.55852868297525
H	5.73935646144162	11.13062944752039	2.25629209156960
H	5.73586873028717	15.17299351389064	5.46345848100160
H	-0.35507717349367	14.42436162481522	6.37608619107032
H	1.25529569901461	15.01500323895974	6.87144615394006
H	0.92342807044489	13.26239664662022	6.82896068006553
H	-0.22521490343427	15.62167956126235	4.10073153049406
H	1.19973067819861	15.39351300441463	3.05377669732728
H	1.33691377216580	16.32694553744406	4.56130015959746

Table S12. Selected orbital compositions for CyPhTrzSe und CyPhTrz.

Selenium adducts	Carbene
LUMO+2: -0.462 eV 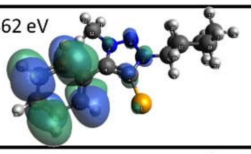	0.043 eV 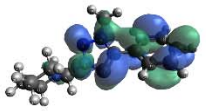
LUMO+1: -0.656 eV 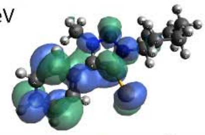	-0.269 eV 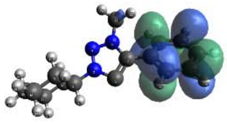
LUMO: -1.308 eV 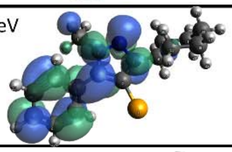	-0.978 eV 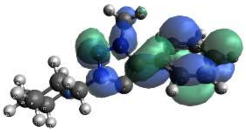
HOMO: -5.257 eV 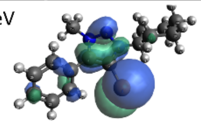	-5.391 eV 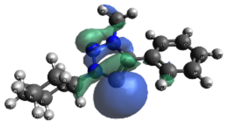
HOMO-1: -5.506 eV 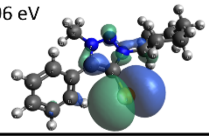	-6.019 eV 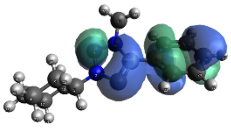
HOMO-2: -7.028 eV 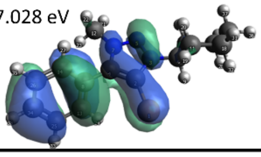	-6.961 eV 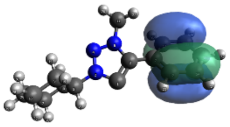

Geom. Opt.: PBEh-3c
Single Point: B3LYP,
RIJCOSX, D3, def2-TZVP
def2/J ZORA

Table S13. HOMO and LUMO compositions of the triazoline selones.

Compound	HOMO	LUMO
PhPhTrzSe	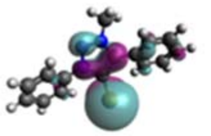	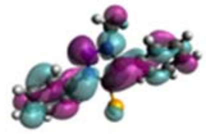
DippPhTrzSe	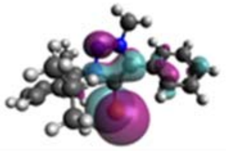	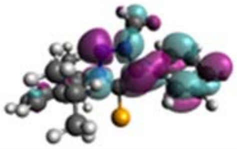
PhDippTrzSe	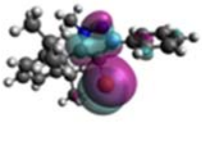	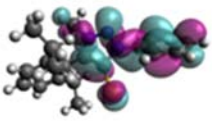
BnPhTrzSe	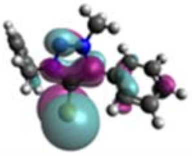	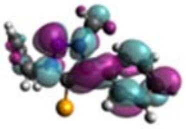
CyPhTrzSe	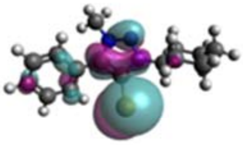	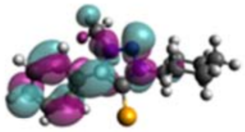
MesPhTrzSe	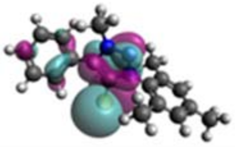	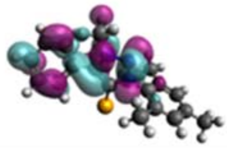
MesPh1,5TrzSe	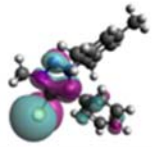	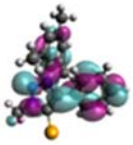
PhCF$_3$PhTrzSe	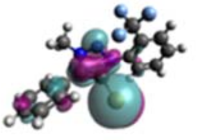	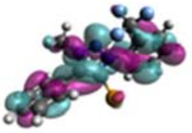
MesFcTrzSe	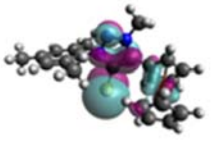	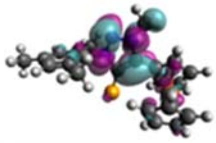
bicaSe$_2$	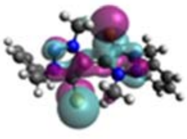	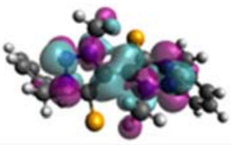

Table S14. HOMO and LUMO compositions of the triazolylidenes.

Compound	HOMO	LUMO
PhPhTrz	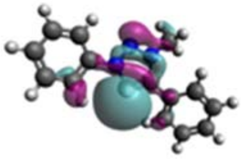	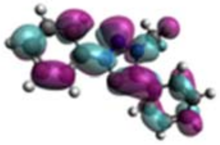
DippPhTrz	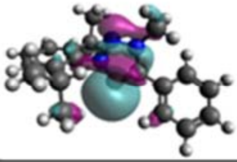	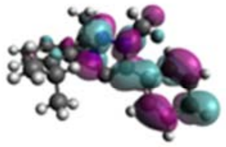
PhDippTrz	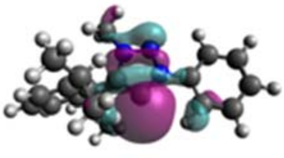	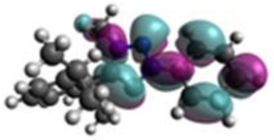
BnPhTrz	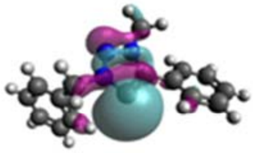	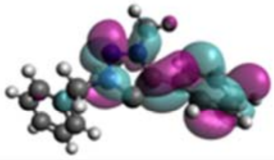
CyPhTrz	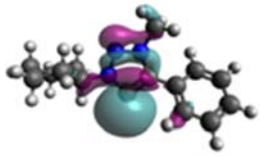	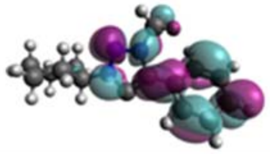
MesPhTrz	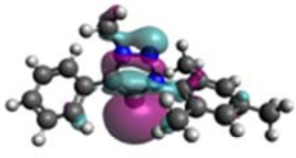	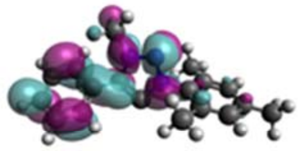
MesPh1,5Trz	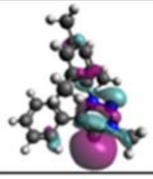	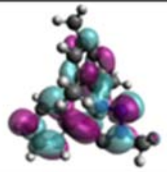
$PhCF_3PhTrz$	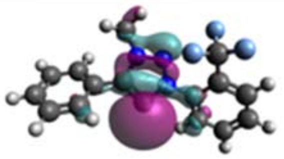	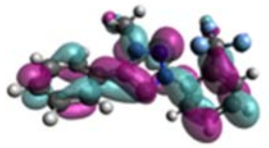
MesFcTrz	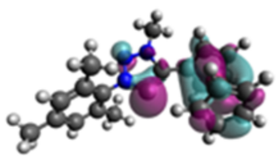	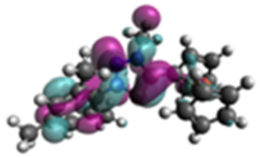
bica	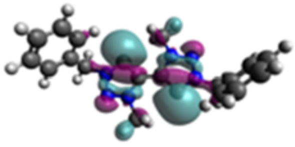	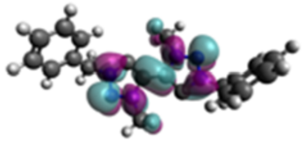

Table S15. Spin population plots of the reduced triazoline selones (iso value 0.004).

Compound	Spin population plot
[PhDippTrz]$^{\bullet-}$	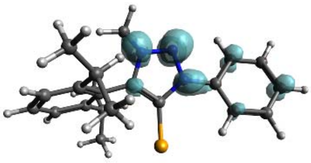
[BnPhTrz]$^{\bullet-}$	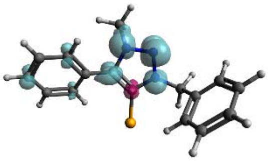
[CyPhTrz]$^{\bullet-}$	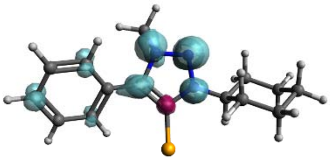
[MesPhTrz]$^{\bullet-}$	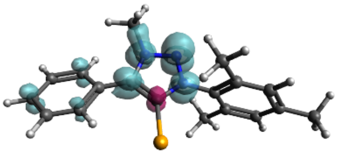
[MesPh1,5Trz]$^{\bullet-}$	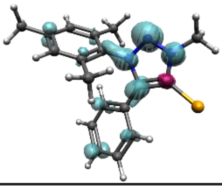
[MesFcTrz]$^{\bullet-}$	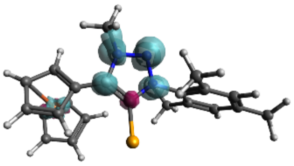

Table S16. Bending of the C^{methyl}-N bond out of the triazoline ring plane in the optimized structures of the reduced triazoline selones. The described angle is close to 90° (88-90°) for the neutral species of all analyzed triazoline selones.

	Angle of the normal of the triazoline ring plane to the C^{methyl}-N bond (α)
$[BnPhTrzSe]^{•–}$	49°
$[PhDippTrzSe]^{•–}$	62°
$[MesFcTrzSe]^{•–}$	50°
$[MesPh1,5TrzSe]^{•–}$	85°
$[MesPhTrzSe]^{•–}$	48°
$[PhPhTrzSe]^{•–}$	52°
$[CyPhTrzSe]^{•–}$	48°

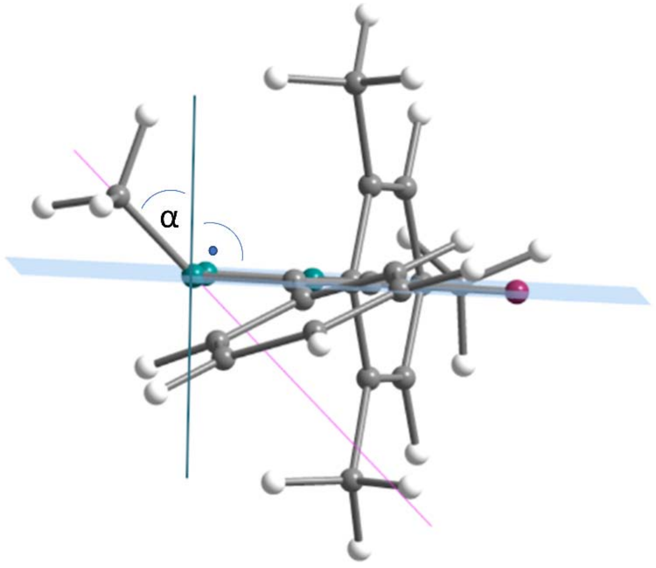

Figure S315. Determination of α in the DFT optimized structures of the reduced triazoline selones.

9 Table of Data

Table S17. Various properties of the triazolylidenes and their selenium adducts.

	E_{sol}^{LUMO}(TrzSe) in MeCN	E_{sol}^{HOMO}(TrzSe) in MeCN	E_{g}^{LUMO}(TrzSe)	$E^{1/2}_{red}$ (50 V/s)	E^{p}_{red} (100 mV/s)	E^{p}_{ox} (100 mV/s)	Mulliken charges on Se
	/eV	/eV	/eV	/V	/V	/V	/a.u.
**PhDippTrz**	-1.518	-5.275	-1.266	-2.455	-2.490	0.323	-0.54928
**MesPhTrz**	-1.433	-5.314	-1.082	-2.527	-2.559	0.091	-0.55389
**BnPhTrz**	-1.409	-5.289	-1.061	-2.558	-2.504	-0.087	-0.56633
**CyPhTrz**	-1.308	-5.257	-0.978	-2.656	-2.642	0.047	-0.57788
**PhCF$_3$PhTrz**	-1.636	-5.351	-1.449	-2.396	-2.440	0.370	-0.54276
**MesPh1,5Trz**	-1.507	-5.352	-1.124	-2.494	-2.509	-0.011	-0.56914
**MesFcTrzSe**	-1.216	-5.158	-0.743	-2.642	-2.626	0.035	-0.55321
**PhPhTrz**	-1.571	-5.280	-1.392	-2.415	-2.426	0.105	-0.55519
**DippPhTrz**	-1.454	-5.265	-1.138	-2.523	-2.518	0.252	-0.54373

10 References

[1] J. Beerhues, H. Aberhan, T.-N. Streit, B. Sarkar, *Organometallics* **2020**, *39*, 4557–4564.

[2] K. J. Kilpin, U. S. D. Paul, A.-L. Lee, J. D. Crowley, *Chem. Comm.* **2011**, *47*, 328–330.

[3] G. R. Fulmer, A. J. M. Miller, N. H. Sherden, H. E. Gottlieb, A. Nudelman, B. M. Stoltz, J. E. Bercaw, K. I. Goldberg, *Organometallics* **2010**, *29*, 2176–2179.

[4] M. Krejčik, M. Daněk, F. Hartl, *J. Electroanal. Chem.* **1991**, *317*, 179–187.

[5] S. Stoll, A. Schweiger, *J. Magn. Reson.* **2006**, *178*, 42–55.

[6] a) Bruker AXS Inc, *SAINT+*: Data Integragtion Engine, Madison, Wisconsin, USA, **1997 - 2002**; b) Bruker AXS Inc, *APEX3*, Madison, Wisconsin, USA, **2015**; c) G. M. Sheldrick, *SADABS Version 2008/1*: Program for Empirical Absorption Correction, University of Göttingen, Germany, **2008**; d) G. M. Sheldrick, *SHELXL Version 2014/7*: Program for Chrystal Structure Solution and Refinement, University of Göttingen, Germany, **2014**; e) C. B. Hübschle, G. M. Sheldrick, B. Dittrich, *J. Appl. Crystallogr.* **2011**, *44*, 1281–1284; f) G. M. Sheldrick, *Acta Crystallogr. C* **2015**, *71*, 3–8.

[7] a) H. Kim, J. Park, Y. S. Lee, *J. Comput. Chem.* **2015**, *36*, 33–41; b) M. Namazian, P. Norouzi, *J. Electroanal. Chem.* **2004**, *573*, 49–53; c) A. Kuhn, K. G. Eschwege, J. Conradie, *J. Phys. Org. Chem.* **2012**, *25*, 58–68; d) M. J. S. Dewar, J. A. Hashmall, N. Trinajstic, *J. Am. Chem. Soc.* **1970**, *92*, 5555–5559; e) P. I. Djurovich, E. I. Mayo, S. R. Forrest, M. E. Thompson, *Org. Electron.* **2009**, *10*, 515–520; f) V. D. Parker, *J. Am. Chem. Soc.* **1974**, *96*, 5656–5659; g) V. D. Parker, *J. Am. Chem. Soc.* **1976**, *98*, 98–103; h) X.-Q. Zhu, C.-H. Wang, *J. Org. Chem.* **2010**, *75*, 5037–5047.

[8] a) M. Nič, J. Jirát, B. Košata, A. Jenkins, A. McNaught, *IUPAC Compendium of Chemical Terminology;* IUPAC, Research Triagle Park, NC, **2009**; b) S. Trasatti, *Pure Appl. Chem.* **1986**, *58*, 955–966.

[9] P. Zanello, *Inorganic Electrochemistry*: Theory, Practice and Application; Royal Society of Chemistry, Cambridge, **2003**.

[10] N. G. Connelly, W. E. Geiger, *Chem. Rev.* **1996**, *96*, 877–910.

[11] a) J. R. Levin, W. L. Dorfner, A. X. Dai, P. J. Carroll, E. J. Schelter, *Inorg. Chem.* **2016**, *55*, 12651–12659; b) I. V. Kuvychko, J. B. Whitaker, B. W. Larson, T. C. Folsom, N. B. Shustova, S. M. Avdoshenko, Y.-S. Chen, H. Wen, X.-B. Wang, L. Dunsch, A. A. Popov, O. V. Boltalina, S. H. Strauss, *Chem. Sci.* **2012**, *3*, 1399; c) P. Peljo, H. H. Girault, *Energy Environ. Sci.* **2018**, *11*, 2306–2309.

[12] a) F. Neese, *WIREs Comput. Mol. Sci.* **2012**, *2*, 73–78; b) F. Neese, *WIREs Comput. Mol. Sci.* **2018**, *8*, 33.

[13] S. Grimme, J. G. Brandenburg, C. Bannwarth, A. Hansen, *J. Chem. Phys.* **2015**, *143*, 54107.

[14] a) Becke, *Physical review. A, General physics* **1988**, *38*, 3098–3100; b) Perdew, *Physical review. B, Condensed matter* **1986**, *33*, 8822–8824; c) J. P. Perdew, *Physical review. B, Condensed matter* **1986**, *34*, 7406.

[15] F. Weigend, R. Ahlrichs, *Phys. Chem. Chem. Phys.* **2005**, *7*, 3297–3305.

[16] A. D. Becke, *J. Chem. Phys.* **1993**, *98*, 5648–5652.

[17] a) S. Grimme, *J. Comput. Chem.* **2004**, *25*, 1463–1473; b) S. Grimme, *J. Comput. Chem.* **2006**, *27*, 1787–1799; c) S. Grimme, J. Antony, S. Ehrlich, H. Krieg, *J. Chem. Phys.* **2010**, *132*, 154104; d) S. Grimme, S. Ehrlich, L. Goerigk, *J. Comput. Chem.* **2011**, *32*, 1456–1465.

[18] C. van Wüllen, *J. Chem. Phys.* **1998**, *109*, 392–399.

[19] a) M. Cossi, N. Rega, G. Scalmani, V. Barone, *J. Comput. Chem.* **2003**, *24*, 669–681; b) Y. Takano, K. N. Houk, *J. Chem. Theory Comput.* **2005**, *1*, 70–77.

[20] a) A. Klamt, G. Schüürmann, *J. Chem. Soc, Perkin Trans. 2* **1993**, 799–805; b) S. Sinnecker, A. Rajendran, A. Klamt, M. Diedenhofen, F. Neese, *J. Phys. Chem. A* **2006**, *110*, 2235–2245.

[21] P.-O. Löwdin, *J. Chem. Phys.* **1950**, *18*, 365–375.

[22] M. D. Hanwell, D. E. Curtis, D. C. Lonie, T. Vandermeersch, E. Zurek, G. R. Hutchison, *J. Cheminform.* **2012**, *4*, 17.
